# Soy protein supplementation is not androgenic or estrogenic in college-aged men when combined with resistance exercise training

**DOI:** 10.1038/s41598-018-29591-4

**Published:** 2018-07-24

**Authors:** Cody T. Haun, C. Brooks Mobley, Christopher G. Vann, Matthew A. Romero, Paul A. Roberson, Petey W. Mumford, Wesley C. Kephart, James C. Healy, Romil K. Patel, Shelby C. Osburn, Darren T. Beck, Robert D. Arnold, Ben Nie, Christopher M. Lockwood, Michael D. Roberts

**Affiliations:** 1 Molecular and Applied Sciences Laboratory, School of Kinesiology, Auburn University, Auburn, AL USA; 2 Department of Cell Biology and Physiology, Edward Via College of Osteopathic Medicine – Auburn Campus, Auburn, AL USA; 3 Lockwood, LLC, Draper, UT 84020 USA; 4 Department of Drug Discovery & Development, Harrison School of Pharmacy, Auburn University Pharmaceutical Research Building, Auburn, AL USA

## Abstract

It is currently unclear as to whether sex hormones are significantly affected by soy or whey protein consumption. Additionally, estrogenic signaling may be potentiated via soy protein supplementation due to the presence of phytoestrogenic isoflavones. Limited evidence suggests that whey protein supplementation may increase androgenic signalling. Therefore, the purpose of this study was to examine the effects of soy protein concentrate (SPC), whey protein concentrate (WPC), or placebo (PLA) supplementation on serum sex hormones, androgen signaling markers in muscle tissue, and estrogen signaling markers in subcutaneous (SQ) adipose tissue of previously untrained, college-aged men (n = 47, 20 ± 1 yrs) that resistance trained for 12 weeks. Fasting serum total testosterone increased pre- to post-training, but more so in subjects consuming WPC (p < 0.05), whereas serum 17β-estradiol remained unaltered. SQ estrogen receptor alpha (ERα) protein expression and hormone-sensitive lipase mRNA increased with training regardless of supplementation. Muscle androgen receptor (AR) mRNA increased while ornithine decarboxylase mRNA (a gene target indicative of androgen signaling) decreased with training regardless of supplementation (p < 0.05). No significant interactions of supplement and time were observed for adipose tissue ERα/β protein levels, muscle tissue AR protein levels, or mRNAs in either tissue indicative of altered estrogenic or androgenic activity. Interestingly, WPC had the largest effect on increasing type II muscle fiber cross sectional area values (Cohen’s d = 1.30), whereas SPC had the largest effect on increasing this metric in type I fibers (Cohen’s d = 0.84). These data suggest that, while isoflavones were detected in SPC, chronic WPC or SPC supplementation did not appreciably affect biomarkers related to muscle androgenic signaling or SQ estrogenic signaling. The noted fiber type-specific responses to WPC and SPC supplementation warrant future research.

## Introduction

Both whey and soy protein supplementation augment gains in muscle mass beyond resistance exercise training alone^1^. There is evidence whey or soy protein supplementation may affect sex hormone signaling mechanisms, and these mechanisms may contribute to observed changes in body composition. For instance, cell culture experiments have revealed soy-derived isoflavones reduce androgen receptor (AR) content in different cell lines^2,3^, and Weber *et al*. reported plasma testosterone decrements in rodents fed high amounts of isoflavones^4^. Regarding whey protein supplementation, Hulmi *et al*. reported ingestion following one bout of resistance exercise blunts increases in serum testosterone^5^. Notably, these authors suggested this observation could be due to an increase in muscle tissue testosterone uptake following exercise. Given that enhanced muscle AR signaling may play a role in the hypertrophic response to resistance exercise, and controversial data exist regarding the specific effects of soy and whey protein on androgenic signaling, it is plausible that whey or soy-induced alterations in androgenic signaling may differentially influence the adaptive response to resistance exercise training.

While whey and soy protein have been shown to promote anabolic effects in skeletal muscle, the effects of these protein supplements on adipose tissue physiology have been less studied. Daidzein and genistein compose the majority of soy isoflavone content in most soy-based food sources, with differences depending on the specific source^6^. These compounds are known ligands of the estrogen alpha (ERα) and beta (ERβ) receptors^7^. Notably, ERs are major regulators of adipocyte size^8^, and high genistein concentrations in culture (>1 μM) induce adipogenesis and result in significant increases in adipocyte number^9^. Hence, it stands to reason that soy protein supplementation may increase whole-body adiposity, and that this increase in adiposity may occur in part through isoflavone-mediated ER signaling.

We recently reported the effects of five different supplements consumed during 12 weeks of resistance exercise training on body composition, strength, muscle fiber cross sectional area (fCSA), satellite cell number, and subcutaneous (SQ) adipocyte CSA (aCSA). Detailed information about supplement formulation, contents, and training design can be found in the paper by Mobley *et al*.^10^. Herein, we sought to analyze remaining biological samples from participants in our previous study whom were in the soy protein concentrate (SPC), whey protein concentrate (WPC), and maltodextrin placebo (PLA) groups to examine the influence of these supplements on androgen and estrogen signaling markers in serum, vastus lateralis muscle tissue, and SQ adipose tissue. We previously reported no significant differences between SPC, WPC and PLA in body fat alterations or muscle hypertrophy responses from the 12-week intervention^10^. However, we did not examine or report the influence of the dependent variables from this investigation on muscle fCSA, SQ aCSA, or body composition alterations independent of group in our previous work. Thus, a secondary aim of this investigation was to examine the influence of each dependent variable herein on changes in SQ fCSA and muscle fCSA. Null hypotheses, assuming no differences between groups and no significant relationships between variables, were posed for all analyses.

## Results

A total of 47 subjects were distributed in groups as: PLA (n = 15), SPC (n = 15), WPC (n = 17). Due to either a lack of subject compliance, biological sample availability, or datum removal procedures described in the methods, the average sample size for analysis was n = 33 (i.e., approximately 11 per group). Specific sample sizes for each analysis are described in supplementary Tables and/or Figures. First, data are shown from our previous work in Tables 1–3 describing changes in body composition, SQ aCSA, and muscle fCSA. No significant group × time interaction was identified for these variables.

**Table 1:** Subject and Body Mass Data.

	Age (years)	Total Body Mass (kg)		Total Fat Mass (kg)		Total Body Muscle Mass (kg)	
		Pre	Post	Pre	Post	Pre	Post
*PLA*	20.85 ± 1.14	78.00 ± 10.29	81.48 ± 9.83	18.06 ± 5.19	18.80 ± 5.38	57.05 ± 6.33	59.90 ± 6.22
*SPC*	20.85 ± 1.72	81.37 ± 13.84	84.13 ± 14.50	19.73 ± 8.45	20.51 ± 8.51	57.67 ± 6.76	60.42 ± 7.35
*WPC*	20.87 ± 1.60	79.57 ± 12.94	81.83 ± 12.84	19.47 ± 8.58	18.69 ± 8.25	58.15 ± 5.74	60.81 ± 5.33
*Total*	20.85 ± 1.48	79.64 ± 12.23	82.45 ± 12.29	19.10 ± 7.49	19.31 ± 7.43	57.65 ± 6.12	60.40 ± 6.16

**Table 2:** Adiposity Distribution, and aCSA Data.

	Visceral Adiposity (kg)		Android Adiposity (kg)		Gynoid Adiposity (kg)		aCSA (μm^2^)	
	Pre	Post	Pre	Post	Pre	Post	Pre	Post
*PLA*	0.27 ± 0.20	0.26 ± 0.21	1.35 ± 0.58	1.40 ± 0.66	3.23 ± 0.97	3.31 ± 0.96	3172.89 ± 1268.45	2765.55 ± 1680.46
*SPC*	0.42 ± 0.37	0.44 ± 0.48	1.55 ± 0.89	1.60 ± 0.95	3.42 ± 1.59	3.49 ± 1.46	3540.94 ± 1405.45	3340.39 ± 1586.78
*WPC*	0.48 ± 0.35	0.45 ± 0.32	1.52 ± 0.90	1.42 ± 0.87	3.39 ± 1.60	3.10 ± 1.64	3819.91 ± 965.70	3552.34 ± 1027.82
*Total*	0.40 ± 0.32	0.39 ± 0.35	1.47 ± 0.80	1.47 ± 0.82	3.35 ± 1.40	3.29 ± 1.38	3510.52 ± 1221.59	3216.47 ± 1460.18

**Table 3:** VL Thickness, Leg Lean Mass, and fCSA Data.

	VL Thickness (cm)		Dual Leg Lean Mass (kg)		Type I fCSA (μm^2^)		Type II fCSA (μm^2^)	
	Pre	Post	Pre	Post	Pre	Post	Pre	Post
*PLA*	2.26 ± 0.30	2.72 ± 0.32	21.90 ± 2.81	23.43 ± 2.86	3449.24 ± 975.28	3972.24 ± 2243.75	4658.66 ± 1110.54	5093.30 ± 1116.52
*SPC*	2.62 ± 0.41	3.04 ± 0.34	22.46 ± 2.85	23.90 ± 3.05	3937.71 ± 864.35	4698.13 ± 1329.59	4535.50 ± 967.69	5182.24 ± 1422.97
*WPC*	2.49 ± 0.23	2.96 ± 0.29	22.55 ± 2.59	23.93 ± 2.32	3583.41 ± 870.45	3975.84 ± 1069.78	4760.92 ± 1415.50	6287.67 ± 2348.93
*Total*	2.46 ± 0.34	2.91 ± 0.34	22.32 ± 2.69	23.76 ± 2.68	3659.98 ± 903.64	4215.18 ± 1205.67	4654.13 ± 1162.34	5549.51 ± 1789.50

**Figure 1:**
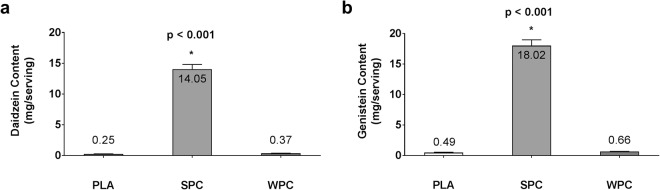
Supplement isoflavone content in milligrams (mg) per serving are displayed for each supplement based on LC-MS/MS analyses. Panel a visualizes daidzein content and panel b genistein content. Mean phytoestrogen values are provided above or within bars and error bars represent standard error of the mean. Symbol: *denotes SPC > PLA and WPC (p < 0.001).

Isoflavone contents of each supplement are shown in Fig. 1. Five, randomly selected supplement packets from SPC, WPC, and PLA were used for LC-MS/MS analysis (n = 15). Independent samples t-tests wherein each supplement was directly compared to another revealed SPC contained significantly more isoflavones than both WPC and PLA (p < 0.001). SPC contained 14.05 ± 0.74 mg of daidzein and 18.02 ± 0.95 mg of genistein, whereas WPC and PLA contained negligible amounts of both phytoestrogens (<1 mg).

### Serum Hormones

**Figure 2:**
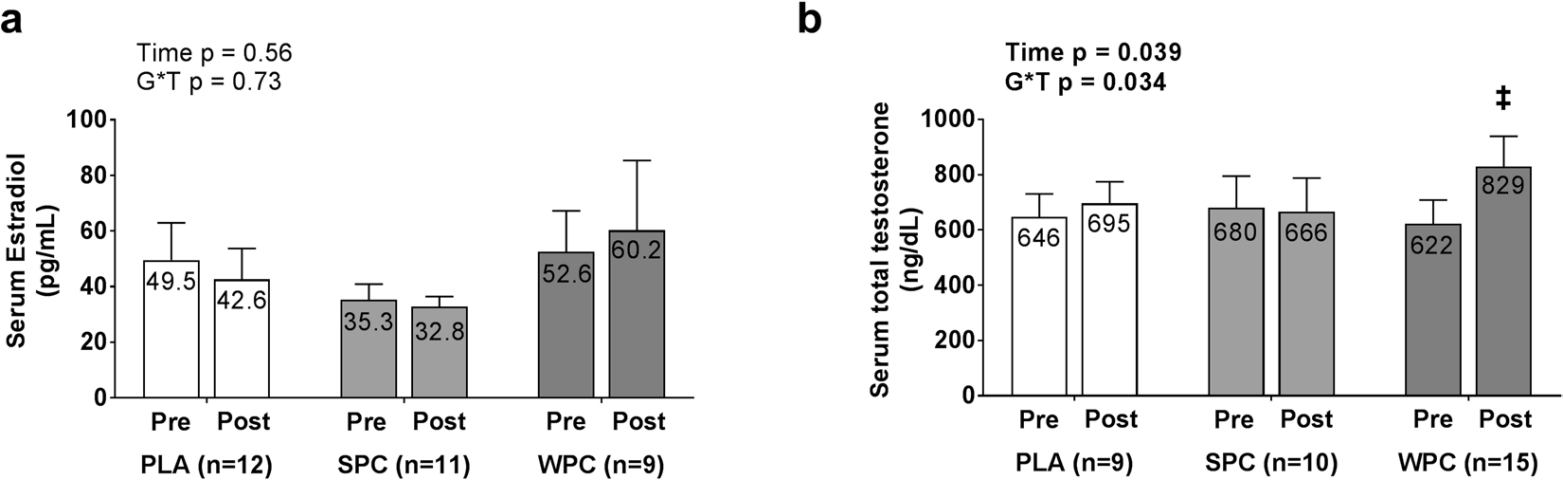
Resting, fasting serum estradiol concentrations are displayed in picograms (pg) per milliliter (mL) in panel a. Resting, fasting serum total testosterone concentrations are displayed in nanograms (ng) per deciliter (dL) in panel b. Mean values for each time point within each group are listed in bars and error bars represent standard error of the mean. Symbol: ^‡^denotes a significant group by time interaction from repeated measures ANOVA (WPC > PLA and SPC).

Resting, fasted serum estradiol and total testosterone data are presented in Fig. 2. According to Shapiro-Wilks tests of normal residual distributions, PRE serum estradiol residual distributions were non-normally distributed in PLA (p = 0.005) and trended toward non-normality in WPC (p = 0.062), while SPC residuals were normally distributed at PRE (p = 0.191). POST serum estradiol residual distributions were also non-normally distributed in PLA (p = 0.010) and WPC (p < 0.001), while residuals were normally distributed in SPC (p = 0.646). Therefore, data were square root transformed prior to repeated measures ANOVA. Residuals remained non-normally distributed upon square root transformations, so a log_10_ transformation was performed on raw data, resulting in normal distributions for all groups at each level of time, except for PLA estradiol residual distributions at PRE (p = 0.004). No datum from PLA estradiol levels at PRE met criteria for outlier removal so statistical analysis proceeded. In addition to the violation of normality, Levene’s Test of Homogeneity of Variance revealed heterogeneous variance at both PRE and POST time points between groups. Hence, p-values from lower-bound corrections are reported in Fig. 2. No significant effect of time or group × time interaction was observed for serum estradiol (p > 0.05).

Shapiro-Wilks tests revealed that PRE serum testosterone residual distributions were non-normally distributed in SPC (p = 0.002) and trended toward violation in PLA (p = 0.068), while WPC residuals were normally distributed (p = 0.201). POST serum testosterone residual distributions were also non-normally distributed in PLA (p = 0.041) and SPC (p = 0.003), while residuals trended toward violation in WPC (p = 0.075). Therefore, data were square root transformed prior to ANOVA. Upon transformation, all residual distributions were normally distributed. A significant main effect of time (p = 0.039) and group × time interaction was observed for serum testosterone (p = 0.034). Two-tailed independent samples t-tests revealed no significant difference between groups at either time point (p > 0.05). However, a within-group dependent samples t-test revealed a significant increase in serum testosterone within the WPC group (p = 0.049).

### Muscle and Fat Protein Expression

**Figure 3:**
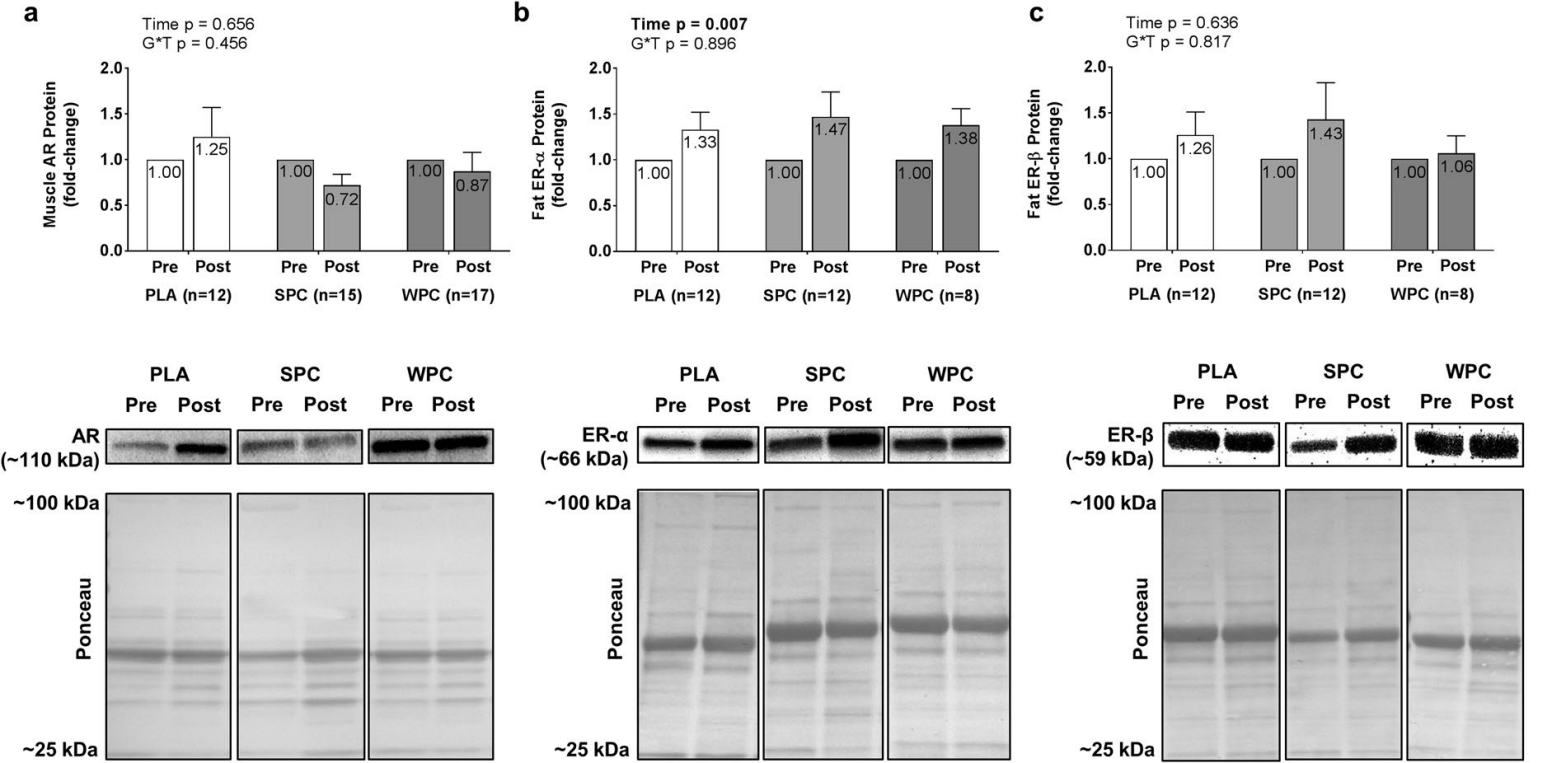
Fold-change values in muscle androgen receptor protein expression (panel a), adipose tissue estrogen receptor-alpha (ERα, panel b), and ERβ (panel c) are displayed. Post protein expression values were divided by pre protein expression values for calculation within each subject. Error bars for fold-change scores represent standard error of the mean. Representative Western blot images were cropped from different parts of the same gels, and representative whole gels are made available in the supplementary information file.

Muscle AR and SQ ER protein expression fold-change data are presented in Fig. 3. Muscle AR protein expression residuals were non-normally distributed, but this was resolved by completing a square root transformation of the raw data. ANOVA revealed no significant effect of time or group × time interaction. A significant main effect of time was identified for SQ ERα protein expression, where expression increased from PRE to POST by 0.390 ± 0.128 fold (+39%, p = 0.004). However, no significant group or group × time interaction was identified. ERβ residual distributions for both PLA and SPC were non-normally distributed (p = 0.003, p < 0.001). Therefore, raw data were square root transformed prior to ANOVA. Upon square root transformation, SPC remained non-normally distributed, so a log_10_ transformation was conducted on the raw data. SPC remained non-normally distributed after log_10_ transformation. Upon further analysis, a subject from SPC expressing a 25-fold change in ERβ was considered an outlier and removed from the data set as this datum was greater than 3 standard deviations from the group mean. Removal of this data point resulted in a normal distribution. Neither a significant main effect of time or group × time interaction was observed for ERβ protein expression with or without this datum included.

### Androgen-responsive mRNA Expression in Muscle and Estrogen-responsive mRNA Expression in Adipose Tissue

**Figure 4:**
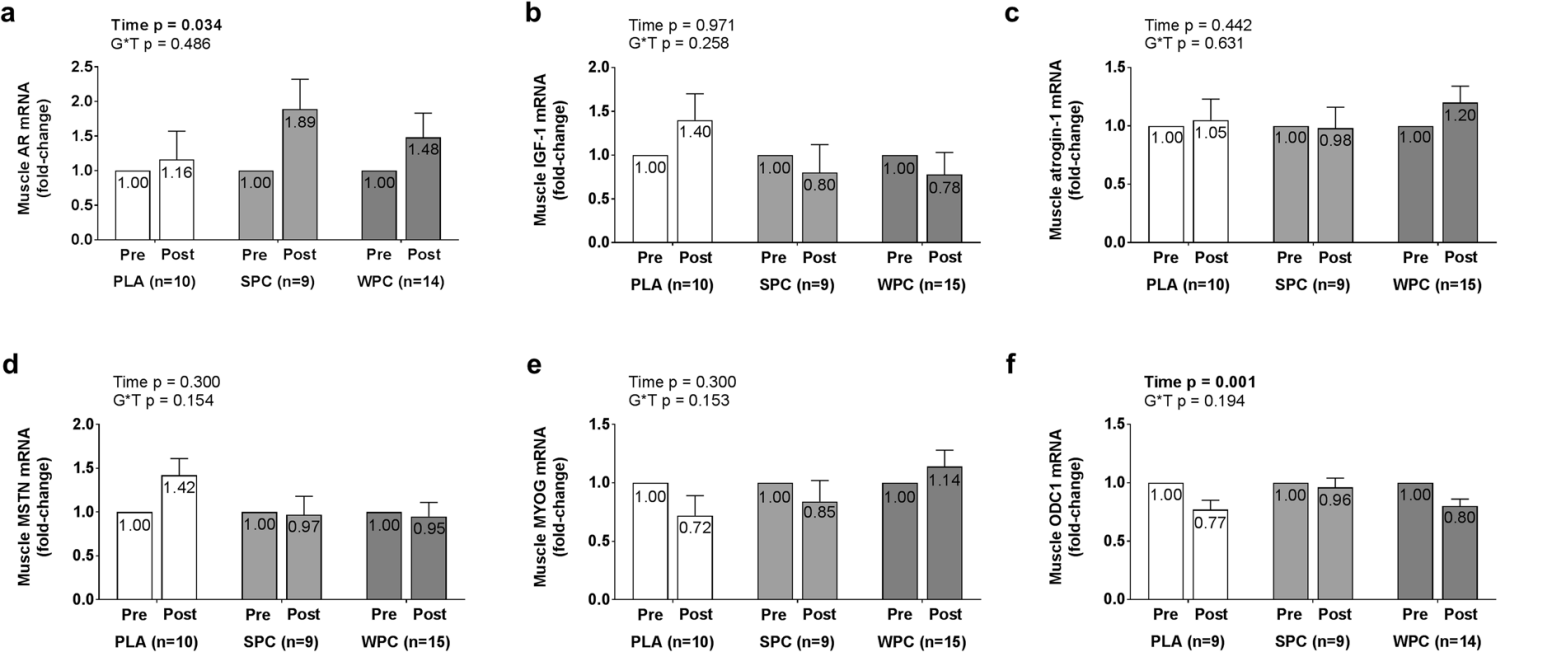
Fold-change values in muscle mRNA expression of androgen-responsive genes are displayed from 2^−ΔΔCT^ method calculations described in the methods based on changes in CT values in the gene of interest relative to the geometric mean of housekeeping genes. Error bars for fold-change scores represent standard error of the mean.

Muscle mRNA expression data are presented in Fig. 4. Similar to SQ protein data analysis, a subject was removed from the analysis expressing mRNA fold-changes greater than three standard deviations from group means in the WPC group due to multiple violations of normality. Upon removal, only ornithine decarboxylase 1 (ODC1) mRNA residuals were non-normally distributed and required transformation. Further analysis revealed two subjects expressing ODC1 mRNA fold-change scores greater than 3 standard deviations from group means, one in PLA and one in WPC, warranting the removal of these subjects from the analysis. Upon square root transformation, ODC1 mRNA were normally distributed. Significant main effects of time were observed for AR and ODC1 mRNA (p = 0.034, p = 0.001). Post-hoc analyses revealed AR mRNA expression significantly increased 0.498 ± 0.225 fold (+49.8%, p = 0.034) and ODC1 mRNA expression significantly decreased 0.249 ± 0.082 fold (−24.9%, p = 0.005).

**Figure 5:**
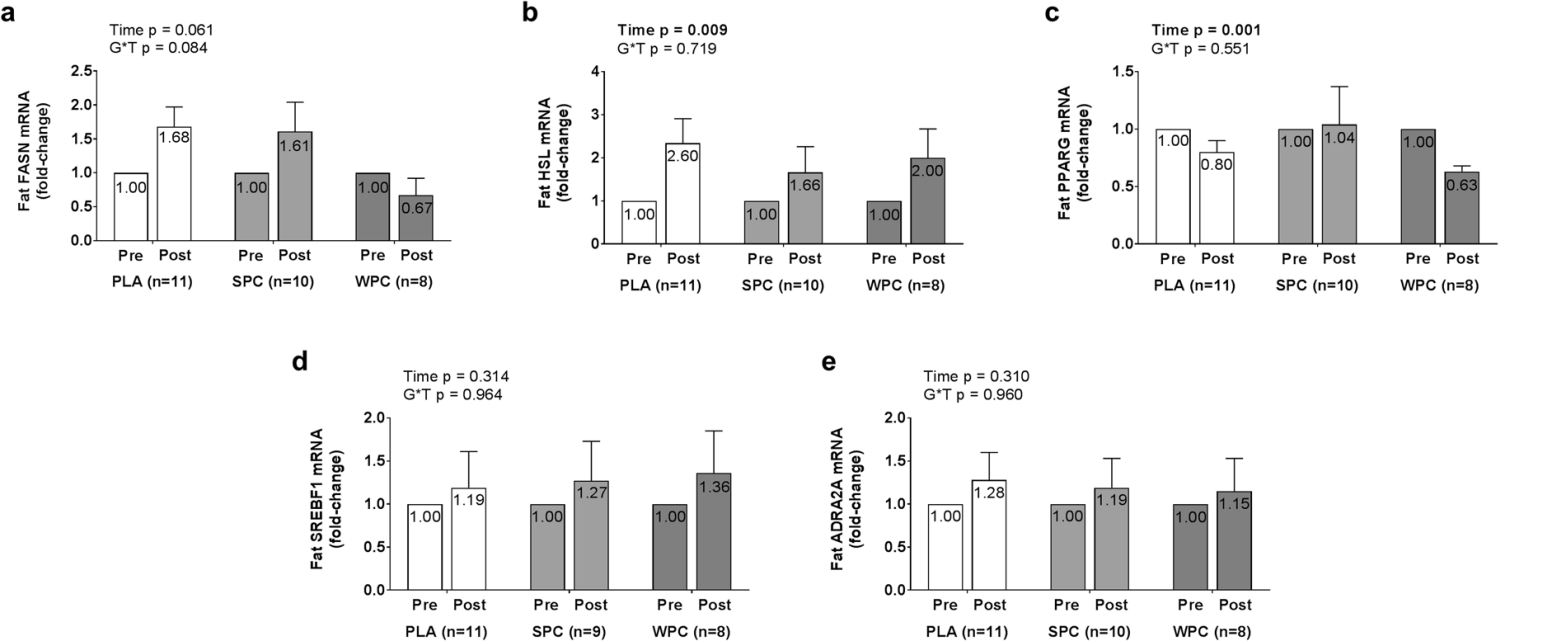
Fold-change values in adipose tissue mRNA expression of estrogen-responsive genes are displayed from 2^−ΔΔCT^ method calculations described in the methods based on changes in CT values in the gene of interest relative to the geometric mean of housekeeping genes. Error bars for fold-change scores represent standard error of the mean.

SQ mRNA expression data are presented in Fig. 5. Adrenoreceptor alpha 2A (ADRA2A), fatty acid synthase (FASN), peroxisome proliferator-activated receptor gamma (PPARG), and sterol regulatory element-binding protein (SREBP1) mRNA residuals were non-normally distributed and were first square root transformed. Log_10_ transformations were performed on these data given square root transformations did not result in normal residual distributions for these mRNA targets. Upon log_10_ transformations, the majority of ADRA2A and PPARG residuals were normally distributed. A single subject was removed from the analysis given fold-change scores for three of the assayed mRNAs in this subject were greater than 3 standard deviations from the mean, resulting in normal residual distributions of FASN and SREBP1. The same criteria for outlier removal described above resulted in the removal of an additional SPC subject for SREBP1 mRNA analysis. Consequently, the majority of group levels for SQ mRNAs were normally distributed. Nevertheless, with or without these data points, no significant group × time interactions were observed for these mRNAs. ANOVA revealed significant effects of time for hormone-sensitive lipase (HSL) and PPARG mRNA expression (p = 0.009, p = 0.001). Post-hoc, two-tailed dependent samples t-tests on PRE and POST arrays revealed HSL mRNA expression significantly increased 1.012 ± 0.344 fold (+101.2%, p = 0.007) while PPARG mRNA expression insignificantly decreased 0.179 ± 0.635 fold (−17.9%, p = 0.139) from PRE to POST.

### Correlations between Dependent Variables and Muscle fCSA or SQ aCSA

**Figure 6:**
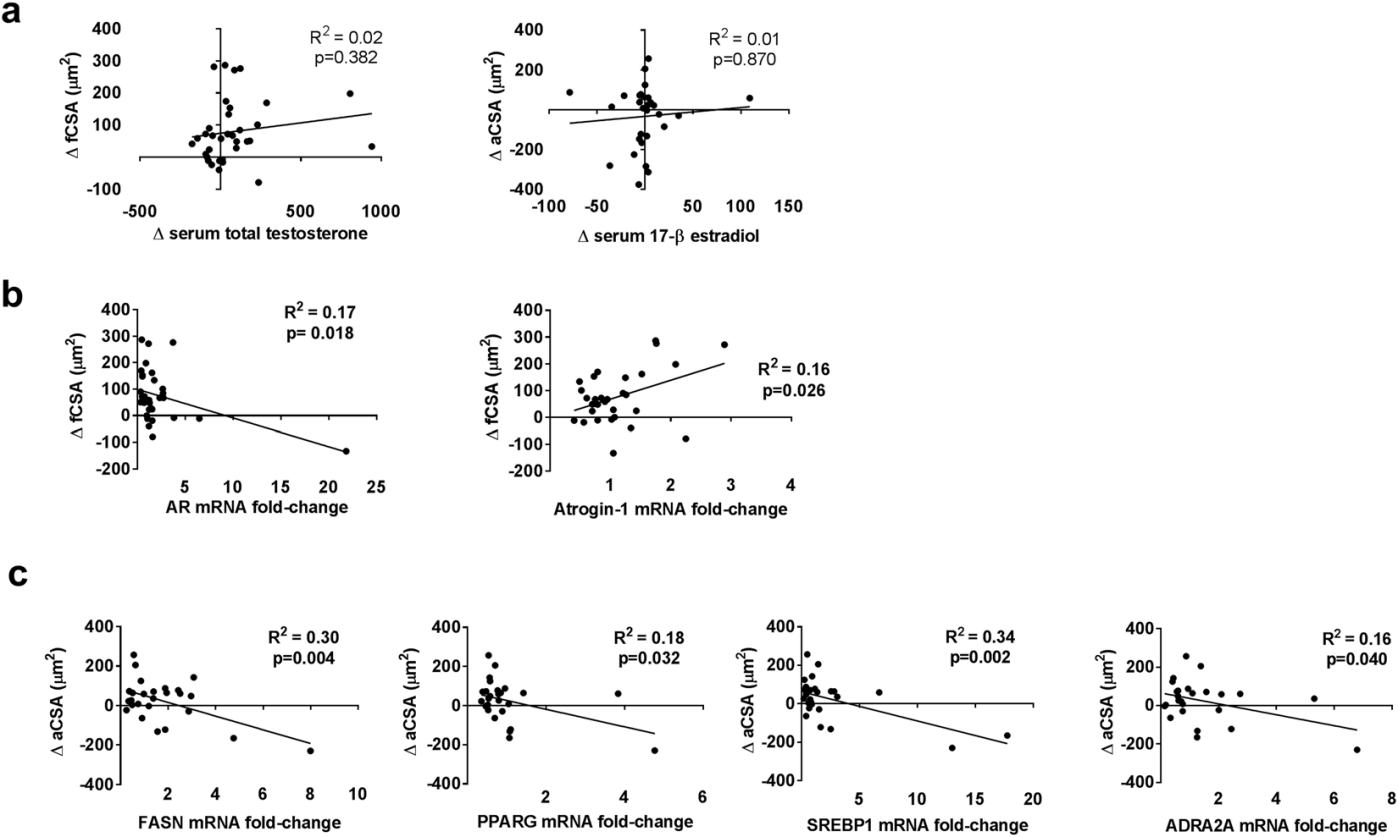
Regression plots demonstrating the relationships between select pre-to-post changes (Δ) in serum hormones and muscle fiber cross sectional area (fCSA) (panel a), muscle androgen-responsive mRNAs and muscle fCSA (panel b), and adipose tissue estrogen-responsive mRNAs and adipocyte cross sectional area (aCSA) (panel c) are displayed. R^2^ is the squared product of Pearson’s r correlation coefficient. A p-value from simple regression analysis is also listed.

Descriptive statistics for muscle fCSA and aCSA are presented in Tables 2 and 3. As previously reported, no significant differences between groups were observed. The relationships between select dependent variables and fCSA or aCSA changes (Δ) are presented in Fig. 6. Briefly, there was no significant relationship between Δ muscle fCSA versus Δ serum total testosterone levels (r = 0.141, p = 0.382, n = 31; Fig. 6a). Additionally, there was no significant relationship between Δ aCSA versus Δ serum 17-β estradiol levels (r = 0.100, p = 0.870, n = 26; Fig. 6a). Significant relationships were observed with Δ muscle fCSA and fold changes in AR mRNA (r = −0.418, p = 0.019, n = 31) and atrogin-1 mRNA (r = 0.402, p = 0.025, n = 31) (Fig. 6b). Moreover, significant relationships existed between changes in Δ aCSA and the following mRNAs: (1) FASN (r = −0.544, p = 0.004, n = 26), (2) PPARG (r = −0.422, p = 0.032, n = 26), (3) SREBP1 (r = −0.579, p = 0.002, n = 26), and ADRA2A (r = −0.405, p = 0.040, n = 26) (Fig. 6c). Finally, no correlations existed between Δ AR protein levels and Δ fCSA values (r = −0.185, p = 0.254, n = 40), Δ ERα protein levels and Δ aCSA values (r = −0.233, p = 0.223, n = 29), or Δ ERβ protein levels and Δ aCSA values (r = 0.147, p = 0.448, n = 29) (*data not shown*).

## Discussion

We sought to determine the effects of SPC, WPC or PLA supplementation on resting, fasted serum estradiol and testosterone, androgen signaling biomarkers in skeletal muscle, and estrogen signaling biomarkers in SQ adipose tissue prior to and following 12 weeks of full-body resistance exercise training. In spite of isoflavone content being substantially higher in the SPC versus the PLA and WPC supplements, we observed the following: 1) serum estradiol concentrations were not significantly altered by supplementation, although serum total testosterone concentrations were increased in the WPC group only, 2) SQ ERα protein expression and HSL mRNA expression significantly increased as a result of training, 3) skeletal muscle AR mRNA increased, but ODC1 mRNA decreased, as a result of training, and 4) ~15% of the variance in muscle fCSA and ~25% in SQ aCSA was associated with alterations in tissue-specific mRNA expression patterns, while changes in serum estradiol and testosterone explained virtually none of the variance in CSA values. Collectively, these data suggest 12 weeks of resistance exercise training, in general, and the combination of resistance exercise training and WPC supplementation, in particular, increases serum testosterone concentration in college-aged men. However, in agreement with data demonstrating that changes in serum testosterone are not predictive of changes in muscle fiber size^11,12^, the observed WPC-induced increases in serum testosterone herein did not correlate with hypertrophic outcomes. Additionally, our observations regarding the effects of resistance exercise training on SQ protein and mRNA expression values are novel to the field of exercise physiology and are discussed in greater detail below.

To our knowledge, we are the first to report changes in SQ protein and mRNA expression alterations following resistance exercise training in college-aged men. SQ ERα protein and HSL mRNA increased in all groups, and this could be indicative of increased lipolysis. Lipolysis via activation of HSL in adipocytes and/or the reduction of lipogenesis has been linked to ER signaling^13–15^. The ERα receptor is expressed in white adipose tissue, and activation of this receptor mediates lipolytic effects through estradiol-binding and resultant molecular signaling at both the level of gene transcription and translation^16,17^. HSL is an enzyme that catalyzes triacylglycerol hydrolysis thereby increasing lipolysis^18^. Considering the aforementioned literature, the concomitant increased expression of ERα protein and HSL mRNA with training is sensible given that SQ aCSA values decreased in all groups by ~300 μm^2^. However, it is notable that SPC and WPC supplementation did not enhance the expression of these biomarkers and, thus, the observed increases in adipose tissue ERα protein and HSL mRNA are likely a result of training and not protein supplementation.

Several studies have noted that chronic resistance exercise training does not affect resting serum testosterone levels in younger, college-aged men^19^. However, discordant data exist regarding the effects of resistance exercise training combined with soy or whey protein supplementation on serum estradiol and testosterone. In contrast to our findings suggesting significant increases in serum testosterone as a result of training with WPC supplementation, Kalman *et al*. reported no significant changes in total serum testosterone after 12 weeks of RT and supplementation with either soy or whey protein fractions in college-aged men^20^. Furthermore, Kalman *et al*. reported significant reductions in serum estradiol after the 12 week supplement and training intervention in all groups, and specifically highlighted statistically significant reductions in the whey blend group whereas we observed no differences in serum estradiol in any group. While it is difficult to explain this discrepancy between studies, a different training methodology was employed. Specifically, both training volume and intensity were increased systematically herein, and our training paradigm consisted of comparatively higher intensities^10^. Hence, while speculative at best, this methodological difference may have contributed to the differential serum estradiol and testosterone profiles observed. Beyond the study by Kalman *et al*., other evidence suggests whey protein consumption before and after a bout of resistance exercise blunts acute elevations in serum testosterone^21,22^. As stated prior, Hulmi *et al*. speculated that this phenomenon could be due to an increased uptake of testosterone into muscle tissue^22^ given: (1) a significant correlation existed between decreases in serum testosterone and increases in AR mRNA expression 1 h after resistance exercise in their study, and (2) evidence suggests that increased testosterone-mediated androgen signaling can increase AR mRNA expression^23,24^. For this reason, we performed an exploratory analysis of intramuscular testosterone concentrations in a subset of muscle samples using liquid-liquid extraction and LC-MS/MS methods similar to methods described by Singh^25^. Interestingly, intramuscular testosterone concentrations were too low to precisely calculate (<instrument detection limit (IDL): 0.1 ng/mL). Furthermore, AR mRNA expression increased in all groups following 12 weeks of training, and exhibited no significant between-group differences. Hence, it seems unlikely that chronic differences in the muscular uptake of testosterone occurred between groups. Notwithstanding, the observation that total serum testosterone was higher with WPC supplementation following 12 weeks of resistance exercise training is a novel finding. While speculative, this observation could be related to the enrichment of corticosteroid-binding proteins (CBPs) in the WPC supplement and the consequential presence thereof in serum following 12 weeks of twice-per-day consumption by subjects in the WPC group^26,27^. CBPs have been shown to be present in whey protein^27^, and CBPs in serum can bind circulating testosterone and thereby increase the measurement of total testosterone^26^. However, this speculation is limited given that CBPs contained within whey protein are likely hydrolyzed in the digestive tract. What seems more probable are the potential effects that WPC supplementation may have had on increasing serum IGF-1 levels. While not measured in the current study, others have reported that whey protein supplementation increases serum IGF-1 levels^28^, and IGF-1 has been shown to stimulate testosterone biosynthesis^29^. Despite these potential mechanisms which may explain why WPC supplementation increased resting, fasting levels of testosterone, serum examination of IGF-1 was not feasible due to a lack of remaining serum samples. Hence, future research should examine the potential relationship that exists between whey protein supplementation and testosterone biosynthesis.

While muscle AR mRNA expression increased with training regardless of supplementation, it is notable that a moderate PRE to POST increase was only observed in the WPC group (Cohen’s d = 0.59; Table 11 in supplementary data). As previously mentioned, androgens (e.g., testosterone) increase expression of their receptors^23,24^, and the increase in serum total testosterone over time corroborates an increase in skeletal muscle AR mRNA. Hence, this may provide limited evidence that increases in serum testosterone via WPC supplementation does modestly affect this androgen signaling biomarker. It is interesting, however, that AR protein levels were not altered in spite of training-induced increases in mRNA levels. While this finding is difficult to reconcile, others have noted in cell culture studies that mRNA and protein levels poorly correlate, and this phenomena is primarily due to the variable half-life of proteins^30^. In this regard, a cell culture-based study has determined that the AR protein has a short half-life of ~3-6 hours^31^. Thus, while muscle AR mRNA was elevated with resistance exercise training herein, AR protein levels may have not changed due to a relatively higher turnover rate of this protein. We also observed that muscle ODC1 mRNA expression decreased over time regardless of supplementation. ODC1 is a rate-limiting enzyme in polyamine (e.g., putrescine, spermidine, spermine) synthesis^32^. Turchanowa *et al*. reported significant increases in ODC1 and polyamine concentrations in rodent skeletal muscle during recovery from resistance exercise^33^. Additionally, Cepero *et al*. reported significantly increased polyamine concentrations in hypertrophied rodent skeletal muscle after 3 months of aerobic exercise^34^. Although a detailed understanding of ODC1 signaling in human skeletal muscle is premature, Lee *et al*. have recently shown upregulation of ODC1 mRNA by AR signaling and a regulatory role in proliferation in cultured human skeletal muscle myoblasts^32^. To our knowledge, no comparative data of skeletal muscle ODC1 mRNA expression after chronic resistance exercise training exists. However, a potential explanation of our ODC1 mRNA findings could be related to a rather pulsatile nature of ODC1 mRNA expression corresponding to the acute increase in androgen signaling after a bout of resistance exercise. That is, ODC1 mRNA expression could be acutely increased after a bout of exercise coincident to acute increases in androgen signaling, followed by a transient down-regulation captured by the muscle sample collected at POST. Notwithstanding, more data are needed in order to determine the physiological role that ODC1 gene expression plays in skeletal muscle physiology.

One final observation noted herein is that WPC supplementation had the largest effect on increasing type II muscle fCSA values (Cohen’s d = 1.30), whereas SPC had the largest effect on increasing this metric in type I fibers (Cohen’s d = 0.84) (Tables 26 and 27 in supplementary data). Indeed, this is a relatively novel finding and it could implicate differential effects of each protein source on promoting fiber type-specific hypertrophy. Regarding the differential physiological effects that each protein source produces, van Vliet *et al*. have suggested that SPC supplementation has a greater stimulatory effect on splanchnic protein synthesis rates, whereas milk-based proteins better stimulate muscle protein synthesis (MPS) rates^35^. However, research in the area of fiber type-specific MPS or anabolic responses to plant- versus milk-based protein supplements have not been explored, and our data warrant future research in this area.

Certain limitations to the current study exist. Notably, only select sex hormones were assayed due to resource constraints. In this regard, replicating this current research design with more blood sampling time points (e.g., weekly) whereby markers of testosterone biosynthesis (e.g., DHEA, androstenedione), bioavailable testosterone (e.g., SHBG, total testosterone, free testosterone) as well as different estrogens (e.g., estrone, estriol) may add insightful data to these current findings. Additionally, acute post-exercise WPC versus SPC supplementation studies whereby blood and biopsies are obtained hours following a single exercise bout will better determine if androgenic or estrogenic signaling is impacted by different protein supplements. In spite of these limitations, we are the first to present evidence suggesting biomarkers of lipolysis increase with longer-term resistance exercise training in college-aged men, albeit WPC or SPC supplementation did not augment these effects. Furthermore, we provide unique preliminary evidence that WPC may increase fasted, resting serum testosterone while SPC supplementation combined with training does not appreciably affect biomarkers indicative of androgenic or estrogenic signaling in muscle or adipose tissue, respectively. Finally, the differential effects that WPC versus SPC has on fiber type-specific adaptations to resistance exercise training should be further investigated.

## Materials and Methods

### Ethical Approval and Participant Screening

Prior to initiating this study, the protocol was reviewed and approved by the Auburn University

Institutional Review Board (IRB), and was conducted in accordance with the Declaration of Helsinki (approved protocol #: 15–320 MR 1508; IRB contact: irbadmin@auburn.edu). Additionally, this trial was registered at ClinicalTrials.gov (Identifier: NCT03501628, date registered: April 18, 2018). Apparently healthy, college-aged males (i.e., 19–23 years old) naïve to resistance exercise training were recruited locally for this experiment. Participants provided both verbal and written informed consent to participate in the study and completed a medical history form before study initiation. Screening forms were administered to ensure participants had not engaged in regular resistance exercise for the previous six months and that participants were not consuming a high-protein diet (i.e., >2 g/kg/d). Additionally, participants verified no use of anabolic steroids, supplemental protein, creatine monohydrate, or prohormones. Baseline testing occurred one week following screening.

### Experiment Overview

**Figure 7:**
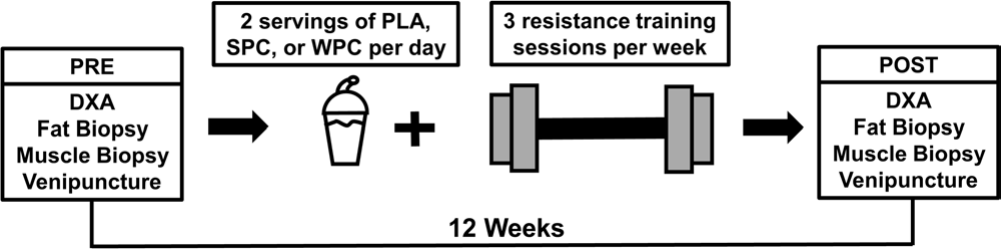
An overview of the experiment is provided. Subjects completed PRE testing consisting of dual-energy x-ray absorptiometry tests of body composition, a muscle biopsy of the vastus lateralis, a fat biopsy of subcutaneous fat in the gluteal region, and venipuncture. Participants then completed 12 weeks of resistance exercise training and consumed two servings of either PLA, SPC, or WPC per day. POST testing consisted of the same assessments as PRE, and both assessments were under a 4-hour fast around the same time of day (±2 hours). Additionally, PRE and POST tests were 72 hours following the last resistance exercise training bout along, and WPC/SPC/PLA supplementation occurred up to the day prior to POST assessments.

Readers are directed to our previous manuscript for a more detailed breakdown of the parent study design, procedures, and additional supplement information^10^. Figure 7 provides a visual representation of the timeline of the investigation, and the points at which biological samples analyzed herein were collected. Briefly, baseline (PRE) blood, body composition, and muscle biopsy samples were collected 12 weeks prior to the collection of post-intervention samples (POST). Between these two time points, subjects consumed either PLA, SPC, or WPC twice per day, while both subjects and researchers were blinded to subject grouping. Prior to PRE blood and tissue sample collections, subjects were instructed to refrain from rigorous physical activity for 4–5 days. Subjects were also instructed to report to the laboratory well-hydrated and at least 4 hours fasted on the day of testing. Subject hydration status was confirmed via urine specific gravity and DXA measurements were performed upon hydration confirmation. After DXA scans were complete, venipuncture and biopsies of the vastus lateralis muscle and subcutaneous fat in the gluteal region were performed. During the intervention, subjects completed resistance exercise three times per week, as previously described^10^, and were closely monitored by research staff for appropriate technical execution of the exercises. Resistance exercises included the barbell back squat, barbell bench press, trap-bar deadlift, and barbell bent row. Load magnitude for each exercise was progressively increased throughout the study, so long as subjects maintained proper lifting technique. The experimental procedures employed to process and analyze blood serum, body composition data, fat tissue, and muscle tissue samples are described in detail below. Additionally, LC-MS/MS methods for the measurement of supplement phytoestrogen contents are described below.

### Supplement Phytoestrogen Analysis

We sought to accurately measure isoflavone contents of each supplement given their proposed role in androgen and estrogen signaling. For this analysis we randomly selected five separate packets of each supplement and used LC-MS/MS to measure the total daidzein and genistein concentrations in each packet. 2–3 mg of powder from each supplement was weighed and added to a 5 mL glass tube. Samples were sonicated in 1 mL methanol for 30 min to extract phytoestrogens. 500 μL of methanol extract (and standards) were collected and added to a 1.5 mL centrifuge tube. An aliquot of 100 µL of internal standard was added to sample tubes. Samples were centrifuged at 14,000 *g* for 15 min. 200 µL of supernatant was collected and transferred into an Agilent injection vial for LC-MS/MS measurement. The LC-MS/MS measurement of genistein, daidzein, and their internal standard (Quercetin), was performed on an Agilent 1290 UHPLC system coupled Agilent 6460 Triple Quad Mass Spectrometer. The mobile phase consists of 10 mM ammonium format and methanol. The samples were separated on ACQUITY UPLC^®^ HSS T3 column (2.1 × 100 mm, 1.8 μm) using a gradient from 60% of methanol to 80% over 1.5 min, then ran for 0.5 min at 80% of methanol. The samples were introduced into the mass spectrometer with a flow rate of 0.4 mL/min using an Agilent Jet Stream Electrospray Ionization (AJ ESI) source. Nitrogen gas was used as the dry (10 L/min at 350 °C), nebulizer (45 psi), and collision gas. Mass spectra were acquired in negative-ion mode and capillary voltage was set at −3500 V. Mass transitions were monitored using multiple-reaction monitoring (MRM). The transitions for each analyte are listed in Table 4.

**Table 4:** LC-MS/MS Parameters.

Compound	Type of Transition	Mass Transition (MRM)	Fragmentor (V)	Collision Energy (V)
Genistein	Quantifier ion	269.0–133.0	130	29
	Qualifier ion	269.0–224.0	130	22
Daidzein	Quantifier ion	253.0–222.9	130	31
	Qualifier ion	253.0–207.9	130	29
Quercetin	Quantifier ion	301.0–150.9	110	14
	Qualifier ion	301.0–107.0	110	25

### Serum Hormone Analyses

Fasting blood samples were aseptically collected into 5 mL serum separator tubes (BD Vacutainer, Franklin Lakes, NJ, USA) from the antecubital vein before and after 12 weeks of supplementation and training during the same time of day (±2 hours). Notably, SPC, WPC or PLA supplementation occurred up until the day prior to blood and tissue extraction. After collection in serum tubes, blood was centrifuged at 4,000 g at 4 °C for 5 min. Serum was then pipetted into cryotubes and placed in a −80 °C freezer for batch-processing. 17β estradiol and total testosterone concentrations in serum were measured using commercially available enzyme linked immunosorbent assay kits (ELISAs, ALPCO Diagnostics, Salem, NH, USA).

### Fat and Muscle Biopsies

Methodology for fat and muscle biopsies along with tissue processing procedures in our laboratory have been described in detail elsewhere^10^, however, a brief description follows. After the extraction of muscle tissue from the right *vastus lateralis* approximately midway between the patella and iliac crest using a 5 gauge Bergstöm needle with suction and sterile laboratory procedures, a fat tissue biopsy was also completed. A 20–40 mg portion of muscle tissue was placed in a cryomold and suspended in optimal cutting temperature media (OCT; Electron Microscopy Sciences, Hatfield, PA, USA) for histochemical analysis to measure the cross sectional area of muscle fibers. The approximate 100 mg of remaining muscle tissue was placed in foil and snap frozen for protein and RNA isolation batch processing. Approximately 100 mg of SQ fat was extracted from the gluteal aspect of the left hip. 20–40 mg of SQ fat was placed in 10% formalin and preserved for hematoxylin and eosin staining and histological analysis for adipocyte cross sectional area analysis. Approximately 50–75 mg of fat tissue was placed in foil and snap frozen in liquid nitrogen for protein and RNA isolation procedures.

### Fat and Muscle mRNA Expression Analyses

At the conclusion of the study, ~25 mg of fat and muscle tissue placed in foil was removed and homogenized in 500 μL of Ribozol (Ameresco) depending on the amount of tissue available and stored at −80 °C for batch processing. Upon removal for batch processing, RNA isolation occurred according to manufacturer’s instructions. RNA concentrations were assessed using a NanoDrop Lite (Thermo Scientific, Waltham, MA, USA) prior to cDNA synthesis. cDNA was synthesized via reverse transcription from 1,000 ng of total RNA for real time PCR analyses employing a commercial cDNA synthesis kit (Quanta Biosciences, Gaithersburg, MD, USA). SYBR green-based methods with gene-specific primers designed using an online primer designer tool (Primer3Plus, Cambridge, MA, USA) were subsequently performed. Genes are denoted in Table 5. The comparative threshold cycle (CT) method (2^−ΔΔCT^) was used to calculate relative gene expression^36^. Melt curve analyses were performed on the first PCR plate for each gene to ensure that one PCR product was amplified per reaction.

**Table 5:** Primer Sequences for RT-PCR.

Gene	Forward primer (5′ → 3′)	Reverse primer (5′ → 3′)
*AR Responsive Genes*		
AR	ATCATCACAGCCTGTTGAACT	CAATCCCGACCCTTCCCAG
Atrogin-1	ATGTGCGTGTATCGGATGG	AAGGCAGGTCAGTGAAGC
IGF-1	GTGGATGAGTGCTGCTTC	GGTTCTGGGTCTTCCTTC
Myostatin	GACCAGGAGAAGATGGGCTGAATCCGTT	CTCATCACAGTCAAGACCAAAATCCCTT
Myogenin	GCCAGACTATCCCCTTCCTC	GAGGCCGCGTTATGATAAAA
ODC-1	GACGGGCTCTGATGGTATGT	TCCATAGACGCCATCATTCA
*ER Responsive Genes*		
α-adr	ACTGGACTACAAGGGCATGG	ACATCAAAACCAAGGCCAAG
FASN	GTCTTGAACTCCTTGGCGGA	GAGCGGGTGGTTCTGAGAAA
HSL	GTCCTCGTCAGGCTCATCTC	CTCTTGAGGTAGGGCTCGTG
PPAR-γ	GCCCAGGTTTGCTGAATGTG	TTGGCAAACAGCTGTGAGGA
SREBP1	TTCGCTTTCTGCAACACAGC	AGTGTGTCCTCCACCTCAGT
*Housekeeping Genes*		
28 s	GCGTTGGATTGTTCACCCAC	ACCTGTCTCACGACGGTCTA
cyclophilin	CGATGTCTCAGAGCACGAAA	CCCACCTGTTTCTTCGACAT
Fbl	CCCACACCTTCCTGCGTAAT	GCTGAGGCTGTGGAGTCAAT

All primers were designed using PrimerPlus3 (Cambridge, MA, USA) and BLASTed against other potential mRNA targets using the online NCBI Nucleotide database (Bethesda, MD).

### Fat and Muscle Protein Expression Analyses

Approximately 75 mg of fat and muscle tissue were placed in cryotubes containing 250–500 μL of cell lysis buffer, depending on the amount of tissue remaining after the previously mentioned tissue allocations. Insoluble proteins were removed by centrifugation at 500 *g* for 5 min at 4 °C and supernatants containing homogenates were stored at −80 °C. Tissue homogenates were batch-assayed for total protein content using a BCA Protein Assay Kit (Thermo Scientific, Waltham, MA, USA). After calculation of sample protein concentrations, homogenates were prepared for Western blotting using 4x Laemmli buffer. Fat tissue lysates were prepared at concentrations of 750 ng/μL and muscle tissue lysates were prepared at 1 μg/μL concentrations. For immunoblotting, 25 μL of samples were loaded onto 4–15% SDS-polyacrylamide gels (BioRad, Hercules, CA, USA) and subjected to electrophoresis (180 V for 60 min) using 1x SDS-PAGE running buffer (Ameresco). Proteins were subsequently transferred to polyvinyl difluoride membranes (BioRad), Ponceau stained, and imaged using a gel documentation system (UVP, Upland, CA, USA) to ensure equal protein loading between lanes. Additionally, Ponceau images served to provide a proxy of total sample loaded and protein expression values were normalized on a per-lane basis to account for any differences in sample loading. After this, membranes were blocked for 1 hour at room temperature with 5% nonfat milk powder. For muscle samples, membranes were incubated overnight with rabbit anti-human AR (1:1,000; Thermo Fisher Scientific) primary antibodies at 4 °C in 5% bovine serum albumin (BSA). For fat samples, membranes were incubated overnight with mouse ER-α (1:500; Hybridoma Bank) and separately with mouse ER-β (1:500; Hybridoma Bank) at 4 °C in 5% bovine serum albumin (BSA). The following day, membranes were incubated with horseradish peroxidase-conjugated goat anti-rabbit IgG (1:2000; Cell Signaling) and anti-mouse IgG (1:2000; Cell Signaling) secondary antibodies at room temperature for 1 h prior to membrane development. Membrane development was performed using an enhanced chemiluminescent reagent (Luminata Forte HRP substrate; Millipore, Billerica, MA, USA), and band densitometry was performed through the use of a gel documentation system and associated densitometry software (UVP). Densitometry values for all protein targets were normalized to Ponceau densities. POST protein expression values were subsequently normalized to PRE values in order to obtain fold-change values where: POST/PRE = fold-change.

### Fat and Muscle Immunohistochemical Analyses

SQ aCSA and muscle fCSA analysis was performed as previously published by our laboratory^10^. SQ fat samples were removed from formalin and washed in cold running tap water, embedded, and stored in 70% alcohol. Dehydration was accomplished by gradually increasing percentages of ethyl alcohol to replace the water content in the tissue. Hemo-De was used thereafter to clear the tissue from the ethyl alcohol and allow infiltration with paraffin. The paraffin tissue blocks were sectioned into 6 µm slices and placed onto glass microscope slides. Paraffin was removed with xylene, mounted sections were stained with hematoxylin and eosin, and sample sections were enclosed with a coverslip and mounting media. Two 10x objective digital images per sample were obtained using bright-field imaging (Nikon Instruments), and CSAs were obtained from at least 100 adipocytes per image using ImageJ (National Institutes of Health, Bethesda, MD, USA). For muscle samples, sections from OCT‐preserved samples were cut at a thickness of 20 μm using a cryotome (Leica Biosystems; Buffalo Grove, IL, USA) and were adhered to positively-charged histology slides. Sections of muscle tissue were dried at room temperature for 30 min and incubated in a phosphate‐buffered saline (PBS) solution containing 0.5% Triton X‐100, and blocked with Pierce Super Blocker (Thermo Fisher Scientific). Sections were then rinsed in PBS and incubated with primary antibodies for 1 h. The primary antibodies used for muscle fiber-typing were rabbit anti-dystrophin IgG (Thermo Fisher Scientific; 10 µL antibody per 1 mL of blocking solution) and mouse anti-myosin II IgG (catalog #: SC71; Hybridoma Bank; 100 µL per 1 mL of blocking solution). Sections were then washed for 5 min in PBS and incubated in the dark for 60 min with a secondary antibody solution containing Texas Red-conjugated anti-rabbit IgG (Vector Laboratories, Burlingame, CA, USA), and Alexa Fluor 488-conjugated anti-mouse IgG (Thermo Fisher Scientific) (10 µL of all secondary antibodies per 1 mL of blocking solution). Sections were then washed for 5 min in PBS, air-dried, and were mounted with fluorescent media containing 4,6-diamidino-2-phenylindole (DAPI; Vector Laboratories). Following mounting, slides were stored in the dark at 4 °C until immunofluorescent images were obtained. Digital images were captured using a fluorescence microscope (Nikon Instruments, Melville, NY, USA) and 20x objective. Approximate exposure times of 600 ms for red and green imaging were used while only 30 ms exposure occurred for blue imaging. For fiber typing, our staining method allowed the identification of cell membranes (detected by the Texas Red filter), type II fiber green cell bodies (detected by the FITC filter), type I fiber black cell bodies (unlabeled), and myonuclei (detected by the DAPI filter). Measurements of muscle fiber cross sectional area (CSA) were performed using the open-sourced software CellProfiler^TM^ per modified methods previously described^37^, whereby the number of pixels counted within the border of each muscle fiber were converted to a total area in microns-squared (µm^2^). Based on data from Mackey *et al*.^38^ and Murach *et al*.^39^, at least 50 fibers per specimen were quantified in order to obtain accurate CSAs.

### Statistical Analyses

Statistical tests were performed in RStudio (Version 1.0.143) and SPSS (Version 23). The average sample size for each dependent variable was 33 subjects, while specific sample sizes for each analysis are reported in figures or text. Group (3 levels [PLA, SPC, WPC]) and time (2 levels [PRE, POST]) served as independent variables in this analysis. Measurement of each dependent variable occurred at each time point. *A-priori* power analyses in RStudio using general linear model parameters in the “pwr” package (Version 1.2–1) revealed 79% power (power = 1 − β) for the discovery of a medium size effect, and 98% power for the discovery of a large effect when analyses included 33 subjects with 2 observations (i.e., 66 total observations). Supplementary Tables provide descriptive statistics for each dependent variable with no outlier removal, mean differences, Cohen’s *d* effect sizes, and 95% confidence intervals. Data are not restated in results when included in figures. Data are reported as mean ± standard error of the mean, and are based on any data removal described below. The alpha level of significance was set a-priori to: p ≤ 0.050. Statistical assumptions tests were completed prior to statistical analysis consisting of: a) Shapiro-Wilks tests of residual distributions for normality, and b) Levene’s test of homogeneity of variance, given that a repeated measures analysis of variance (ANOVA) was performed for the provision of p-values. Violation of these assumptions are reported and appropriate data transformations (i.e., square root or log_10_ transformations) were completed when residuals were not normally distributed prior to ANOVA for the avoidance of type 1 or type 2 errors. Data transformation and data removal were avoided with intention to report raw data and associated inferential statistics. For this reason, if the majority of levels of group (2 of 3 groups) at each level of time were normally distributed, ANOVA proceeded. However, if datum met both of the following two criteria, datum were removed to best reflect group responses and more confidently infer effects: 1) Shapiro-Wilks tests revealed non-normal distributions for the data array and, 2) the datum was ≥3 standard deviations from the group mean. Although rare, cases of data removal were reported. For significant group × time interactions, two-tailed independent samples t-tests were performed as a post-hoc test. For significant time *F-*statistics identified from ANOVA, a 2-tailed dependent samples t-test was performed comparing PRE to POST data arrays. Lastly, to examine the relationships between dependent variables and changes in SQ aCSA and muscle fCSA, independent of group, simple regression analyses were performed. No datum were removed for this analysis given the intent to examine overall relationships rather than make between-group comparisons. Specifically, changes in SQ aCSA were regressed by: (1) serum estradiol, (2) serum testosterone, (3) fat ER protein expression, and (4) fat mRNA expression. Changes in muscle fCSA were separately regressed by: (1) serum estradiol, (2) serum testosterone, (3) muscle AR protein expression, and (4) muscle mRNA expression.

## Electronic supplementary material

Project summary

Supplementary figures and tables
