# Author Correction: Soy protein supplementation is not androgenic or estrogenic in college-aged men when combined with resistance exercise training

**DOI:** 10.1038/s41598-018-30574-8

**Published:** 2018-08-10

**Authors:** Cody T. Haun, C. Brooks Mobley, Christopher G. Vann, Matthew A. Romero, Paul A. Roberson, Petey W. Mumford, Wesley C. Kephart, James C. Healy, Romil K. Patel, Shelby C. Osburn, Darren T. Beck, Robert D. Arnold, Ben Nie, Christopher M. Lockwood, Michael D. Roberts

**Affiliations:** 1 Molecular and Applied Sciences Laboratory, School of Kinesiology, Auburn University, Auburn, AL USA; 2 Department of Cell Biology and Physiology, Edward Via College of Osteopathic Medicine – Auburn Campus, Auburn, AL USA; 3 Lockwood, LLC, Draper, UT 84020 USA; 4 Department of Drug Discovery & Development, Harrison School of Pharmacy, Auburn University Pharmaceutical Research Building, Auburn, AL USA

Correction to: *Scientific Reports* 10.1038/s41598-018-29591-4, published online 24 July 2018

The original version of this Article contained errors.

In the Abstract,

“Limited also evidence suggests that whey protein supplementation may increase androgenic signaling.”

now reads:

“Limited evidence suggests that whey protein supplementation may increase androgenic signaling.”

In the Results,

“No datum from PLA estradiol levels at PRE met criteria for outlier removal and given that all other levels were normally distributed.”

now reads:

“No datum from PLA estradiol levels at PRE met criteria for outlier removal so statistical analysis proceeded.”

In the Discussion,

“Notwithstanding, more data are needed in order to determine the physiological role that ODC1 gene expression has on skeletal muscle physiology.”

now reads:

“Notwithstanding, more data are needed in order to determine the physiological role that ODC1 gene expression plays in skeletal muscle physiology.”

Finally, in the Discussion,

“Indeed, this is a relatively novel finding and it could implicate the differential role that each protein source has on promoting fiber type-specific hypertrophy.”

now reads:

“Indeed, this is a relatively novel finding and it could implicate differential effects of each protein source on promoting fiber type-specific hypertrophy.”

These errors have now been corrected in the PDF and HTML versions of the Article.

