# Supplementary material for: Soy protein supplementation is not androgenic or estrogenic in college-aged men when combined with resistance exercise training
Source: Sci Rep. 2018 Jul 24;8:11151. doi: 10.1038/s41598-018-29591-4 (PMC6057888; doi:10.1038/s41598-018-29591-4)
Supplement: Supplementary file 1 — Project summary [file 41598_2018_29591_MOESM1_ESM.pdf]

**WHO recommended format for a Research Protocol per the request of the editor regarding manuscript SREP-17-46851A titled "*Soy protein supplementation is not androgenic or estrogenic in college-aged men when combined with resistance exercise training*"**

## Project summary

Dietary protein supplementation and/or amino acid supplementation is a widespread practice in individuals that regularly engage in resistance exercise training (i.e., weightlifting). This is for good reason, as the scientific literature has determined that these supplements are: a) extraordinarily safe, and b) effective at promoting increases in muscle mass beyond increases that would naturally occur in an un-supplemented state. There are an array of different protein and amino acid supplements that are currently sold over the counter. These include, but are not limited to: 1) whey protein (concentrate, isolate, or hydrolysate), 2) soy protein (concentrate or isolate), 3) egg protein (concentrate or hydrolysate), 4) casein protein (concentrate or hydrolysate), and 5) branched chain amino acids.

While 'meta-data' (or compiled data from various studies) exists differentially comparing the anabolic effects of various protein supplements, sparse evidence exists comparing the differential anabolic effects of amino acids or protein supplements in one study. Therefore, the purpose of this study was to compare the effects of L-leucine+maltodextrin supplementation, whey protein concentrate supplementation, hydrolyzed whey protein supplementation, or soy protein supplementation on changes in lean tissue mass (assessed by dual x-ray absorptiometry). Screened participants will be untrained, unsupplemented males between the ages of 19-35. Training and supplementation will occur over a 12-week period and is described in greater detail below.

## General information

*Protocol title*: The Effects of Different Protein and Amino Acid Supplements on Muscle Hypertrophy after 12 Weeks of Resistance Exercise in Untrained Men

*Name/Address of Sponsor/funder*: gift funds were graciously provided by Hilmar Ingredients (Hilmar, CA, USA) and Bionutritional Research Group (Irvine, CA, USA).

*Name and title of the investigator(s) who is (are) responsible for conducting the research*: Michael D. Roberts, PhD; Associate Professor, School of Kinesiology, Auburn University, Auburn, AL, USA

*Name(s) and address(es) of the clinical laboratory(ies) and other medical and/or technical department(s) and/or institutions involved in the research*: Molecular and Applied Sciences Laboratory, 301 Wire Road, School of Kinesiology, Auburn University, Auburn, AL 36849, USA

**Rationale & background information**

There is widespread evidence suggesting protein supplementation enhances resistance training adaptations. For instance, Cribb et al. [1] reported that resistance trained participants who consumed 45 g/d of whey protein isolate following 10 weeks of resistance training achieved a 5 kg increase in lean body mass (LBM) which was 4.2 kg greater than a casein-supplemented group. Burke et al. [2,3] reported that whey protein supplementation promoted 2.3-2.5 kg increases in LBM with 6 weeks of resistance training which was ~1.4 kg greater than the effects observed in these studies' maltodextrin placebo (PLA) groups. Notably, a soy protein group was also examined in one of the two aforementioned studies [3], and this group experienced a 1.7 kg increase in LBM which was not statistically greater than the PLA group. Hulmi et al. [4] reported that 10.5 weeks of resistance training and whey protein supplementation elicited a 2.5 kg increase in LBM which was 0.5 kg greater than the non-energetic PLA group. Furthermore, Volek et al. [5] reported that 9 months of resistance training combined with whey or soy protein supplementation resulted in 3.6 and 2.6 kg increases in LBM, respectively. While the aforementioned studies did not determine the mechanisms responsible for the reported phenotypic changes in skeletal muscle mass, others have postulated that protein supplementation reduces fast to slow isoform shifts [6] and promotes myogenic responses to resistance training [7]. A recent review by Morton et al. [8] provides additional studies reporting that protein supplementation in conjunction with resistance training enhances indices of skeletal muscle anabolism, although other studies have reported that protein supplementation has no added benefit when performed in conjunction with 8-12 weeks of resistance training [9,10].

Contrary to much of the positive data supporting whey protein supplementation, the data appears to be less favorable regarding the effects of amino acid-only supplementation in enhancing resistance training induced increases in LBM. For instance, Bird et al. [11] reported that essential amino acid (EAA) supplementation (6 g/d) led to ~3 kg increase in LBM following 12 weeks of resistance training twice a week in younger untrained males, albeit these increases were not significantly different from a PLA group. Vieillevoye et al. [12] reported similar findings in younger untrained males whereby 15 g/d of EAA supplementation during 12 weeks of resistance training did not increase LBM compared to a sucrose PLA group. Additionally, Aguilar et al. [13] recently reported that younger male subjects who supplemented with L-leucine (LEU; 3 g/d) during 8 weeks of resistance training experienced no additional increase in quadriceps muscle size increases when compared to subjects consuming a cornstarch PLA.

In spite of the aforementioned literature suggesting whey or soy protein supplementation may be more effective than EAA or LEU in promoting additional increases in LBM with resistance training, a prevailing hypothesis is that the LEU content of a given dietary protein determines the efficacy of how that protein potentiates muscle growth. However, this hypothesis is based on acute human, animal, or cell culture-based studies reporting that LEU or whey protein (which contains 8-11% LEU) optimally stimulates muscle protein synthesis [14-17]. To this end, there is no evidence which has directly compared the anabolic effects of LEU supplementation versus supplementation with other dietary protein sources that contain high levels of LEU (e.g., whey or soy).

**Study goals and objectives**

The purpose of this study was to examine if supplementation with LEU, whey protein concentrate (WPC), whey protein hydrolysate (WPH) or soy protein concentrate (SPC) enhances markers of skeletal muscle hypertrophy with resistance training in previously untrained, college-aged males. A secondary aim was to also assess how these different supplements affected subcutaneous (SQ) fat cell size from biopsy specimens given that recent data from our group has demonstrated that WPH can elicit lipolytic effects [10,18,19]. Notably, the servings from all supplement groups (except the maltodextrin placebo described below) were standardized for LEU content (~3 grams per serving) and ingested twice daily.

## Study design and Methodology

*Study design*

The study design implemented was double-blinded and placebo-controlled (Figure 1). Participants were encouraged to refrain from rigorous physical activity for 4-5 days prior to baseline testing (T1). For T1, participants were instructed to report to the laboratory in a well-hydrated, 4-hour fasted state whereby they were subjected to the following assessments: a) urine specific gravity, b) height and body mass, c) body composition using dual-energy X-ray absorptiometry (DXA) (General Electric Lunar Prodigy enCORE, software version 10.50.086; Madison, WI, USA), d) vastus lateralis thickness using ultrasonography (General Electric LOGIQ S7 Expert; Chicago, IL, USA), e) venipuncture, f) percutaneous skeletal muscle biopsy collection from the vastus lateralis, and g) a percutaneous SQ fat biopsy from the gluteal region. Two to three days following T1, subjects reported back to the laboratory in a 4-hour fasted state for a second visit (T2) whereby maximal force production capacity was assessed using an isometric mid-thigh pull (IMTP) test, lower body strength was assessed using a three repetition maximum (3-RM) squat, and upper body strength was assessed using a 3-RM bench press. Additionally, during T2, subjects were familiarized with all lifts that were to be performed during the training intervention. Following T2, subjects engaged in 12 weeks of resistance training and supplementation. The last training bout (T38) consisted of IMTP as well as squat and bench press 3-RM re-assessments in a 4-hour fasted state. Seventy two hours following T38, subjects reported back to the laboratory in a 4-hour fasted state for post-testing (T39) which consisted of all body composition, and blood and biopsy collection procedures noted for T1. All of the aforementioned testing procedures as well as training and supplementation procedures are described in greater detail below.

Figure 1. Study design

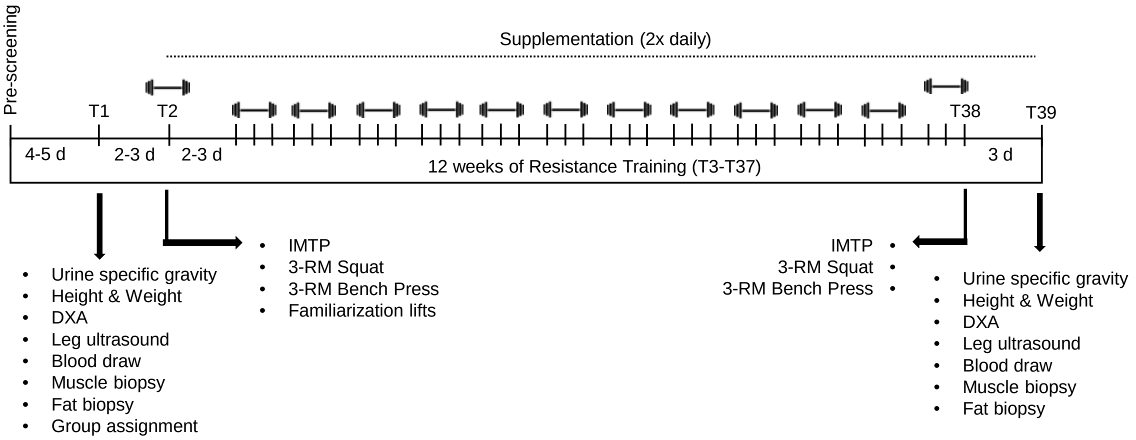


Abbreviations: DXA, dual x-ray absorptiometry; 3-RM, three-repetition maximum test; IMTP, isometric mid-thigh pull.

*Body Composition Testing*

During T1 and T39 participants were instructed to submit a urine sample (~5 mL) to assess normal hydration specific gravity levels (1.005-1.020 ppm) using a handheld refractometer (ATAGO; Bellevue, WA, USA). Participants with a urine specific gravity $\geq$1.020 were asked to consume tap water every 15 min for 30 min and then were re-tested. Following hydration testing, height and body mass were assessed using a digital column scale (Seca 769; Hanover, MD, USA) with weights and heights collected to the nearest 0.1 kg and 0.5 cm, respectively. Next, participants were subjected to a full body DXA scan while wearing general sports attire (i.e., athletic shorts or compression shorts and an athletic shirt) to assess various body composition characteristics. Dual arm and dual leg lean muscle mass, as assessed by the accompanying software, were used to estimate total body skeletal muscle mass (TBMM) by employing the equation from Kim et al. [20], as reported by our group previously [10]. Notably, body segmentation for each scan was standardized prior to analyses by the same technician. Total body fat mass was also assessed by the accompanying software. According to previous data published by our laboratory, the same-day reliability of the DXA during a test-calibrate-retest on 10 participants produced intra-class correlation coefficients of 0.998 for total body fat mass (mean difference between tests = 0.40±0.05 kg), 0.998 for total body lean mass (mean difference between tests = 0.29±0.13 kg), and 0.998 for dual-leg lean mass (mean difference between tests = 0.17±0.09 kg) [21].

Following DXA scans, participants were subjected to an ultrasound assessment to determine vastus lateralis muscle thickness. Measurements were taken from the midway point between the iliac crest and patella of the right femur whereby subjects were in a standing position and all weight was placed on the left leg. All DXA scans and ultrasound assessments were completed by the same investigator as suggested by previous research interventions [10,22] in order to minimize variability in testing procedures.

*Venipuncture, and percutaneous skeletal muscle and fat biopsies*

T1 and T39 venous blood samples were aseptically collected from the antecubital vein and collected into a 5 mL serum separator tube (BD Vacutainer; Franklin Lakes, NJ, USA). Notably, this blood was saved for further experimentation and variables assessed from these blood draws are not presented herein. Immediately following blood collection, participants were

instructed to lay in a supine position on a treatment table whereby a percutaneous skeletal muscle biopsy was aseptically obtained from the left vastus lateralis muscle using a 5 gauge Bergstöm needle with suction as previously described by our laboratory [23-26]. Approximately, 20-40 mg of skeletal muscle tissue for each time point was placed in a cryomold with OCT media (Electron Microscopy Sciences; Hatfield, PA, USA). Cryomolds were then slow-frozen in liquid-nitrogen-cooled isopentane and stored at -80°C for immunohistochemistry analyses that are described below. Sections of SQ fat (1-2 cm) extracted from the gluteal aspect of the left hip were placed in 10% formalin and preserved for hematoxylin and eosin (H&E) staining and histological analyses which are described in detail below. Following T1 testing procedures, subjects were counterbalanced into one of five groups based upon DXA LBM values in order to ensure that baseline values did not differ between supplement groups. More details regarding supplementation are described below, and supplementation began immediately following the first training bout (T3).

*Isometric mid-thigh pull, strength testing, and weightlifting familiarization*

During T2 and T38, participants were instructed to report back to the laboratory under well hydrated, 4-hour fasted conditions for strength testing and weight training familiarization (T2). First, each participant completed an IMTP test which has been validated to approximate whole-body maximal voluntary strength [27-29]. Briefly, knee and hip angles (125±5° and 175±5°, respectively) were measured using a standard goniometer (Fabrication Enterprises; White Plains, NY, USA). A standard, 20 kg barbell (York Barbell; York, PA, USA) and STS Power Rack (York Barbell) were used to conduct the IMTP. Dual OR6-7 force plates (AMTI; Watertown, MA, USA) with dual Gen 5 amplifiers (AMTI) sampling at 500 Hz were used to measure vertical force production in Newtons (N). Similar to other investigations [29-32], each participant was allowed at least two attempts, and up to four attempts if differences in vertical peak force between trials was >250 N. Manufacturer software was used to calculate vertical peak force during the testing sessions, and a custom-written MATLAB script (Natick, MA, USA) was employed to identify the greatest vertical force produced in N for each trial, post-hoc. Two trials within 250 N were used to calculate an average vertical peak force across trials and functioned as a metric for maximal voluntary force production (i.e., strength) in this investigation.

Approximately 5 min following T2 and T38 IMTP testing, participants performed 3-RM back squat and bench press assessments using a 20 kg barbell (York Barbell), STS Power Rack (York Barbell) and free weights. The demonstration of proper technique as well as the implementation of progressively-loaded 3-RM tests were overseen by C.B.M. and C.T.H. who possess the Certified Strength and Conditioning Specialist (CSCS) credential from the National Strength and Conditioning Association (NSCA). Bench press and back squat repetitions were considered to be successful when performed through the full range of motion (i.e., chest touch to full arm extension for bench press, and eccentric lowering past 90° knee flexion for back squat). A repetition was not counted if subjects exhibited poor and/or unsafe technique or needed assistance with a repetition during maximal testing.

Approximately 5 min following T2 3-RM testing, participants were instructed to perform the other two major lifts that were implemented for training (i.e., deadlift and bent-over-row) in the presence of CSCS-certified personnel. The goal of this session was to familiarize each participant with appropriate lifting technique in order to minimize the risk of injury throughout the course of the study.

*Training protocol*

For visits 3-37 (T3-T37), a daily undulating periodization (DUP) training model was employed over the 12-week training period given that this model has been shown to be more beneficial in eliciting greater increases in strength [33,34] and hypertrophy [35,36] than traditional linear periodization training models. Specifically, participants were instructed to perform free-weighted barbell squats, bench press, deadlifts, and bent-over-rows for 4 sets of 10 repetitions (Monday or Tuesday), 6 sets of 4 repetitions (Wednesday or Thursday), and 5 sets of 6 repetitions (Friday or Sunday). Immediately following each completed set, a rating of perceived exertion (RPE) score was acquired from each participant (scale: 1-10) in order to monitor and progress each participant accordingly while minimizing the potential risk of injury [37-40]. The RPE scale was described to participants as the remaining number of repetitions that the participant would be able to complete while employing good technique (i.e., 1 = 9 remaining repetitions in reserve, 10 = 0 remaining repetitions in reserve). More information on relative training intensities and progression can be found in Table 1. Participants were instructed to attend all 36 resistance training sessions throughout the duration of the study, but those that missed more than 4 sessions were not included in the analysis due to lack of training compliance. All participants were supervised by laboratory personnel for each training session to ensure that proper lifting technique was executed, and training volumes for each session were recorded.

Table 1. Training load progression

| **Week** | **Training Paradigm** | |
|---|---|---|
| 0 | Days 1-3 | Familiarization session, IMTP & 3-RM Testing |
| 1 | Day 1: 4x10 | 51 % of Est. 1-RM |
| | Day 2: 6x4 | 60 % of Est. 1-RM |
| | Day 3: 5x6 | 56 % of Est. 1-RM |
| 2 | Day 1: 4x10 | 60 % of Est. 1-RM |
| | Day 2: 6x4 | 70 % of Est. 1-RM |
| | Day 3: 5x6 | 65 % of Est. 1-RM |
| 3 | Day 1: 4x10 | 70 % of Est. 1-RM |
| | Day 2: 6x4 | 79 % of Est. 1-RM |
| | Day 3: 5x6 | 74 % of Est. 1-RM |
| 4 | Day 1: 4x10 | 73 % of Est. 1-RM |
| | Day 2: 6x4 | 89 % of Est. 1-RM |
| | Day 3: 5x6 | 84 % of Est. 1-RM |
| 5 | Day 1: 4x10 | 78 % of Est. 1-RM |
| | Day 2: 6x4 | 95 % of Est. 1-RM |
| | Day 3: 5x6 | 90 % of Est. 1-RM |
| 6 | Day 1: 4x10 | 82 % of Est. 1-RM |
| | Day 2: 6x4 | 100 % of Est. 1-RM |
| | Day 3: 5x6 | 94 % of Est. 1-RM |
| 7 | Day 1-3: 4x5 (de-load) | 60% of Est. 1-RM |

| | | |
|---|---|---|
| 8 | Day 1: 4x10 | 74 % of Est. 1-RM |
| | Day 2: 6x4 | 90 % of Est. 1-RM |
| | Day 3: 5x6 | 85 % of Est. 1-RM |
| 9 | Day 1: 4x10 | 83 % of Est. 1-RM |
| | Day 2: 6x4 | 101 % of Est. 1-RM |
| | Day 3: 5x6 | 96 % of Est. 1-RM |
| 10 | Day 1: 4x10 | 87 % of Est. 1-RM |
| | Day 2: 6x4 | 107 % of Est. 1-RM |
| | Day 3: 5x6 | 98 % of Est. 1-RM |
| 11 | Day 1: 4x10 | 90 % of Est. 1-RM |
| | Day 2: 6x4 | 109 % of Est. 1-RM |
| | Day 3: 5x6 | 102 % of Est. 1-RM |
| 12 | Day 1-2: 4x5 (de-load) | 60% of Est. 1-RM |
| | Day 3: IMTP & 3-RM Testing | 108% of Est. 1-RM |

Legend: Estimated one repetition maximum (Est. 1-RM) was calculated per the NSCA's recommended guidelines (i.e., 3-RM/0.93). Abbreviations: IMTP, isometric mid-thigh pull; 3-RM three repetition maximum; 1-RM, one repetition maximum; NSCA, National Strength and Conditioning Association.

*Supplementation*

As stated above, participants were assigned to ingest either a PLA, LEU, WPC, WPH, or SPC supplement throughout the training intervention. More information regarding the macronutrient profile for a serving size of each supplement can be found in Table 2. On training days (T3-T37), participants consumed an individually-packaged serving in ~500 ml of tap water immediately following each training session under direct observation of the study personnel. Additionally, participants were instructed to consume an individual serving within 30 min prior to bedtime on training days given that this strategy has been shown to be effective for stimulating overnight muscle protein synthesis [41]. On non-training days, participants were instructed to consume an individual serving between a meal of their choosing and 30 min prior to bedtime. Supplements were separated into individual ready-made supplement-coded packets for daily consumption, and participants were given a 3-week supply. Study personnel collected and counted empty packets from each participant every 3 weeks before the next 3-week supply was distributed. Participants that did not consume $\geq$80% were not included in the analysis due to lack of compliance.

Each supplement, except PLA, was formulated to provide ~3 g of leucine, per serving. Furthermore, each supplement was formulated to yield similar amounts of total energy (kcal) and fat (g), and was double-blinded to laboratory personnel and participants for group, appearance, taste, texture, and packaging. The WPC supplement was formulated using an agglomerated, 80% WPC (Hilmar™ 8010, Hilmar Ingredients; Hilmar, CA, USA). The WPH supplement was formulated using an agglomerated, partially hydrolyzed [12.5% degree of hydrolysate (12.5% DH), yielding approximately 67% of peptides as <5 kilodaltons (kDa) in molecular weight] 80% whey protein concentrate (Hilmar™ 8360, Hilmar Ingredients); SPC used an agglomerated, 80% soy protein concentrate (ALPHA® 5812, Solae, LLC; St. Louis, MO, USA); LEU used an agglomerated, L-Leucine (L-Leucine USP, Glambia Nutritionals; Carlsbad, CA, USA) and non-GMO, corn-derived maltodextrin (MALTRIN®-M100; Grain Processing Corporation; Muscantine, IA, USA); and, the PLA group was formulated using maltodextrin (MALTRIN®-

M100; Grain Processing Corporation). All five supplements were manufactured at JW Nutritional, LLC (Allen, TX, USA), a United States Food and Drug Administration cGMP-compliant facility independently audited and pre-qualified by Obvium*Q, LLC (Phoenix, AZ, USA), a GMP regulatory compliance firm. Personnel at JW Nutritional, LLC and C.M.L. (Lockwood, LLC; Draper, UT, USA) formulated and maintained blinding of groups, and each supplement was assigned a randomly generated item number. Manufacturing batch records for production of each of the five supplements were reviewed by a trained, independent expert in dietary supplement quality control and assurance (C.M.L.) before approval for use within the present study. All supplements were independently validated for nutritional facts and total amino acids using validated, approved methods at Covance Laboratories, Inc. (Madison, WI, USA), a pre-qualified third-party analytical laboratory, and results reviewed by C.M.L. prior to the supplements being approved for use within the present study. Once analysis was complete, a Lockwood, LLC representative not involved in the study released the code for all treatments.

Table 2. Nutritional components per serving for the different supplements

| Variable | PLA | LEU | WPC | WPH | SPC |
|---|---|---|---|---|---|
| Calories | 204 | 200 | 184 | 192 | 266 |
| Total Fat (g) | 2.8 | 2.0 | 3.5 | 4.6 | 4.5 |
| Saturated Fat (g) | 2.3 | 1.6 | 2.3 | 3.3 | 2.6 |
| Trans Fat (g) | 0.0 | 0.0 | 0.1 | 0.2 | 0.2 |
| Cholesterol (mg) | 3.8 | 2.9 | 74.0 | 74.3 | 5.3 |
| Total Carbohydrate (g) | 44.4 | 43.1 | 12.0 | 12.2 | 17.2 |
| Dietary Fiber (g) | 1.6 | 1.8 | 1.8 | 2.2 | 1.5 |
| Sugars (g) | 6.0 | 5.1 | 5.9 | 3.2 | 6.2 |
| Protein (g) | 0.4 | 2.3 | 26.3 | 25.4 | 39.2 |
| Alanine (mg) | 7 | 7 | 1,397 | 1,430 | 1,646 |
| Arginine (mg) | 8 | 8 | 766 | 773 | 2,969 |
| Aspartic Acid (mg) | 15 | 16 | 2,881 | 3,010 | 4,537 |
| Cystine (mg) | 0 | 0 | 651 | 728 | 536 |
| Glutamic Acid (mg) | 36 | 35 | 4,530 | 4,730 | 7,154 |
| Glycine (mg) | 6 | 6 | 489 | 543 | 1,597 |
| Histidine (mg) | 0 | 0 | 470 | 477 | 910 |
| Isoleucine (mg) | 8 | 28 | 1,736 | 1,820 | 1,842 |
| Leucine (mg) | 15 | 2,871 | 2,794 | 2,910 | 2,960 |
| Lysine (mg) | 11 | 79 | 2,386 | 2,640 | 2,362 |
| Methionine (mg) | 0 | 8 | 598 | 611 | 540 |
| Phenylalanine (mg) | 8 | 9 | 861 | 908 | 1,980 |
| Proline (mg) | 14 | 13 | 1,630 | 1,670 | 2,029 |
| Serine (mg) | 10 | 9 | 1,348 | 1,400 | 1,950 |
| Threonine (mg) | 7 | 7 | 1,853 | 1,900 | 1,499 |
| Tryptophan | 0 | 0 | 482 | 525 | 501 |
| Tyrosine (mg) | 7 | 7 | 808 | 839 | 1,480 |
| Valine (mg) | 11 | 14 | 1,465 | 1,530 | 1,754 |
| Total EAAs (mg) | 60 | 3,016 | 12,645 | 13,321 | 14,348 |
| Total BCAAs (mg) | 34 | 2,913 | 5,995 | 6,260 | 6,556 |
| Calcium (mg) | 15 | 15 | 155 | 152 | 165 |
| Iron (mg) | 0.38 | 0.35 | 0.63 | 1.04 | 5.21 |

| | | | | | |
|---|---|---|---|---|---|
| Potassium (mg) | 32 | 37 | 230 | 464 | 961 |
| Sodium (mg) | 91 | 105 | 133 | 310 | 217 |
| Vitamin D3 (IU) | 0.0 | 0.0 | 0.0 | 0.0 | 0.0 |
| Degree of hydrolysis (%) | N/A | N/A | N/A | 12.5 | N/A |
| M.W. range (%) | | | | | |
| >10.0 kD | - | - | 74.3 | 29.0 | 86.0 |
| 5.0-10.0 kD | - | - | 5.1 | 5.3 | 3.6 |
| 2.0-5.0 kD | - | - | 15.4 | 10.2 | 2.6 |
| 1.0-2.0 kD | - | - | 1.6 | 10.8 | 1.2 |
| 0.5-1.0 kD | - | - | 0.9 | 15.5 | 0.9 |
| <0.5 kD | - | - | 2.7 | 29.3 | 5.6 |

Abbreviations: PLA, maltodextrin placebo; LEU, L-leucine; WPC, whey protein concentrate; WPH, whey protein hydrolysate; SPC, soy protein concentrate; g, grams; mg, milligrams; IU, international units; kD, kilodaltons.

*Nutritional intake monitoring*

Participants were instructed to maintain their normal dietary habits along with returning a 4-day food log (2 week days and both weekend days) at baseline (T1), week 6 (T20) and week 12 (T39). On each occasion, participants were given detailed written and verbal instructions on completing the food logs. Dietary intake data were analyzed using open-sourced software (http://www.myfitnesspal.com), which has been employed to analyze food intake data in other studies [42-48].

**Safety considerations**

In the event that a subject suffered an injury from training, this was immediately reported to Dr. Michael Goodlett (MD) who is the medical oversight of the Auburn University IRB. Additionally, any biopsy-related issues (e.g., hematomas) were reported to Dr. Goodlett. One participant did sustain an injury during this study, and he was referred to Dr. Goodlett. Additionally, this adverse event was filed with the Auburn IRB. This individual has since recovered from this injury.

**Follow-up**

Following the conclusion of training, the subjects were no longer required to report to the laboratory unless they had issues from the biopsy sites (e.g., hematoma formation or swelling). Notably, there were no adverse events related to biopsies, and no subjects reported adverse events related to being a participant in the study.

**Data Management and Statistical Analysis**

For the current paper submitted to Scientific Reports analyzing the SPC, WPC and PLA groups statistical tests were performed in RStudio (Version 1.0.143) and SPSS (Version 23). The average sample size for each dependent variable was 33 subjects, while specific sample sizes for each analysis are reported in figures or text. Group (3 levels [PLA, SPC, WPC]) and time (2 levels [PRE, POST]) served as independent variables in this analysis. Measurement of each

dependent variable occurred at each time point. *A-priori* power analyses in RStudio using general linear model parameters in the “pwr” package (Version 1.2-1) revealed 79 % power (power = 1 – β) for the discovery of a medium size effect, and 98 % power for the discovery of a large effect when analyses included 33 subjects with 2 observations (i.e., 66 total observations). Supplementary tables provide descriptive statistics for each dependent variable with no outlier removal, mean differences, Cohen’s *d* effect sizes, and 95 % confidence intervals. Data are not restated in results when included in figures. Data are reported as mean ± standard error of the mean, and are based on any data removal described below. The alpha level of significance was set a-priori to: $p \leq 0.050$. Statistical assumptions tests were completed prior to statistical analysis consisting of: a) Shapiro-Wilks tests of residual distributions for normality, and b) Levene’s test of homogeneity of variance, given that a repeated measures analysis of variance (ANOVA) was performed for the provision of p-values. Violation of these assumptions are reported and appropriate data transformations (i.e., square root or $\log_{10}$ transformations) were completed when residuals were not normally distributed prior to ANOVA for the avoidance of type 1 or type 2 errors. Data transformation and data removal were avoided with intention to report raw data and associated inferential statistics. For this reason, if the majority of levels of group (2 of 3 groups) at each level of time were normally distributed, ANOVA proceeded. However, if datum met both of the following two criteria, datum were removed to best reflect group responses and more confidently infer effects: 1) Shapiro-Wilks tests revealed non-normal distributions for the data array and, 2) the datum was ≥ 3 standard deviations from the group mean. Although rare, cases of data removal were reported. For significant group × time interactions, two-tailed independent samples t-tests were performed as a post-hoc test. For significant time *F*-statistics identified from ANOVA, a 2-tailed dependent samples t-test was performed comparing PRE to POST data arrays.

**Quality assurance**

All data obtained from training was kept in de-identified participant folders with study codes (e.g., “Subject 1”). Data entry was conducted by laboratory personnel, and data entry was double-checked by other laboratory personnel along with the principal investigator. Data entry from biochemical assays were entered by a graduate student, and these data were double-checked by the principal investigator.

**Expected outcomes of the project**

Based upon the supporting literature, we hypothesized that individuals consuming whey protein supplements would experience greater increases in indices related to muscle anabolism compared to those consuming the LEU, SPC and PLA supplements.

**Duration of the project**

This project was completed between August of 2016 and May of 2017.

**Problems anticipated**

Only one subject had to be withdrawn from the study due to an injury sustained during training. Other subjects were removed due to compliance issues (e.g., not training).

**Project management**

Michael D. Roberts, PhD: principal investigator, responsible for procuring biopsies, IRB reporting, and manuscript preparation

Christopher B. Mobley, PhD: study coordinator, responsible for overseeing the recruitment, training, and biochemical analysis

Cody T. Haun, MS: responsible for overseeing the recruitment, training, and biochemical analysis

**Ethics**

Prior to initiating this study, the protocol was reviewed and approved by the Auburn University Institutional Review Board (IRB), and was conducted in accordance with the Declaration of Helsinki (approved protocol #: 15-320 MR 1508; IRB contact: irbadmin@auburn.edu). Healthy, untrained, college-aged male (i.e., 19-23) participants were recruited for this study. All enrolled participants provided verbal and written study consent, completed a medical history form, and were given a 4-day food log to complete prior to initiating the study. These screening forms ensured that all eligible participants were healthy and recreationally active but: a) had not engaged in any regular exercise program for at least 6 months prior to study initiation ($\leq$2 resistance training exercise or high-intensity aerobic exercise sessions/week), b) were not currently consuming a high-protein diet ($\geq$2.0 g/kg/d), c) were not using anabolic enhancing agents (e.g., anabolic steroids, supplemental protein, creatine monohydrate, or prohormones), or d) did not have medical or orthopedic condition(s) that would hinder them from participating in the current study. Once initial screening was complete, all eligible participants were scheduled to return to the School of Kinesiology at Auburn University one week later for baseline testing (T1).

**Informed Consent Forms**

See appended document following this section.

**Budget**

Participant compensation along with reagents for the study were made possible through gift funds donated to Dr. Roberts' laboratory through Hilmar Ingredients (Hilmar, CA, USA) and Bionutritional Research Group (Irvine, CA, USA). More information regarding these explicit costs can be obtained by contacting the principal investigator at mdr0024@auburn.edu.

**Other support for the project**

N/A

**Collaboration with other scientists of research institutions**

N/A

**Curriculum Vitae of Dr. Roberts**
See appended document following this section.

**Other research activities of the investigator**
Refer to curriculum vitae for involvement in other contract- or grant-funded research.

**Financing and insurance**
N/A

**References**


1 Cribb, P. J., Williams, A. D., Carey, M. F. & Hayes, A. The effect of whey isolate and resistance training on strength, body composition, and plasma glutamine. *Int J Sport Nutr Exerc Metab* **16**, 494-509 (2006).

2 Burke, D. G. *et al.* The effect of whey protein supplementation with and without creatine monohydrate combined with resistance training on lean tissue mass and muscle strength. *Int J Sport Nutr Exerc Metab* **11**, 349-364 (2001).

3 Candow, D. G., Burke, N. C., Smith-Palmer, T. & Burke, D. G. Effect of whey and soy protein supplementation combined with resistance training in young adults. *Int J Sport Nutr Exerc Metab* **16**, 233-244 (2006).

4 Hulmi, J. J. *et al.* Acute and long-term effects of resistance exercise with or without protein ingestion on muscle hypertrophy and gene expression. *Amino Acids* **37**, 297-308, doi:10.1007/s00726-008-0150-6 (2009).

5 Volek, J. S. *et al.* Whey protein supplementation during resistance training augments lean body mass. *J Am Coll Nutr* **32**, 122-135, doi:10.1080/07315724.2013.793580 (2013).

6 Paoli, A. *et al.* Protein Supplementation Does Not Further Increase Latissimus Dorsi Muscle Fiber Hypertrophy after Eight Weeks of Resistance Training in Novice Subjects, but Partially Counteracts the Fast-to-Slow Muscle Fiber Transition. *Nutrients* **8**, doi:10.3390/nu8060331 (2016).

7 Reidy, P. T. *et al.* Protein Supplementation Does Not Affect Myogenic Adaptations to Resistance Training. *Med Sci Sports Exerc* **49**, 1197-1208, doi:10.1249/MSS.0000000000001224 (2017).

8 Morton, R. W. *et al.* A systematic review, meta-analysis and meta-regression of the effect of protein supplementation on resistance training-induced gains in muscle mass and strength in healthy adults. *Br J Sports Med*, doi:10.1136/bjsports-2017-097608 (2017).

9 Reidy, P. T. *et al.* Protein Supplementation Has Minimal Effects on Muscle Adaptations during Resistance Exercise Training in Young Men: A Double-Blind Randomized Clinical Trial. *J Nutr* **146**, 1660-1669, doi:10.3945/jn.116.231803 (2016).

10 Lockwood, C. M. *et al.* Effects of Hydrolyzed Whey versus Other Whey Protein Supplements on the Physiological Response to 8 Weeks of Resistance Exercise in

College-Aged Males. *J Am Coll Nutr* **36**, 16-27, doi:10.1080/07315724.2016.1140094 (2017).
11 Bird, S. P., Tarpenning, K. M. & Marino, F. E. Independent and combined effects of liquid carbohydrate/essential amino acid ingestion on hormonal and muscular adaptations following resistance training in untrained men. *Eur J Appl Physiol* **97**, 225-238, doi:10.1007/s00421-005-0127-z (2006).
12 Vieillevoye, S., Poortmans, J. R., Duchateau, J. & Carpentier, A. Effects of a combined essential amino acids/carbohydrate supplementation on muscle mass, architecture and maximal strength following heavy-load training. *Eur J Appl Physiol* **110**, 479-488, doi:10.1007/s00421-010-1520-9 (2010).
13 Aguiar, A. F. *et al.* Free leucine supplementation during an 8-week resistance training program does not increase muscle mass and strength in untrained young adult subjects. *Amino Acids* **49**, 1255-1262, doi:10.1007/s00726-017-2427-0 (2017).
14 Phillips, S. M., Tang, J. E. & Moore, D. R. The role of milk- and soy-based protein in support of muscle protein synthesis and muscle protein accretion in young and elderly persons. *J Am Coll Nutr* **28**, 343-354 (2009).
15 Tang, J. E., Moore, D. R., Kujbida, G. W., Tarnopolsky, M. A. & Phillips, S. M. Ingestion of whey hydrolysate, casein, or soy protein isolate: effects on mixed muscle protein synthesis at rest and following resistance exercise in young men. *J Appl Physiol (1985)* **107**, 987-992, doi:10.1152/japplphysiol.00076.2009 (2009).
16 Atherton, P. J., Smith, K., Etheridge, T., Rankin, D. & Rennie, M. J. Distinct anabolic signalling responses to amino acids in C2C12 skeletal muscle cells. *Amino Acids* **38**, 1533-1539, doi:10.1007/s00726-009-0377-x (2010).
17 Norton, L. E. *et al.* The leucine content of a complete meal directs peak activation but not duration of skeletal muscle protein synthesis and mammalian target of rapamycin signaling in rats. *J Nutr* **139**, 1103-1109, doi:10.3945/jn.108.103853 (2009).
18 Mobley, C. B. *et al.* Effects of protein type and composition on postprandial markers of skeletal muscle anabolism, adipose tissue lipolysis, and hypothalamic gene expression. *J Int Soc Sports Nutr* **12**, 14, doi:10.1186/s12970-015-0076-9 (2015).
19 Roberts, M. D. *et al.* Comparing serum responses to acute feedings of an extensively hydrolyzed whey protein concentrate versus a native whey protein concentrate in rats: a metabolomics approach. *Appl Physiol Nutr Metab* **39**, 158-167, doi:10.1139/apnm-2013-0148 (2014).
20 Kim, J., Wang, Z., Heymsfield, S. B., Baumgartner, R. N. & Gallagher, D. Total-body skeletal muscle mass: estimation by a new dual-energy X-ray absorptiometry method. *Am J Clin Nutr* **76**, 378-383 (2002).
21 Kephart, W. C. *et al.* Ten weeks of branched-chain amino acid supplementation improves select performance and immunological variables in trained cyclists. *Amino Acids* **48**, 779-789, doi:10.1007/s00726-015-2125-8 (2016).
22 Lohman, M., Tallroth, K., Kettunen, J. A. & Marttinen, M. T. Reproducibility of dual-energy x-ray absorptiometry total and regional body composition measurements using different scanning positions and definitions of regions. *Metabolism* **58**, 1663-1668, doi:10.1016/j.metabol.2009.05.023 (2009).
23 Kephart, W. C. *et al.* A single bout of whole-leg, peristaltic pulse external pneumatic compression upregulates PGC-1alpha mRNA and endothelial nitric oxide sythase protein

in human skeletal muscle tissue. *Exp Physiol* **100**, 852-864, doi:10.1113/EP085160 (2015).
24 Martin, J. S. *et al.* A single 60-min bout of peristaltic pulse external pneumatic compression transiently upregulates phosphorylated ribosomal protein s6. *Clin Physiol Funct Imaging*, doi:10.1111/cpf.12343 (2016).
25 Martin, J. S. *et al.* Impact of external pneumatic compression target inflation pressure on transcriptome-wide RNA expression in skeletal muscle. *Physiol Rep* **4**, doi:10.14814/phy2.13029 (2016).
26 Haun, C. T. *et al.* Does external pneumatic compression treatment between bouts of overreaching resistance training sessions exert differential effects on molecular signaling and performance-related variables compared to passive recovery? An exploratory study. *PLoS One* **12**, e0180429, doi:10.1371/journal.pone.0180429 (2017).
27 Nuzzo, J. L., McBride, J. M., Cormie, P. & McCaulley, G. O. Relationship between countermovement jump performance and multijoint isometric and dynamic tests of strength. *J Strength Cond Res* **22**, 699-707, doi:10.1519/JSC.0b013e31816d5eda (2008).
28 Wang, R. *et al.* Isometric Mid-Thigh Pull Correlates With Strength, Sprint, and Agility Performance in Collegiate Rugby Union Players. *J Strength Cond Res* **30**, 3051-3056, doi:10.1519/JSC.0000000000001416 (2016).
29 Beckham, G. *et al.* Relationships of isometric mid-thigh pull variables to weightlifting performance. *J Sports Med Phys Fitness* **53**, 573-581 (2013).
30 Kraska, J. M. *et al.* Relationship between strength characteristics and unweighted and weighted vertical jump height. *Int J Sports Physiol Perform* **4**, 461-473 (2009).
31 Leary, B. K. *et al.* The relationship between isometric force-time curve characteristics and club head speed in recreational golfers. *J Strength Cond Res* **26**, 2685-2697, doi:10.1519/JSC.0b013e31826791bf (2012).
32 Haff, G. G., Ruben, R. P., Lider, J., Twine, C. & Cormie, P. A comparison of methods for determining the rate of force development during isometric midthigh clean pulls. *J Strength Cond Res* **29**, 386-395, doi:10.1519/JSC.0000000000000705 (2015).
33 Rhea, M. R., Ball, S. D., Phillips, W. T. & Burkett, L. N. A comparison of linear and daily undulating periodized programs with equated volume and intensity for strength. *J Strength Cond Res* **16**, 250-255 (2002).
34 Prestes, J. *et al.* Comparison between linear and daily undulating periodized resistance training to increase strength. *J Strength Cond Res* **23**, 2437-2442, doi:10.1519/JSC.0b013e3181c03548 (2009).
35 Klemp, A. *et al.* Volume-equated high- and low-repetition daily undulating programming strategies produce similar hypertrophy and strength adaptations. *Appl Physiol Nutr Metab* **41**, 699-705, doi:10.1139/apnm-2015-0707 (2016).
36 Simao, R. *et al.* Comparison between nonlinear and linear periodized resistance training: hypertrophic and strength effects. *J Strength Cond Res* **26**, 1389-1395, doi:10.1519/JSC.0b013e318231a659 (2012).
37 Day, M. L., McGuigan, M. R., Brice, G. & Foster, C. Monitoring exercise intensity during resistance training using the session RPE scale. *J Strength Cond Res* **18**, 353-358, doi:10.1519/R-13113.1 (2004).
38 Impellizzeri, F. M., Rampinini, E., Coutts, A. J., Sassi, A. & Marcora, S. M. Use of RPE-based training load in soccer. *Med Sci Sports Exerc* **36**, 1042-1047 (2004).

39 Zourdos, M. C. *et al.* Novel Resistance Training-Specific Rating of Perceived Exertion Scale Measuring Repetitions in Reserve. *J Strength Cond Res* **30**, 267-275, doi:10.1519/JSC.0000000000001049 (2016).
40 Veugelers, K. R., Young, W. B., Fahrner, B. & Harvey, J. T. Different methods of training load quantification and their relationship to injury and illness in elite Australian football. *J Sci Med Sport* **19**, 24-28, doi:10.1016/j.jsams.2015.01.001 (2016).
41 Trommelen, J. *et al.* Pre-sleep dietary protein-derived amino acids are incorporated in myofibrillar protein during post-exercise overnight recovery. *Am J Physiol Endocrinol Metab*, ajpendo 00273 02016, doi:10.1152/ajpendo.00273.2016 (2017).
42 Waldron, M. *et al.* The effects of acute branched-chain amino acid supplementation on recovery from a single bout of hypertrophy exercise in resistance-trained athletes. *Appl Physiol Nutr Metab* **42**, 630-636, doi:10.1139/apnm-2016-0569 (2017).
43 Schoenfeld, B. J., Contreras, B., Vigotsky, A. D. & Peterson, M. Differential Effects of Heavy Versus Moderate Loads on Measures of Strength and Hypertrophy in Resistance-Trained Men. *J Sports Sci Med* **15**, 715-722 (2016).
44 Jospe, M. R., Fairbairn, K. A., Green, P. & Perry, T. L. Diet app use by sports dietitians: a survey in five countries. *JMIR Mhealth Uhealth* **3**, e7, doi:10.2196/mhealth.3345 (2015).
45 Antonio, J. *et al.* A high protein diet (3.4 g/kg/d) combined with a heavy resistance training program improves body composition in healthy trained men and women--a follow-up investigation. *J Int Soc Sports Nutr* **12**, 39, doi:10.1186/s12970-015-0100-0 (2015).
46 Antonio, J., Peacock, C. A., Ellerbroek, A., Fromhoff, B. & Silver, T. The effects of consuming a high protein diet (4.4 g/kg/d) on body composition in resistance-trained individuals. *J Int Soc Sports Nutr* **11**, 19, doi:10.1186/1550-2783-11-19 (2014).
47 Antonio, J., Ellerbroek, A., Silver, T., Vargas, L. & Peacock, C. The effects of a high protein diet on indices of health and body composition--a crossover trial in resistance-trained men. *J Int Soc Sports Nutr* **13**, 3, doi:10.1186/s12970-016-0114-2 (2016).
48 Antonio, J. *et al.* A High Protein Diet Has No Harmful Effects: A One-Year Crossover Study in Resistance-Trained Males. *J Nutr Metab* **2016**, 9104792, doi:10.1155/2016/9104792 (2016).

AUBURN UNIVERSITY INSTITUTIONAL REVIEW BOARD for RESEARCH INVOLVING HUMAN SUBJECTS

# REQUEST for MODIFICATION

For help, contact: **THE OFFICE OF RESEARCH COMPLIANCE (ORC)**, 115 Ramsay Hall, Auburn University

**Phone:** 334-844-5966 **e-mail:** IRBAdmin@auburn.edu **Web Address:** http://www.auburn.edu/research/vpr/ohs

Revised 2.1.2014 **Submit completed form to IRBsubmit@auburn.edu or 115 Ramsay Hall, Auburn University 36849.**

Form must be populated using Adobe Acrobat / Pro 9 or greater standalone program (do not fill out in browser). Hand written forms will not be accepted.

1. Protocol Number: 15-320 MR1508
2. Current IRB Approval Dates: From: 8-18-15 To: 8-18-16
3. Project Title: The Effects of Different Protein and Amino Acid Supplements on Muscle Hypertrophy after 12 Weeks of Resistance Exercise in Untrained Men
4. Michael D Roberts | Asst Prof | KINE | 4-1925 | mdr0024@auburn.edu

   Principal Investigator | Title | Department | Phone | AU E-Mail (primary)

   Mike Roberts Digitally signed by Mike Roberts DN: cn=Mike Roberts, o=Auburn University, ou=School of Kinesiology, email=mdr0024@auburn.edu, c=US Date: 2015.09.16 10:14:52 -05'00' | 301 Wire Road | 

   PI Signature | Mailing Address | Alternate E-Mail

   Faculty Advisor | FA Signature | Department | Phone | AU E-Mail

   Name of Current Department Head: Mary Rudisill AU E-Mail: rudisme@auburn.edu
5. Current External Funding Agency and Grant number: Gift donation from Bionutritional Research Group
6. a. List any contractors, sub-contractors, other entities associated with this project:

   b. List any other IRBs associated with this project:
7. Nature of change in protocol: (Mark all that apply)

- ☐ Change in Key Personnel (attach CITI forms for new personnel)
- ☐ Change in Sites (attach permission forms for new sites)
- ☐ Change in methods for data storage/protection or location of data/consent documents
- ☑ Change in project purpose or questions
- ☐ Change in population or recruitment (attach new or revised recruitment materials as needed)
- ☐ Change in consent procedures (attach new or revised consent documents as needed)
- ☑ Change in data collection methods or procedures (attach new data collection forms as needed)
- ☐ Other (explain):

**FOR ORC OFFICE USE ONLY**

**DATE RECEIVED IN ORC:**_____ **by**_____ **MODIFICATION #**

**DATE OF IRB REVIEW:**_____ **by**_____ **PROTOCOL APPR**

**DATE OF IRB APPROVAL:**_____ **by**_____ **MODIFICATI**

**INTERVAL F**

**COMMENTS:**

The Auburn University Institutional Review Board has approved this Document for use from 05/18/2016 to 08/18/2016
Protocol # 15-320 MR 1508

8. Briefly list *(numbered or bulleted)* the activities that have occurred up to this point, particularly those that involved participants.

None, we are beginning this study this upcoming summer.

9. For each item marked in Question #7, describe the requested changes to your research protocol, with an explanation and/or rationale for each. (*Additional pages may be attached if needed to provide a complete response.)*

This document is titled 'Proposed amendments for Protein study (15-320 MR1508)'

10. Identify any changes in the anticipated risks and / or benefits to the participants.

Additional anticipated risks: a) more 'soreness' from a full-body workout versus a leg workout, b) more soreness from the two additional fat biopsies

Additional anticipated benefits: compensation of $500 versus $300

11. Identify any changes in the safeguards or precautions that will be used to address anticipated risks.

Participants will be progressively trained (i.e., will not start with heavy weight-lifting, but will use lighter lifting loads and build up slowly to more challenging loads). Moreover, participants can discontinue the study if the workouts make them too sore. The same precautions will be taken with fat biopsies as with the muscle biopsies. Again, the participants can discontinue the study if the biopsies make them too sore. However, we have not had this issue in other IRB-approved studies with muscle biopsies and do not anticipate this being an issue with the fat biopsies.

12. Attach a copy of <u>all</u> "stamped" IRB-approved documents you are currently using. *(information letters, consents, flyers, etc.)*

Question #9:

<u>AMENDMENT REQUEST #1 (reducing the number of participants)</u>

The original purpose of the study was to determine muscle-building effects of supplementing with either 2 doses per day (dose amounts are in parentheses) of:

- whey protein concentrate (40 g/day twice daily) (n=15 participants per group were to be recruited)
- whey protein hydrolysate (40 g/day twice daily), (n=15 participants per group were to be recruited)
- egg protein concentrate (40 g/day twice daily), (n=15 participants per group were to be recruited)
- egg protein hydrolysate (40 g/day twice daily), (n=15 participants per group were to be recruited)
- micellar casein (40 g/day twice daily), (n=15 participants per group were to be recruited)
- soy protein concentrate (40 g/day twice daily), (n=15 participants per group were to be recruited)
- branched chain amino acids (6 g/day twice daily), (n=15 participants per group were to be recruited)
- and a maltodextrin placebo (6 g/day twice daily), (n=15 participants per group were to be recruited)

n=120 participants were to be recruited

We now only wish to test the following groups (not crossed out):

- whey protein concentrate (40 g/day twice daily),
- whey protein hydrolysate (40 g/day twice daily),
- ~~egg protein concentrate (40 g/day twice daily),~~
- ~~egg protein hydrolysate (40 g/day twice daily),~~
- ~~micellar casein (40 g/day twice daily),~~
- soy protein concentrate (40 g/day twice daily),
- branched chain amino acids (6 g/day twice daily),
- and a maltodextrin placebo (6 g/day twice daily)

n=75 participants will now be recruited, although with potential attrition we expect to recruit 120 participants.

**Rationale**: We are unable to procure egg and casein protein from industry sponsors. Notwithstanding, this study will still compare different whey protein supplements versus soy and amino acids supplements, and has extremely valuable implications in terms of which supplement is optimal for improving body composition.

AMENDMENT REQUEST #2 (alteration of the workout for participants)

Below is the original study design that is currently approved:

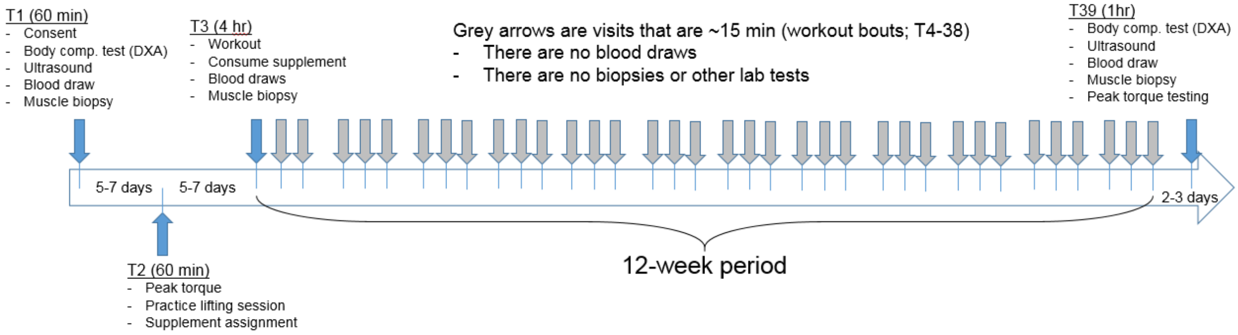


Each gray arrow is a workout to be completed in the School of Kinesiology. Each workout was originally 4-6 sets of leg extensions for 4-10 repetitions. Below shows the leg extensor exercise for frame of reference:

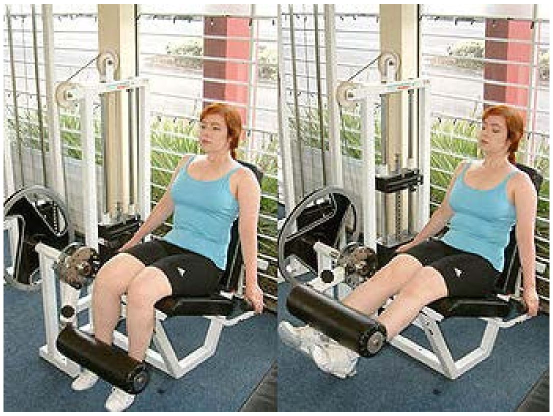

We now request that each workout be a 'full-body' (or 'whole-body') workout which will include:

- 3 sets of squats for 3-10 repetitions
- 3 sets of bench press for 3-10 repetitions
- 3 sets of bent-over rows for 3-10 repetitions
- 3 sets of deadlifts for 3-10 repetitions
- 3 sets of crunches for 3-10 repetitions

Below is a diagram of each exercise:

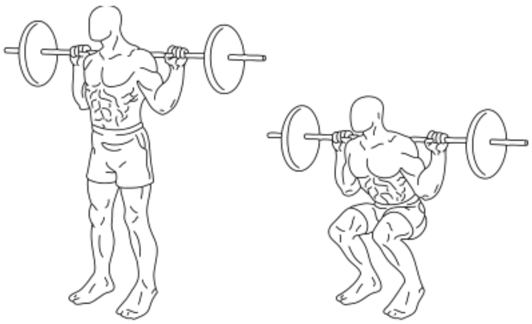

Squat

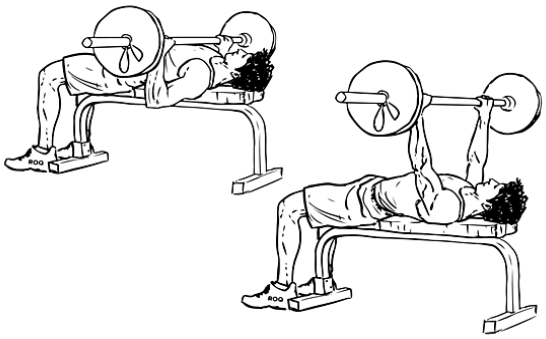

Bench press

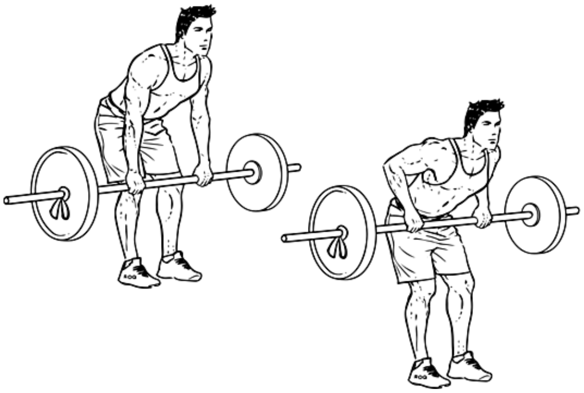

Rows

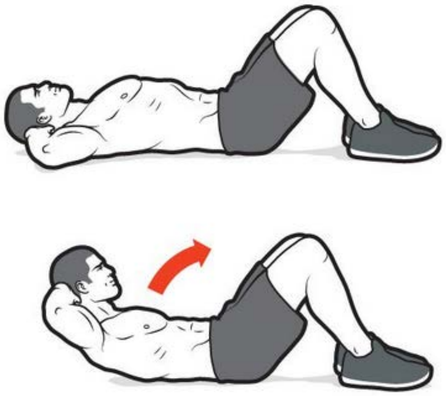

Crunches

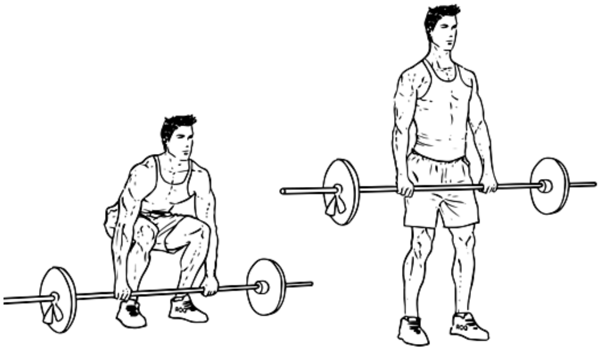

Deadlift

**Rationale**: The purpose of this request is to utilize a whole-body workout that has more beneficial whole-body effects rather than just focusing on the leg muscles with the originally-proposed leg extensor training.  This will better decipher if protein or amino acid supplementation affects whole-body composition versus just leg musculature.

AMENDMENT REQUEST #3 (obtaining 2 fat biopsies)

Below is the original study design that is currently approved:

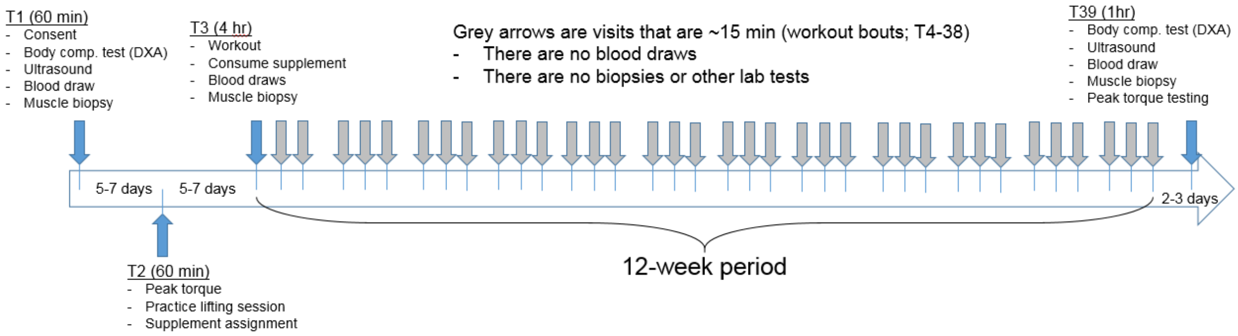


At T1, T3 and T39, there are muscle biopsies that are currently approved. We now wish to also obtain a fat biopsy from the leg as well at T1 and T39.

This process is very similar to the muscle biopsy in that it involves administering lidocaine to the collection site, making a pilot incision with a sterile blade, and placing the biopsy needle in the collection site. However, unlike the muscle biopsy, we will not penetrate the collection site as deep and only collect superficial adipose tissue. Below is a schematic that shows the differences between the two techniques:

Depth of biopsy needle during muscle biopsy

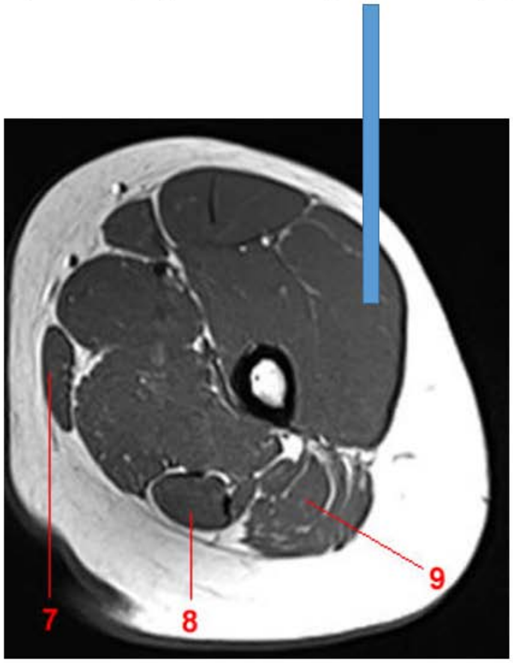


Depth of biopsy needle during fat biopsy

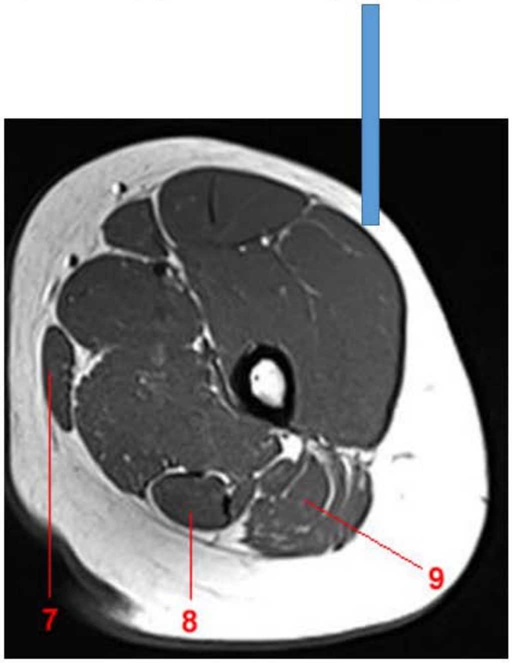


Importantly, fat biopsies are equally as safe as muscle biopsies. Moreover, Dr. Roberts has performed over 200 muscle biopsies to date here on Auburn's campus with zero post-biopsy adverse events being reported. Dr. Roberts had a meeting with Dr. Mike Goodlett about the fat biopsy procedure to ensure that Dr. Goodlett was fully aware of this proposed procedure.

The particular collection site will be the upper buttock region. Specifically, Dr. Roberts will sterilize the area as discussed with the muscle biopsy procedure and administer 1 cc of lidocaine. Dr. Roberts will then pinch a large area of fat in this area, make a pilot incision with a sterile blade, and penetrate the biopsy needle superficially in order to obtain a pencil erasure-size amount of fat tissue. Thereafter, a butterfly Band-Aid and large elastic bandage with gauze will be placed over the biopsy site. As with the 24-h checkup on the muscle biopsy, participants will have the fat biopsy collection site inspected as well.

**Rationale**: Whey protein supplementation has been shown to reduce body fat. Samples collected from the fat biopsy technique will be histologically examined in order to observe if fat cell size is reduced with whey protein supplementation versus the other supplements. Moreover, the fat samples will also be analyzed for the genetic expression of 'fat-building' genes. Our working hypothesis is that whey protein will be superior to soy protein as well as amino acid supplementation on reducing fat cell size and the expression of 'fat-building' genes.

AMENDMENT REQUEST #4 (subject compensation)

The original protocol approved subjects to be compensated $300. We now wish to pay participants $500 upon completion of the study. A new partial compensation will be as follows:

- $50 for completion of visits 1-2 (1 fat biopsy and 1 muscle biopsy given)
- An additional $100 for completion of visits 1-3 (1 fat biopsy and 2 muscle biopsies given)
- An additional $200 for completion of up to all visits except visit #39 (meaning that all workouts were completed but the last testing session was missed and, thus, the last muscle and fat biopsy was not taken)
- An additional $150 for completion of visit #39

Given that we are wishing to implement a more involved workout (full-body versus leg extensions only) and are wishing to collect 3 muscle biopsies (originally-approved) as well as two fat biopsies (AMENDMENT REQUEST #3 above), we feel that this is a fair compensation for participants.

# Opportunity to Participate in a Paid Research Study Titled:

### The Effects of Different Protein and Amino Acid Supplements on Muscle Hypertrophy after 12 Weeks of Resistance Exercise in Untrained Men

"Researchers in The School of Kinesiology at Auburn University are conducting a study which will evaluate which amino acid or protein source supplement (specifically branched chain amino acids, whey, or soy) enhances markers of skeletal muscle growth and body fat reduction when ingested in conjunction with chronic resistance training.

You may qualify if you:

1) are between the ages of 19-35 and have a body mass index (body mass in kilograms/ height in meters^2) between 23-30

2) are apparently healthy and do not have any known illnesses that are contraindicated for exercise (i.e., hypertension, arrhythmias, or cardiopulmonary diseases), or are not taking medications for these diseases

3) have not engaged in weight training over the past 12 months; this means less than one training session per every two weeks

4) have not engaged in lower-body endurance training over the past 12 months; this means that the participant has not run more than 10 miles per week or road-biked/swam more than 2 days per week

5) have not consumed nutritional supplements two months prior to the study including hormone boosters, protein supplements, amino acid supplements and/or creatine monohydrate

6) are not regular tobacco users, or haven't been over the past 12 months

7) do not have any blood clotting issues and/or are on blood-thinning agents or medications

If you decide to participate in this research study, you will be asked to:

1) Complete a consent and pre-test at the Auburn School of Kinesiology, located at 301 Wire Road, Auburn AL (room 136), lasting 80 min, where you will perform a whole-body x-ray scan, ultrasound scan of the leg, blood draw, and skeletal muscle biopsy from the mid-thigh and fat biopsy from the upper buttock region.
2) Complete visit #2, lasting 60 min, which involves doing a leg strength test (BIODEX), and practice leg exercise session.

The Auburn University Institutional Review Board has approved this Document for use from
05/18/2016 to 08/18/2016
Protocol # 15-320 MR 1508

3) Complete visit #3, lasting 4 hours, where you will perform a whole-body resistance training workout and in the Kinesiology building and donate a 2nd muscle biopsy from the mid-thigh as well as two additional blood draws.
4) Complete visits 4-38 over a 12-week period, lasting 45 min each, where you will perform whole-body resistance training workouts and in the Kinesiology building.
5) Complete a visit #39, lasting 80 min, where you will perform a whole-body x-ray scan, ultrasound scan of the leg, blood draw, a 3rd skeletal muscle biopsy from the mid-thigh, a 2nd fat biopsy from the upper buttock region and BIODEX.

The total approximate time commitment in the Kinesiology building will be approximately 33-36 hours.

You will be monetarily compensated in the form of a $500 check for your time commitment upon completion of the study (partial compensation will be provided for non-completion).

If you are interested in participating in this study, please e-mail your name and contact information to Brooks Mobley at moblecb@auburn.edu or Mike Roberts, PhD at mdr0024@auburn.edu."

The Auburn University Institutional Review Board has approved this Document for use from
05/18/2016 to 08/18/2016
Protocol # 15-320 MR 1508

# Advertisement for a Paid Research Study Titled:

**The Effects of Different Protein and Amino Acid Supplements on Muscle Hypertrophy after 12 Weeks of Resistance Exercise in Untrained Men**

Researchers in The School of Kinesiology at Auburn University are conducting a study which will evaluate which amino acid or protein source supplement (specifically branched chain amino acids, whey, or soy) enhances markers of skeletal muscle growth and body fat reduction when ingested in conjunction with chronic resistance training.

You may qualify if you:

1) are between the ages of 19-35 and have a body mass index (body mass in kilograms/ height in meters^2) between 23-30

2) are apparently healthy and do not have any known illnesses that are contraindicated for exercise (i.e., hypertension, arrhythmias, or cardiopulmonary diseases), or are not taking medications for these diseases

3) have not engaged in weight training over the past 12 months; this means less than one training session per every two weeks

4) have not engaged in lower-body endurance training over the past 12 months; this means that the participant has not run more than 10 miles per week or road-biked/swam more than 2 days per week

5) have not consumed nutritional supplements two months prior to the study including hormone boosters, protein supplements, amino acid supplements and/or creatine monohydrate

6) are not regular tobacco users, or haven’t been over the past 12 months

7) do not have any blood clotting issues and/or are on blood-thinning agents or medications

If you decide to participate in this research study, you will be asked to:

1) Complete a consent and pre-test at the Auburn School of Kinesiology, located at 301 Wire Road, Auburn AL (room 136), lasting 80 min, where you will perform a whole-body x-ray scan, ultrasound scan of the leg, blood draw, and skeletal muscle biopsy from the mid-thigh and fat biopsy from the upper buttock region.
2) Complete visit #2, lasting 60 min, which involves doing a leg strength test (BIODEX), and practice leg exercise session.

The Auburn University Institutional Review Board has approved this Document for use from
__05/18/2016__to__08/18/2016__
Protocol # __15-320 MR 1508___

3) Complete visit #3, lasting 4 hours, where you will perform a whole-body resistance training workout and in the Kinesiology building and donate a 2nd muscle biopsy from the mid-thigh as well as two additional blood draws.
4) Complete visits 4-38 over a 12-week period, lasting 45 min each, where you will perform whole-body resistance training workouts and in the Kinesiology building.
5) Complete a visit #39, lasting 80 min, where you will perform a whole-body x-ray scan, ultrasound scan of the leg, blood draw, a 3rd skeletal muscle biopsy from the mid-thigh, a 2nd fat biopsy from the upper buttock region and BIODEX.

The total approximate time commitment in the Kinesiology building will be approximately 33-36 hours.

You will be monetarily compensated in the form of a $500 check for your time commitment upon completion of the study (partial compensation will be provided for non-completion).

If you are interested in participating in this study, please e-mail your name and contact information to Brooks Mobley at moblecb@auburn.edu or Mike Roberts, PhD at mdr0024@auburn.edu.

The Auburn University Institutional Review Board has approved this Document for use from
__05/18/2016__to__08/18/2016__
Protocol # __15-320 MR 1508___

The Auburn University Institutional Review Board has approved this Document for use from
05/18/2016 to 08/18/2016
Protocol # 15-320 MR 1508

AUBURN UNIVERSITY

COLLEGE OF EDUCATION

**NOTE: DO NOT SIGN THIS DOCUMENT UNLESS AN IRB APPROVAL STAMP WITH CURRENT DATES HAS BEEN APPLIED TO THIS DOCUMENT.**

# Informed consent

**For a Research Study Titled:**

**"The Effects of Different Protein and Amino Acid Supplements on Muscle Hypertrophy after 12 Weeks of Resistance Exercise in Untrained Men"**

**You are invited to participate in a research study** to evaluate the effects of resistance training with 2 doses per day of either: whey protein concentrate, whey protein hydrolysate, soy protein concentrate, branched chain amino acids, or a maltodextrin placebo. The study is being conducted under the direction of the Principal Investigator (PI) Mike Roberts, PhD in the Auburn University School of Kinesiology. You were selected as a possible participant because you are a male that:

1) are between the ages of 19-35 and have a body mass index (body mass in kilograms/ height in meters^2) between 23-30

2) are apparently healthy and do not have any known illnesses that are contraindicated for exercise (i.e., hypertension, arrhythmias, or cardiopulmonary diseases), or are not taking medications for these diseases

3) have not engaged in lower-body weight training over the past 12 months; this means less than one training session per every two weeks

4) have not engaged in lower-body endurance training over the past 12 months; this means that the participant has not run more than 10 miles per week or road-biked/swam more than 2 days per week

5) have not consumed nutritional supplements two months prior to the study including hormone boosters, protein supplements, amino acid supplements and/or creatine monohydrate

6) are not regular tobacco users, or haven't been over the past 12 months

 Participant's initials ______

7) do not have any blood clotting issues and/or are on blood-thinning agents or medications

8) are not allergic to any of the supplements being tested (whey or soy) or are not lactose intolerant

**What will be involved if you participate?** If you decide to participate in this research study, you will be asked to visit the School of Kinesiology on 39 separate occasions (4 longer testing sessions [T1, T2, T3, and T39] and 35 training sessions [T4 through T38] for a total of approximately 33-36 hours).

Below is a schematic of the study timeline, and following the schematic is a detailed paragraph of each testing session:

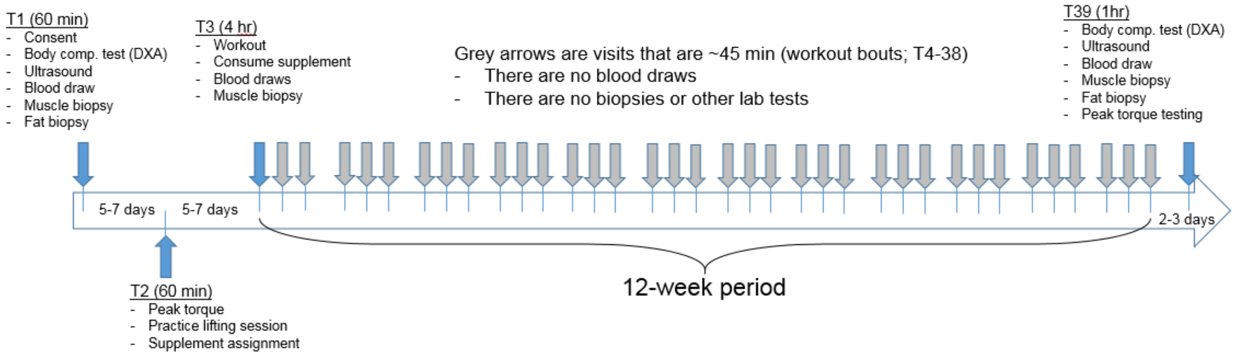


## Visit #1 (T1, 80 min)

You will report to Kinesiology Building room 286 for approximately 1 hour for:

> ***An initial consent, medical questionnaire, and post-consent testing (10 min)***. During this session you will consent to or decline participation. If you consent, you will fill out a brief medical history questionnaire and the PI or a lab member will explain the sequence of testing events for the study. After filling out these forms, you will then perform a series of tests below:
>
> ***Height & weight (5 min).*** You will be asked to remove your shoes and your height and weight will be measured using a standard balance scale.



The Auburn University Institutional Review Board has approved this Document for use from
05/18/2016 to 08/18/2016
Protocol # 15-320 MR 1508

Participant's initials ______

***Urine specific gravity testing (5 min)***. You will then be given a disposable Dixie cup and be instructed to proceed to the restroom and urinate in the cup. You will then leave the cup on the urinal and a lab member will go into the bathroom and retrieve it to analyze the specific gravity using a refractometer. The purpose of this test is to ensure adequate hydration for the body composition testing.

***Dual x-ray absorptiometry (DEXA) body composition testing (10 min)***. You will then be walked downstairs to KINE room 125 and will have your body composition tested using a 'DEXA scanner'. The scan requires you to remain still for 7-10 minutes. During the scan, a low-dose x-ray beam will pass through the entire body. According to the scientific literature, the total radiation exposure is less than that of an airline flight from California to New York and back.

*Please note these DEXA scans are NOT being conducted for clinical purposes which means they are not designed to assess any medical condition you may have. They are being conducted for research purposes only and are not designed to reveal any existing disease or pathology. If however your scan reveals any unexpected findings given your medical history, it will be reviewed by a physician and he will communicate to you if there is a need to follow up with your preferred physician.*

***Ultrasound assessment for leg muscle thickness (5 min).*** Following the DEXA test, you will be escorted to KINE room 260 for this test. This test will require you to lay down face-up on an athletic training table whereby ultrasound pictures will be taken of the outside portion of your upper thigh (the vastus lateralis muscle). This is done by placing a hand-held ultrasound probe on top of the skin, using a small amount of hypoallergenic transmission gel.

***Blood draw (5 min).*** Following height and weight measurement, you will be asked to sit in a phlebotomy chair and have blood drawn from a vein in the front of your elbow. Approximately 1 teaspoon (6 milliliters) of blood will be collected using sterile supplies and techniques. The site will be cleaned and bandaged following the blood draw and you will be given instructions to minimize bruising or discomfort. In addition, in the extraordinarily rate event that you require medical care following the blood draw, you will be referred to go to the on-campus medical clinic or a clinic in the Auburn area.



The Auburn University Institutional Review Board has approved this Document for use from
05/18/2016 to 08/18/2016
Protocol # 15-320 MR 1508

Participant's initials ______

***Skeletal muscle biopsy (20 min).*** Following the blood draw, a skeletal muscle biopsy procedure will be performed.

In the rare event that you are allergic to Lidocaine or betadine/iodine please immediately tell Dr. Roberts.

The person collecting the biopsy (Dr. Roberts) will explain the procedure once more.

Dr. Roberts will then prepare your left middle/outer thigh for the collection of a muscle specimen. Below outlines the events involved with this procedure:

1. The outer aspect of the left mid-thigh will be shaven with electronic clippers in the main laboratory area of room 241.
2. You will then enter an isolated athletic training room within laboratory 241 and an athletic training table will be wiped down with 10% bleach and absorbent paper. You will then lie face up on the training table.
3. The person collecting the biopsy (Dr. Roberts) will then garb in a clean lab gown and sterile gloves.
4. 4 'dots' will be drawn with a Sharpie marker on you left leg to denote where the injection and incision sites are to be made (this will be referred to as the 'collection field').
5. The shaven portion of the leg will be cleansed with sterile, single-use alcohol pads.
6. 0.5 cc of 2% Lidocaine will then be injected subcutaneously within the collection field to de-sensitize pain receptors using a very small needle. During this time, you may feel a slight 'burning' sensation due to the Lidocaine entering the sub-dermal layer. The drug will be allowed 5 min to take effect.
7. 0.5 cc of 2% Lidocaine will then be injected deeper within the collection field to further de-sensitize pain receptors. The drug will be allowed another 5 min to take effect.
   a. During this time, the collection field and 2 inches beyond the collection field will then be swabbed with betadine solution.
   b. A sterile drape will then be placed atop the leg.



The Auburn University Institutional Review Board has approved this Document for use from 05/18/2016 to 08/18/2016
Protocol # 15-320 MR 1508

8. After the 5-min waiting period as mentioned in #7, a sterile/single-use no. 11 blade will be used to make a 1 cm incision (the width of your pinky nail) in the center of the collection field in order to facilitate the procedure.
a. Any immediate bleeding will be swabbed with sterile gauze, although excessive bleeding in this area of the leg usually does not occur.
b. After use, the blade will be placed in a sharps biohazard container and the soiled gauze pads will be placed in a biohazard container.
9. A sterilized biopsy needle (attached to a 60cc syringe with polyethylene tubing) will then be inserted into the pilot hole and will be pierced through the fascia. Once the needle breaches the fascia, Drs. Roberts will instruct the graduate student holding the syringe to apply suction. Dr. Roberts will then apply an upward and downward motion to the needle which excises ~200-300 mg of muscle tissue (which is roughly the size of a no. 2 pencil eraser). During this aspect of the biopsy, you should not feel sharp pain due to the administered lidocaine and will only feel pressure from the biopsy needle. This portion of the procedure typically lasts 5-7 seconds.
10. The needle will be removed from the leg, large sterile gauze pads will be placed atop the pilot incision, and pressure will be held by the investigator for up to 10 min.
    a. The assisting graduate student will take the needle and remove the muscle tissue for preservation.
    b. The student will then begin sterilizing the biopsy needle using bleach and an autoclave (high pressure, high temperature) oven.
11. Following pressure application on the collection field, the pilot incision will be pinched shut and a butterfly bandage will be applied to keep the pilot incision closed.
12. Triple antibiotic will be applied around the incision site, sterile gauze will be placed atop the butterfly bandage, and a large adhesive bandage will be placed atop the collection site.

Prior to leaving the laboratory, Dr. Roberts will hand you a biopsy care sheet to abide by in order to optimize wound closure from the procedure and ask that you return to the laboratory 24 hours after your testing session so that they can examine the collection sites.



The Auburn University Institutional Review Board has approved this Document for use from
05/18/2016 to 08/18/2016
Protocol # 15-320 MR 1508

***Fat biopsy (20 min).*** Following the skeletal muscle biopsy procedure, a fat biopsy will be performed.

In the rare event that you are allergic to Lidocaine or betadine/iodine please immediately tell Dr. Roberts.

The person collecting the biopsy (Dr. Roberts) will explain the procedure once more.

Dr. Roberts will then prepare the outer aspect of the upper right buttock (i.e., 'love handle'). Below outlines the events involved with this procedure:

1. You will remain on the isolated athletic training room within laboratory 241 after the muscle biopsy. You will then lie face down on the training table.
2. The person collecting the biopsy (Dr. Roberts) will then garb in a clean lab gown and sterile gloves.
3. 4 'dots' will be drawn with a Sharpie marker on your upper buttock to denote where the injection and incision sites are to be made (this will be referred to as the 'collection field').
4. 0.5 cc of 2% Lidocaine will then be injected subcutaneously within the collection field to de-sensitize pain receptors using a very small needle. During this time, you may feel a slight 'burning' sensation due to the Lidocaine entering the sub-dermal layer. The drug will be allowed 5 min to take effect.
5. 0.5 cc of 2% Lidocaine will then be injected deeper within the collection field to further de-sensitize pain receptors. The drug will be allowed another 5 min to take effect.
    a. During this time, the collection field and 2 inches beyond the collection field will then be swabbed with betadine solution.
    b. A sterile drape will then be placed atop collection site.
6. After the 5-min waiting period, a sterile/single-use no. 11 blade will be used to make a 1 cm incision (the width of your pinky nail) in the center of the collection field in order to facilitate the procedure.

a. Any immediate bleeding will be swabbed with sterile gauze, although excessive bleeding in this upper buttock region usually does not occur.



The Auburn University Institutional Review Board has approved this Document for use from
__05/18/2016__to__08/18/2016__
Protocol # __15-320 MR 1508__

   b. After use, the blade will be placed in a sharps biohazard container and the soiled gauze pads will be placed in a biohazard container.
7. A sterilized biopsy needle (attached to a 60cc syringe with polyethylene tubing) will then be inserted into the pilot hole and will be pierced in an area of fat. Once the needle is settled, Drs. Roberts will instruct the graduate student holding the syringe to apply suction. Dr. Roberts will then apply an upward and downward motion to the needle which excises ~200-300 mg of fat tissue (which is roughly the size of a no. 2 pencil eraser). During this aspect of the biopsy, you should not feel sharp pain due to the administered lidocaine and will only feel pressure from the biopsy needle. This portion of the procedure typically lasts 5-7 seconds.
8. The needle will be removed from the collection site, large sterile gauze pads will be placed atop the pilot incision, and pressure will be held by the investigator for up to 10 min.
   a. The assisting graduate student will take the needle and remove the fat tissue for preservation.
   b. The student will then begin sterilizing the biopsy needle using bleach and an autoclave (high pressure, high temperature) oven.
9. Following pressure application on the collection field, the pilot incision will be pinched shut and a butterfly bandage will be applied to keep the pilot incision closed.
10. Triple antibiotic will be applied around the incision site, sterile gauze will be placed atop the butterfly bandage, and a large adhesive bandage will be placed atop the collection site.

Prior to leaving the laboratory, Dr. Roberts will hand you a biopsy care sheet to abide by in order to optimize wound closure from the procedure and ask that you return to the laboratory 24 hours after your testing session so that they can examine the collection sites.

Dr. Roberts or a staff member will also give you a 4-day food log to fill out prior to returning to the laboratory for T2 described below.

**<u>Visit #2 (T2, 60 min)</u>**



The Auburn University Institutional Review Board has approved this Document for use from 05/18/2016 to 08/18/2016
Protocol # 15-320 MR 1508

The Auburn University Institutional Review Board has approved this Document for use from
05/18/2016 to 08/18/2016
Protocol # 15-320 MR 1508

AUBURN UNIVERSITY

COLLEGE OF EDUCATION

5-7 days following T1, you will report to Kinesiology room 286, and return your completed food log. You will then engage in the following tests that will last for approximately 1 hour:

***Isokinetic peak torque testing (15 min)***. You will then perform a knee extension peak isokinetic torque test of the right knee extensors. During this test you will have 1 warm-up set of 10 repetitions. Following the warm-up set, you will kick as forcefully as possible against a fixed speed (60 degrees per second) programmed by the BioDex System 4 Isokinetic dynamometer. This test involves extraordinarily low injury risk and is commonly used in rehabilitation settings in order to get a scientific measurement of leg strength.

***Practice whole-body resistance exercise (40 min)***. You will then perform regular weight lifting exercises using a knee extension device that has resistance loaded with free weight plates. You will perform 1 sets of 3-10 repetitions of squats, bench press, bent-over rows, deadlifts and crunches in order to assess your strength. During this practice lifting session, we will determine your 'optimal' lifting load (or resistance) for which you can successfully lift for 3-5 times. This will serve to familiarize you with the exercise devices (which you'll use to train on for visits 3-38) as well as determine the training load to assign to you during these visits.

***Assignment into a supplement or placebo group (5 min)***. You will then be assigned to one of 5 groups described below and will be given a 12-week supply of your supplement. You will be blinded to the supplement composition. Supplements will include:

1. Placebo (PLA). You will consume one serving of maltodextrin PLA capsules (6 g/serving) on whole body workout training days (*described below which will occur during visits 3-39*) immediately after each whole body workout and up to 1 hr prior to bedtime. On non-training days, you will be encouraged to consume one between meals and one serving up to 1 hr prior to bedtime.

2. Branched-chain amino acids (BCAA). You will consume one serving of BCAA capsules (6 g/serving) on whole body workout training days (*described below which will occur during visits 3-39*) immediately after each whole body workout and up to 1 hr prior to bedtime. On non-training days, you will be

 Participant's initials ______

The Auburn University Institutional Review Board has approved this Document for use from
05/18/2016 to 08/18/2016
Protocol # 15-320 MR 1508



encouraged to consume one between meals and one serving up to 1 hr prior to bedtime.

3. Whey protein concentrate (WPC). You will consume one serving of WPC powder in 16 fl oz of water (40 g/serving) on whole body workout training days (*described below which will occur during visits 3-39*) immediately after each whole body workout and up to 1 hr prior to bedtime. On non-training days, you will be encouraged to consume one between meals and one serving up to 1 hr prior to bedtime.

4. Whey protein hydrolysate (WPH). You will consume one serving of WPH powder in 16 fl oz of water (40 g/serving) on whole body workout training days (*described below which will occur during visits 3-39*) immediately after each whole body workout and up to 1 hr prior to bedtime. On non-training days, you will be encouraged to consume one between meals and one serving up to 1 hr prior to bedtime.

5. Soy protein concentrate (SPC). You will consume one serving of SPC powder in 16 fl oz of water (40 g/serving) on whole body workout training days (*described below which will occur during visits 3-39*) immediately after each whole body workout and up to 1 hr prior to bedtime. On non-training days, you will be encouraged to consume one between meals and one serving up to 1 hr prior to bedtime.

You will then be scheduled for T3 which will occur 5-7 days following T2. We ask that you come at least 4 hours fasted to this session.

**Visit #3 (T3, 4 hr)**

You will report to Kinesiology Building room 286 for approximately 4 hours for:

***Whole-body workout and supplement ingestion post-workout (45 min).*** You will perform a whole-body workout which will consist of 3 sets of 3-10 repetitions of squats, bench press, bent-over rows, deadlifts and crunches (with 2 min of rest between sets). The lifting load (or resistance) will be 80-95% of your maximal effort as assessed previously during T2. The lifting load (or resistance) will be

 Participant's initials ______

The Auburn University Institutional Review Board has approved this Document for use from
05/18/2016 to 08/18/2016
Protocol # 15-320 MR 1508

about 70-80% of your maximal effort which will have been determined from T2. Immediately after the exercise bout, you will ingest the supplement to which you were assigned to during T2.

***Blood draws at 30 min and 60 min post-exercise (5 min).*** Approximately 1 teaspoon (6 milliliters) of blood will be collected using sterile supplies and techniques described above at these two post-exercise time points. This will serve to examine if post-workout/post-supplement ingestion affects circulating serum amino acid levels.

***2-h post-exercise biopsy skeletal muscle biopsy (20 min).*** Following the blood draw, a skeletal muscle biopsy procedure will be performed on the left leg as described above. This will serve to examine if post-workout/post-supplement ingestion affects intramuscular anabolic signaling in an acute fashion after one bout and treatment.

More specifically, these acute blood and muscle responses will be examined to see if acute changes in these tissues are reflective of chronic changes with the 12-week protocol.

**Visit #4-38 (T4 thru 38 which occur over a 12-week period, 45 min each)**

You will report to Kinesiology Building room 136 for approximately 45 minutes for each visit for whole-body resistance training sessions. Typically, you'll come in on either Monday, Wednesday and Friday OR Tues, Thurs, and Saturday. You will perform 3 sets of 3-10 repetitions of squats, bench press, bent-over rows, deadlifts and crunches (with 2 min of rest between sets). The lifting load (or resistance) will be 80-95% of your maximal effort as assessed previously during T2. Following each training session (in the laboratory) we will have you consume one serving of your supplement to which you are assigned to. You will also be instructed to consume one serving of your supplement prior to bedtime. On non-training days, we ask that you consume your supplement once between meals and once prior to bedtime.

This phase of the study will occur over a 12-week period.

**Visit #39 (T39, 80 min)**

You will report to Kinesiology Building room 286 for approximately 1 hour for:

 Participant's initials ______

***Urine specific gravity testing (5 min)***. You will be given a disposable Dixie cup and be instructed to proceed to the restroom and urinate in the cup. You will then leave the cup on the urinal and a lab member will go into the bathroom and retrieve it to analyze the specific gravity using a refractometer. Again, the purpose of this test is to ensure adequate hydration for the body composition testing.

***Dual x-ray absorptiometry (DEXA) body composition testing (10 min)***. You will then be walked downstairs to KINE room 125 and will have your body composition tested using a 'DEXA scanner' as described above during T1.

***Ultrasound assessment for leg muscle thickness (5 min).*** Following the DEXA test, you will be escorted to KINE room 260 for this test as described above during T1.

***Blood draw (5 min).*** Following height and weight measurement, you will be asked to sit in a phlebotomy chair and have blood drawn from a vein in the front of your elbow. Approximately 1 tablespoon (6 milliliters) of blood will be collected as described above during T1.

***Skeletal muscle biopsy (20 min).*** Following the blood draw, a skeletal muscle biopsy procedure will be performed on the left leg as described above during T1.

***Fat biopsy (20 min).*** Following the blood draw, a fat biopsy procedure will be performed on the upper buttock as described above during T1.

***Isokinetic peak torque testing (15 min)***. You will then perform a knee extension peak isokinetic torque test of the right knee extensors. During this test you will have 1 warm-up set of 10 repetitions. Following the warm-up set, you will kick as forcefully as possible against a fixed speed (60 degrees per second) programmed by the BioDex System 4 Isokinetic dynamometer. This test involves extraordinarily low injury risk and is commonly used in rehabilitation settings in order to get a scientific measurement of leg strength.

**Are there any risks or discomforts?** The risks associated with participating in this study are:



The Auburn University Institutional Review Board has approved this Document for use from
05/18/2016 to 08/18/2016
Protocol # 15-320 MR 1508

Participant's initials ______

1) The chance of a hematoma (bruise) formation from the muscle biopsy procedure, fat biopsy procedure or venipuncture.

2) Mild discomfort (i.e., pressure) from the muscle or fat biopsy procedures or venipuncture.

3) There is a rare chance that muscle or fat biopsy can lead to dull pain beyond the day of testing, become infected, or produce non-stop bleeding. To this end, hundreds of studies have been performing this procedure, and many of these studies have been performed on non-medical campuses. Furthermore, Drs. Mike Roberts and Dr. David Pascoe are experts of this procedure, and have performed hundreds using sterile techniques without any complications. It should be also noted that Highstead et al. (J Appl Physiol. 2005 Apr;98(4):1202-6) reported that only 18/1,301 participants experienced a hematoma after collection, only 2/1,301 participants experienced a persistent bleeding/oozing, only 4/1,301 participants experienced pain longer than 3 days after collection, and 0/1,301 participants experienced infection. Hence, these biopsy procedures present minimal risk to the participant when done using sterile technique. Also, please be upfront on the medical history questionnaire about being on blood-thinning medications and verbally let an investigator know that you are on them.

4) Mild discomfort (i.e., muscle soreness) from resistance training. Delayed onset muscle soreness is a likely consequence of exercise training and is not unusual. The muscle soreness is not considered a risk, but a nuisance that resolves itself over time.

5) The BIODEX machine is a form of exercise testing. There is an extraordinarily low risk that an adverse event can occur during exercise testing. To this end, and according to the 2009 ACSM Guidelines for Exercise Testing and Prescription, the risk of death associated with exercise testing is 0.05 percent of 10,000 people in a healthy population.

6) There is the risk that an allergic reaction may occur to lidocaine, betadine or ultrasound gel use. To reduce this risk, we will ask that you are upfront about all allergies in the medical history questionnaires and verbally tell the investigators any allergies that you may have.



The Auburn University Institutional Review Board has approved this Document for use from
05/18/2016 to 08/18/2016
Protocol # 15-320 MR 1508

Participant's initials ______

**Are there any benefits to yourself or others?**

You may increase your strength and muscle mass with training and/or supplementation. You may also enjoy the protein supplements as they are highly palatable.

This study will benefit the scientific community and sports performance practitioners by obtaining data which supports or refutes protein supplementation (with different protein sources) with the intent of building muscle mass and increasing strength.

**Will you receive compensation for participating?** To thank you for your time you will receive $500 upon completion of the study.

Between visits 4 thru 38, if you miss more than 3 whole body workouts (which would equal less than a 90% compliance rate to the protocol) then you will be disqualified from the study.

Partial compensation for the study are as follows:

- $50 for completion of visits 1-2 (1 fat biopsy and 1 muscle biopsy given)
- An additional $100 for completion of visits 1-3 (1 fat biopsy and 2 muscle biopsies given)
- An additional $200 for completion of up to all visits except visit #39 (meaning that all workouts were completed but the last testing session was missed and, thus, the last muscle and fat biopsy was not taken)
- An additional $150 for completion of visit #39

Please be aware that compensation for participation in research may be subject to taxation. If you have any questions regarding taxation, please contact the Office of Procurement and Payment Services (334-844-7771).

**Are there any costs for participating?** If you decide to participate, you will not be monetarily charged for anything.

In the unlikely event that you sustain an injury from participation in this study, the investigators have no current plans to provide funds for any medical expenses or other costs you may incur.



The Auburn University Institutional
Review Board has approved this
Document for use from
05/18/2016 to 08/18/2016
Protocol # 15-320 MR 1508

Participant's initials ______

AUBURN UNIVERSITY

COLLEGE OF EDUCATION

**If you change your mind about participating**, you can withdraw at any time during the study. Your participation is completely voluntary. If you choose to withdraw, your data can be withdrawn as long as it is identifiable. Your decision about whether or not to participate or to stop participating will not jeopardize your future relations with Auburn University or the School of Kinesiology.

**Your privacy will be protected**. Any information obtained in connection with this study will remain confidential. Information obtained through your participation may be published in a professional journal and presented at a professional scientific meeting. However, you will be assigned a participant code upon agreeing to partake in the study and any discussion regarding your data will be associated with your participant code. Only de-identified data will be used to disseminate scientific results, and no one will use this data for commercial use (i.e., marketing).

**If you have questions about this study, please ask them now or contact**:

Mike Roberts, Ph.D. at 785-215-3804 (cell #), 334-844-1925 (office #), or mdr0024@auburn.edu (e-mail)

The Auburn University Institutional Review Board has approved this Document for use from
05/18/2016 to 08/18/2016
Protocol # 15-320 MR 1508

 Participant's initials ______

AUBURN UNIVERSITY

COLLEGE OF EDUCATION

**A copy of this document will be given to you to keep. If a copy is not offered to you, please verbally request a copy from the PI or a lab member.**

**If you have questions about your rights as a research participant**, you may contact the Auburn University Office of Research Compliance or the Institutional Review Board by phone (334)-844-5966 or e-mail at IRBadmin@auburn.edu or IRBChair@auburn.edu.

HAVING READ THE INFORMATION PROVIDED, YOU MUST DECIDE WHETHER OR NOT YOU WISH TO PARTICIPATE IN THIS RESEARCH STUDY. YOUR SIGNATURE INDICATES YOUR WILLINGNESS TO PARTICIPATE.

____________________________________ ____________________________________

Participant's signature Date Investigator obtaining consent Date

____________________________________ ____________________________________

Printed Name Printed Name

For payment purposes please indicate here if you are a:

___________ employee

___________ foreign national

In both situations, taxes will be deducted prior to payment being received.

The Auburn University Institutional Review Board has approved this Document for use from
05/18/2016 to 08/18/2016
Protocol # 15-320 MR 1508

 Participant's initials ______

**Auburn School of Kinesiology**

*Study title:* "The Effects of Different Protein and Amino Acid Supplements on Muscle Hypertrophy after 12 Weeks of Resistance Exercise in Untrained Men"

***Demographics***

Name: ________________________________ Participant ID number: _________

Date: _________________ Age: ____________ Birth Date: _______________

Daytime phone: ________________ Evening contact number:_________________

E-mail: __________________

Preferred method of contact (circle multiple answers):

Daytime contact # Evening contact # E-mail No preference

## ***General Health and History Form***

1. Do you have any cardiovascular or metabolic diseases? Yes No

Please explain and give approximate dates for all “Yes” answers:
______________________________________________________________________________
______________________________________________________________________________
________________________________.

2. Please list any medications you are currently taking and for what conditions:
______________________________________________________________________________
______________________________________________________________________________
________________________________.

3. Are you allergic to local anesthetics (lidocaine, novacaine or similar drugs)?
Yes No

4. Are you allergic to betadine (if yes, tell Dr. Roberts and an alternative anti-bacterial for biopsies can be used)?
Yes No

5. Are you allergic to ultrasound gel?
Yes No

6. Have you had or do you currently have blood clotting issues?
Yes No

Please explain and give dates of “yes” answers:
______________________________________________________________________
_____________________________________________________________

7. Have you consumed supplemental protein, creatine, and/or branched chain amino acids, or agents that affect hormones (testosterone boosters, growth hormone boosters, etc.) within the past 2 months?
Yes No

Please explain and give dates of “yes” answers:
______________________________________________________________________
_____________________________________________________________

8. Have you been a regular tobacco user within the past 12 months (cigarettes, dip, gum, etc.)?
Yes No

Please explain and give dates of “yes” answers:
______________________________________________________________________________
__________________________________________________

9. Are you allergic to any of the supplements being tested (egg, whey, casein, or soy) or are you lactose intolerant?
Yes No

10. Do you have chronic lower back pain and/or have you had back surgery which may hamper your ability to perform weight-training exercises??

Yes No

Please explain and give dates of “yes” answers:

_______________________________________________________________________________

__________________________________________________

11. Beyond the potential risks listed in the IRB form, do you have any reason to believe that your participation in this study may put your health at risk?

Yes No

Please explain and give dates of “yes” answers:

_______________________________________________________________________________

__________________________________________________

**<u>Exercise history (we need this to classify our participants as ‘regularly-active’ or ‘relatively sedentary’)</u>**

Do you regularly exercise train?
If yes, then (on average) how many days per week do you do this?

- Road bike or spin class: _______ days/week, _______ hours/day
- Jog/run or walk: _______ days/week, _______ hours/day
- Road Bike or spin class: _______ days/week, _______ hours/day
- Swim: _______ days/week, _______ hours/day
- Lower-body weight train _______ days/week, _______ hours/day
- Other ____________________________________:
  _______ days/week, _______ hours/day

Please place an 'X' by any of these medications that you are currently or have been taking over the past year.

| Drug Names | Other Names |
|---|---|
| ____ Advate | antihemophilic factor (factor VIII, recombinant) |
| ____ Alphanate | antihemophilic factor (factor VIII, human)/ von Willebrand factor complex (human) |
| ____ Alprolix | coagulation factor IX (recombinant), Fc fusion protein |
| ____ Alteplase | Actilyse or Actilyse 20 |
| ____ Alteplase | Indeparin (5000 iu) or Inderparin |
| ____ Aspirin | |
| | |
| ____ Bebulin VH | coagulation factor IX complex |
| ____ BeneFIX | coagulation factor IX (recombinant) |
| ____ Corifact | factor XIII concentrate (human) |
| ____ Coumadin | Uniwafarin, Sofarin, Lycobion, Sofarin, Uniwafarin, warfarin, warfarin sodium, Warf-1, Warf-2, Warf-5 |
| | |
| ____ Dalteparin | Fragmin or Fragmin PF |
| ____ Danaparoid | Generic |
| ____ Enoxaparin | Clexane, Cutenox, Dyanalix, Enoxarin, Enqxarin, Flothin, Leeparin, Lmwx-PFS |
| ____ Feiba NF | anti-inhibitor coagulant complex |
| ____ Feiba VH | anti-inhibitor coagulant complex |
| ____ Fondaparinux | Arixtra |
| ____ Helixate FS | antihemophilic factor (factor VIII, recombinant) |
| ____ Hemofil M | antihemophilic factor (factor VIII, human) |
| ____ Humate-P | antihemophilic factor (factor VIII, human)/ von Willebrand factor complex (human) |
| ____ Koate-DVI | antihemophilic factor (factor VIII, human) |
| ____ Kogenate FS | antihemophilic factor (factor VIII, recombinant) |
| ____ Lepirudin | generic |
| ____ Mephyton | phytonadione |
| ____ Monarc-M | antihemophilic factor (factor VIII, human) |
| ____ Monoclate-P | antihemophilic factor (factor VIII, human) |
| ____ Mononine | coagulation factor IX (human) |
| ____ NovoSeven RT | coagulation factor VIIa (recombinant) |
| ____ phytonadione | generic |
| ____ Profilnine SD | coagulation factor IX complex |
| ____ Recombinate | antihemophilic factor (factor VIII, recombinant) |
| ____ ReFacto | antihemophilic factor (factor VIII, recombinant) |
| ____ Rixubis | coagulation factor IX (recombinant) |
| ____ Tretten | coagulation factor XIII A-subunit (recombinant) |
| ____ Urokinase | Dabunase, Dukinase, Kd-Unase, Medinase, Solokinase, Uni-kinase, Uroken |
| ____ vitamin K | Phytonadione |

| | |
|---|---|
| ____ Wilate | antihemophilic factor (factor VIII, human)/ von Willebrand factor complex (human) |
| ____ Xyntha | antihemophilic factor (factor VIII, recombinant |

**Please Sign:**

I hereby state that, to the best of my knowledge, my answers to the above questions are correct.

Given that this document contains sensitive personal health information it will be retained behind two locked doors in the office of the Principal Investigator (PI, Michael Roberts, PhD) and will only be accessed by the PI or involved study personnel.

Finally, data collected on these forms will not be disclosed to a third party.

The sole use of this form is to further screen you for study inclusion/exclusion criteria. If there is further question as to your ability to participate in this study, the PI will consult with Dr. Trent Wilson and/or Dr. Michael Goodlett, MD. In this situation, the PI will discuss the case using your participant number and not your name. One year after the study concludes this document will be destroyed.

Subject's Signature: ________________________
Date: _______________

Investigator's Signature: ________________________
Date: _______________

# Auburn School of Kinesiology

*Study title:* **“**The Effects of Different Protein and Amino Acid Supplements on Muscle Hypertrophy after 12 Weeks of Resistance Exercise in Untrained Men”

***Data Collection Form***

---

Participant ID number: _________

**T1; date (time): ____________(_________)**

Informed consent form completed; tester initial if completed: _________

Body weight and height obtained; tester initial if completed: _________

Weight: _______ lbs; Height: _______ inches

Urine specific gravity _________

DXA dual-leg lean mass: ________lb

DXA % fat (total body): ________%

Ultrasound; tester initial if completed: _________

Blood collection; tester initial if completed: _________

Skeletal muscle biopsy left leg; tester initial if completed: _________

Fat biopsy; tester initial if completed: _________

*Give 4-day food log; participant returns in 5-7 days for T2

**T2; date (time): ____________(_________)**

Peak isokinetic torque test of the right leg; tester initial if completed _________

Peak torque at 60: ______________ ft-lbs

Peak torque at 120: ______________ ft-lbs

*Practice exercise session*

Squat weight that can be lifted 3-5 times until fatigue: ________ lb

Bench press weight that can be lifted 3-5 times until fatigue: ________ lb

Bent-over row weight that can be lifted 3-5 times until fatigue: ________ lb

Supplement assignment (A, B, C, D, E): _________

*Give supplements and supplement instructions.

**T3; date (time): ____________(_________)**

Whole-body workout
(4 sets of 10 reps is the goal, 2 min between sets)

Squat
Set 1: ______ lbs x _______ reps
Set 2: ______ lbs x _______ reps
Set 3: ______ lbs x _______ reps

Bench press
Set 1: ______ lbs x _______ reps
Set 2: ______ lbs x _______ reps
Set 3: ______ lbs x _______ reps

Deadlift
Set 1: ______ lbs x _______ reps
Set 2: ______ lbs x _______ reps
Set 3: ______ lbs x _______ reps

Row
Set 1: ______ lbs x _______ reps
Set 2: ______ lbs x _______ reps
Set 3: ______ lbs x _______ reps
*cruches

*ingest supplement immediately post-workout

30 min post blood collection; tester initial if completed: _________

60 min post blood collection; tester initial if completed: _________

2 hr post biopsy left leg; tester initial if completed: _________

**T4; date (time): ____________(_________)**

Whole-body workout
(3 sets of 10 reps is the goal, 2 min between sets)

Squat
Set 1: ______ lbs x _______ reps
Set 2: ______ lbs x _______ reps
Set 3: ______ lbs x _______ reps

Bench press
Set 1: ______ lbs x _______ reps
Set 2: ______ lbs x _______ reps
Set 3: ______ lbs x _______ reps

Deadlift
Set 1: ______ lbs x _______ reps
Set 2: ______ lbs x _______ reps
Set 3: ______ lbs x _______ reps

Row
Set 1: ______ lbs x _______ reps
Set 2: ______ lbs x _______ reps
Set 3: ______ lbs x _______ reps
*crunches

**T6; date (time): ____________(_________)**
Whole-body workout
(3 sets of 3 reps is the goal, 2 min between sets)

Squat
Set 1: ______ lbs x _______ reps
Set 2: ______ lbs x _______ reps
Set 3: ______ lbs x _______ reps

Bench press
Set 1: ______ lbs x _______ reps
Set 2: ______ lbs x _______ reps
Set 3: ______ lbs x _______ reps

Deadlift
Set 1: ______ lbs x _______ reps
Set 2: ______ lbs x _______ reps
Set 3: ______ lbs x _______ reps

Row
Set 1: ______ lbs x _______ reps
Set 2: ______ lbs x _______ reps
Set 3: ______ lbs x _______ reps
*crunches

**T5; date (time): ____________(_________)**

Whole-body workout
(3 sets of 6 reps is the goal, 2 min between sets)

Squat
Set 1: ______ lbs x _______ reps
Set 2: ______ lbs x _______ reps
Set 3: ______ lbs x _______ reps

Bench press
Set 1: ______ lbs x _______ reps
Set 2: ______ lbs x _______ reps
Set 3: ______ lbs x _______ reps

Deadlift
Set 1: ______ lbs x _______ reps
Set 2: ______ lbs x _______ reps
Set 3: ______ lbs x _______ reps

Row
Set 1: ______ lbs x _______ reps
Set 2: ______ lbs x _______ reps
Set 3: ______ lbs x _______ reps
*crunches

**T7; date (time): ____________(_________)**
Whole-body workout
(3 sets of 10 reps is the goal, 2 min between sets)

Squat
Set 1: ______ lbs x _______ reps
Set 2: ______ lbs x _______ reps
Set 3: ______ lbs x _______ reps

Bench press
Set 1: ______ lbs x _______ reps
Set 2: ______ lbs x _______ reps
Set 3: ______ lbs x _______ reps

Deadlift
Set 1: ______ lbs x _______ reps
Set 2: ______ lbs x _______ reps
Set 3: ______ lbs x _______ reps

Row
Set 1: ______ lbs x _______ reps
Set 2: ______ lbs x _______ reps
Set 3: ______ lbs x _______ reps
*crunches

**T8; date (time): ____________(_________)**

Whole-body workout
(3 sets of 6 reps is the goal, 2 min between sets)

Squat
Set 1: ______ lbs x _______ reps
Set 2: ______ lbs x _______ reps
Set 3: ______ lbs x _______ reps

Bench press
Set 1: ______ lbs x _______ reps
Set 2: ______ lbs x _______ reps
Set 3: ______ lbs x _______ reps

Deadlift
Set 1: ______ lbs x _______ reps
Set 2: ______ lbs x _______ reps
Set 3: ______ lbs x _______ reps

Row
Set 1: ______ lbs x _______ reps
Set 2: ______ lbs x _______ reps
Set 3: ______ lbs x _______ reps
*crunches

**T10; date (time): ____________(_________)**
Whole-body workout
(3 sets of 10 reps is the goal, 2 min between sets)

Squat
Set 1: ______ lbs x _______ reps
Set 2: ______ lbs x _______ reps
Set 3: ______ lbs x _______ reps

Bench press
Set 1: ______ lbs x _______ reps
Set 2: ______ lbs x _______ reps
Set 3: ______ lbs x _______ reps

Deadlift
Set 1: ______ lbs x _______ reps
Set 2: ______ lbs x _______ reps
Set 3: ______ lbs x _______ reps

Row
Set 1: ______ lbs x _______ reps
Set 2: ______ lbs x _______ reps
Set 3: ______ lbs x _______ reps
*crunches

**T9; date (time): ____________(_________)**

Whole-body workout
(3 sets of 3 reps is the goal, 2 min between sets)

Squat
Set 1: ______ lbs x _______ reps
Set 2: ______ lbs x _______ reps
Set 3: ______ lbs x _______ reps

Bench press
Set 1: ______ lbs x _______ reps
Set 2: ______ lbs x _______ reps
Set 3: ______ lbs x _______ reps

Deadlift
Set 1: ______ lbs x _______ reps
Set 2: ______ lbs x _______ reps
Set 3: ______ lbs x _______ reps

Row
Set 1: ______ lbs x _______ reps
Set 2: ______ lbs x _______ reps
Set 3: ______ lbs x _______ reps
*crunches

**T11; date (time): ____________(_________)**
Whole-body workout
(3 sets of 6 reps is the goal, 2 min between sets)

Squat
Set 1: ______ lbs x _______ reps
Set 2: ______ lbs x _______ reps
Set 3: ______ lbs x _______ reps

Bench press
Set 1: ______ lbs x _______ reps
Set 2: ______ lbs x _______ reps
Set 3: ______ lbs x _______ reps

Deadlift
Set 1: ______ lbs x _______ reps
Set 2: ______ lbs x _______ reps
Set 3: ______ lbs x _______ reps

Row
Set 1: ______ lbs x _______ reps
Set 2: ______ lbs x _______ reps
Set 3: ______ lbs x _______ reps
*crunches

**T12; date (time): ____________(_________)**

Whole-body workout
(3 sets of 3 reps is the goal, 2 min between sets)

Squat
Set 1: ______ lbs x _______ reps
Set 2: ______ lbs x _______ reps
Set 3: ______ lbs x _______ reps

Bench press
Set 1: ______ lbs x _______ reps
Set 2: ______ lbs x _______ reps
Set 3: ______ lbs x _______ reps

Deadlift
Set 1: ______ lbs x _______ reps
Set 2: ______ lbs x _______ reps
Set 3: ______ lbs x _______ reps

Row
Set 1: ______ lbs x _______ reps
Set 2: ______ lbs x _______ reps
Set 3: ______ lbs x _______ reps
*crunches

**T14; date (time): ____________(_________)**

Whole-body workout
(3 sets of 6 reps is the goal, 2 min between sets)

Squat
Set 1: ______ lbs x _______ reps
Set 2: ______ lbs x _______ reps
Set 3: ______ lbs x _______ reps

Bench press
Set 1: ______ lbs x _______ reps
Set 2: ______ lbs x _______ reps
Set 3: ______ lbs x _______ reps

Deadlift
Set 1: ______ lbs x _______ reps
Set 2: ______ lbs x _______ reps
Set 3: ______ lbs x _______ reps

Row
Set 1: ______ lbs x _______ reps
Set 2: ______ lbs x _______ reps
Set 3: ______ lbs x _______ reps
*crunches

**T13; date (time): ____________(_________)**

Whole-body workout
(3 sets of 10 reps is the goal, 2 min between sets)

Squat
Set 1: ______ lbs x _______ reps
Set 2: ______ lbs x _______ reps
Set 3: ______ lbs x _______ reps

Bench press
Set 1: ______ lbs x _______ reps
Set 2: ______ lbs x _______ reps
Set 3: ______ lbs x _______ reps

Deadlift
Set 1: ______ lbs x _______ reps
Set 2: ______ lbs x _______ reps
Set 3: ______ lbs x _______ reps

Row
Set 1: ______ lbs x _______ reps
Set 2: ______ lbs x _______ reps
Set 3: ______ lbs x _______ reps
*crunches

**T15; date (time): ____________(_________)**

Whole-body workout
(3 sets of 3 reps is the goal, 2 min between sets)

Squat
Set 1: ______ lbs x _______ reps
Set 2: ______ lbs x _______ reps
Set 3: ______ lbs x _______ reps

Bench press
Set 1: ______ lbs x _______ reps
Set 2: ______ lbs x _______ reps
Set 3: ______ lbs x _______ reps

Deadlift
Set 1: ______ lbs x _______ reps
Set 2: ______ lbs x _______ reps
Set 3: ______ lbs x _______ reps

Row
Set 1: ______ lbs x _______ reps
Set 2: ______ lbs x _______ reps
Set 3: ______ lbs x _______ reps
*crunches

**T16; date (time): ____________(_________)**

Whole-body workout
(3 sets of 10 reps is the goal, 2 min between sets)

Squat
Set 1: ______ lbs x _______ reps
Set 2: ______ lbs x _______ reps
Set 3: ______ lbs x _______ reps

Bench press
Set 1: ______ lbs x _______ reps
Set 2: ______ lbs x _______ reps
Set 3: ______ lbs x _______ reps

Deadlift
Set 1: ______ lbs x _______ reps
Set 2: ______ lbs x _______ reps
Set 3: ______ lbs x _______ reps

Row
Set 1: ______ lbs x _______ reps
Set 2: ______ lbs x _______ reps
Set 3: ______ lbs x _______ reps
*crunches

**T18; date (time): ____________(_________)**
Whole-body workout
(3 sets of 3 reps is the goal, 2 min between sets)

Squat
Set 1: ______ lbs x _______ reps
Set 2: ______ lbs x _______ reps
Set 3: ______ lbs x _______ reps

Bench press
Set 1: ______ lbs x _______ reps
Set 2: ______ lbs x _______ reps
Set 3: ______ lbs x _______ reps

Deadlift
Set 1: ______ lbs x _______ reps
Set 2: ______ lbs x _______ reps
Set 3: ______ lbs x _______ reps

Row
Set 1: ______ lbs x _______ reps
Set 2: ______ lbs x _______ reps
Set 3: ______ lbs x _______ reps
*crunches

**T17; date (time): ____________(_________)**

Whole-body workout
(3 sets of 6 reps is the goal, 2 min between sets)

Squat
Set 1: ______ lbs x _______ reps
Set 2: ______ lbs x _______ reps
Set 3: ______ lbs x _______ reps

Bench press
Set 1: ______ lbs x _______ reps
Set 2: ______ lbs x _______ reps
Set 3: ______ lbs x _______ reps

Deadlift
Set 1: ______ lbs x _______ reps
Set 2: ______ lbs x _______ reps
Set 3: ______ lbs x _______ reps

Row
Set 1: ______ lbs x _______ reps
Set 2: ______ lbs x _______ reps
Set 3: ______ lbs x _______ reps
*crunches

**T19; date (time): ____________(_________)**
Whole-body workout
(3 sets of 10 reps is the goal, 2 min between sets)

Squat
Set 1: ______ lbs x _______ reps
Set 2: ______ lbs x _______ reps
Set 3: ______ lbs x _______ reps

Bench press
Set 1: ______ lbs x _______ reps
Set 2: ______ lbs x _______ reps
Set 3: ______ lbs x _______ reps

Deadlift
Set 1: ______ lbs x _______ reps
Set 2: ______ lbs x _______ reps
Set 3: ______ lbs x _______ reps

Row
Set 1: ______ lbs x _______ reps
Set 2: ______ lbs x _______ reps
Set 3: ______ lbs x _______ reps
*crunches

*****give 3-day food log to complete**

**T20; date (time): _____________(__________)**

Whole-body workout
(3 sets of 6 reps is the goal, 2 min between sets)

Squat
Set 1: ______ lbs x _______ reps
Set 2: ______ lbs x _______ reps
Set 3: ______ lbs x _______ reps

Bench press
Set 1: ______ lbs x _______ reps
Set 2: ______ lbs x _______ reps
Set 3: ______ lbs x _______ reps

Deadlift
Set 1: ______ lbs x _______ reps
Set 2: ______ lbs x _______ reps
Set 3: ______ lbs x _______ reps

Row
Set 1: ______ lbs x _______ reps
Set 2: ______ lbs x _______ reps
Set 3: ______ lbs x _______ reps
*crunches

**T22; date (time): _____________(__________)**
Whole-body workout
(3 sets of 10 reps is the goal, 2 min between sets)

Squat
Set 1: ______ lbs x _______ reps
Set 2: ______ lbs x _______ reps
Set 3: ______ lbs x _______ reps

Bench press
Set 1: ______ lbs x _______ reps
Set 2: ______ lbs x _______ reps
Set 3: ______ lbs x _______ reps

Deadlift
Set 1: ______ lbs x _______ reps
Set 2: ______ lbs x _______ reps
Set 3: ______ lbs x _______ reps

Row
Set 1: ______ lbs x _______ reps
Set 2: ______ lbs x _______ reps
Set 3: ______ lbs x _______ reps
*crunches

**T21; date (time): _____________(__________)**

Whole-body workout
(3 sets of 3 reps is the goal, 2 min between sets)

Squat
Set 1: ______ lbs x _______ reps
Set 2: ______ lbs x _______ reps
Set 3: ______ lbs x _______ reps

Bench press
Set 1: ______ lbs x _______ reps
Set 2: ______ lbs x _______ reps
Set 3: ______ lbs x _______ reps

Deadlift
Set 1: ______ lbs x _______ reps
Set 2: ______ lbs x _______ reps
Set 3: ______ lbs x _______ reps

Row
Set 1: ______ lbs x _______ reps
Set 2: ______ lbs x _______ reps
Set 3: ______ lbs x _______ reps
*crunches

**T23; date (time): _____________(__________)**
Whole-body workout
(3 sets of 6 reps is the goal, 2 min between sets)

Squat
Set 1: ______ lbs x _______ reps
Set 2: ______ lbs x _______ reps
Set 3: ______ lbs x _______ reps

Bench press
Set 1: ______ lbs x _______ reps
Set 2: ______ lbs x _______ reps
Set 3: ______ lbs x _______ reps

Deadlift
Set 1: ______ lbs x _______ reps
Set 2: ______ lbs x _______ reps
Set 3: ______ lbs x _______ reps

Row
Set 1: ______ lbs x _______ reps
Set 2: ______ lbs x _______ reps
Set 3: ______ lbs x _______ reps
*crunches

**T24; date (time): ____________(_________)**

Whole-body workout
(3 sets of 3 reps is the goal, 2 min between sets)

Squat
Set 1: ______ lbs x _______ reps
Set 2: ______ lbs x _______ reps
Set 3: ______ lbs x _______ reps

Bench press
Set 1: ______ lbs x _______ reps
Set 2: ______ lbs x _______ reps
Set 3: ______ lbs x _______ reps

Deadlift
Set 1: ______ lbs x _______ reps
Set 2: ______ lbs x _______ reps
Set 3: ______ lbs x _______ reps

Row
Set 1: ______ lbs x _______ reps
Set 2: ______ lbs x _______ reps
Set 3: ______ lbs x _______ reps
*crunches

**T26; date (time): ____________(_________)**
Whole-body workout
(3 sets of 6 reps is the goal, 2 min between sets)

Squat
Set 1: ______ lbs x _______ reps
Set 2: ______ lbs x _______ reps
Set 3: ______ lbs x _______ reps

Bench press
Set 1: ______ lbs x _______ reps
Set 2: ______ lbs x _______ reps
Set 3: ______ lbs x _______ reps

Deadlift
Set 1: ______ lbs x _______ reps
Set 2: ______ lbs x _______ reps
Set 3: ______ lbs x _______ reps

Row
Set 1: ______ lbs x _______ reps
Set 2: ______ lbs x _______ reps
Set 3: ______ lbs x _______ reps
*crunches

**T25; date (time): ____________(_________)**

Whole-body workout
(3 sets of 10 reps is the goal, 2 min between sets)

Squat
Set 1: ______ lbs x _______ reps
Set 2: ______ lbs x _______ reps
Set 3: ______ lbs x _______ reps

Bench press
Set 1: ______ lbs x _______ reps
Set 2: ______ lbs x _______ reps
Set 3: ______ lbs x _______ reps

Deadlift
Set 1: ______ lbs x _______ reps
Set 2: ______ lbs x _______ reps
Set 3: ______ lbs x _______ reps

Row
Set 1: ______ lbs x _______ reps
Set 2: ______ lbs x _______ reps
Set 3: ______ lbs x _______ reps
*crunches

**T27; date (time): ____________(_________)**
Whole-body workout
(3 sets of 3 reps is the goal, 2 min between sets)

Squat
Set 1: ______ lbs x _______ reps
Set 2: ______ lbs x _______ reps
Set 3: ______ lbs x _______ reps

Bench press
Set 1: ______ lbs x _______ reps
Set 2: ______ lbs x _______ reps
Set 3: ______ lbs x _______ reps

Deadlift
Set 1: ______ lbs x _______ reps
Set 2: ______ lbs x _______ reps
Set 3: ______ lbs x _______ reps

Row
Set 1: ______ lbs x _______ reps
Set 2: ______ lbs x _______ reps
Set 3: ______ lbs x _______ reps
*crunches

**T28; date (time): ____________(_________)**

Whole-body workout
(3 sets of 10 reps is the goal, 2 min between sets)

Squat
Set 1: ______ lbs x _______ reps
Set 2: ______ lbs x _______ reps
Set 3: ______ lbs x _______ reps

Bench press
Set 1: ______ lbs x _______ reps
Set 2: ______ lbs x _______ reps
Set 3: ______ lbs x _______ reps

Deadlift
Set 1: ______ lbs x _______ reps
Set 2: ______ lbs x _______ reps
Set 3: ______ lbs x _______ reps

Row
Set 1: ______ lbs x _______ reps
Set 2: ______ lbs x _______ reps
Set 3: ______ lbs x _______ reps
*crunches

**T30; date (time): ____________(_________)**
Whole-body workout
(3 sets of 3 reps is the goal, 2 min between sets)

Squat
Set 1: ______ lbs x _______ reps
Set 2: ______ lbs x _______ reps
Set 3: ______ lbs x _______ reps

Bench press
Set 1: ______ lbs x _______ reps
Set 2: ______ lbs x _______ reps
Set 3: ______ lbs x _______ reps

Deadlift
Set 1: ______ lbs x _______ reps
Set 2: ______ lbs x _______ reps
Set 3: ______ lbs x _______ reps

Row
Set 1: ______ lbs x _______ reps
Set 2: ______ lbs x _______ reps
Set 3: ______ lbs x _______ reps
*crunches

**T29; date (time): ____________(_________)**

Whole-body workout
(3 sets of 6 reps is the goal, 2 min between sets)

Squat
Set 1: ______ lbs x _______ reps
Set 2: ______ lbs x _______ reps
Set 3: ______ lbs x _______ reps

Bench press
Set 1: ______ lbs x _______ reps
Set 2: ______ lbs x _______ reps
Set 3: ______ lbs x _______ reps

Deadlift
Set 1: ______ lbs x _______ reps
Set 2: ______ lbs x _______ reps
Set 3: ______ lbs x _______ reps

Row
Set 1: ______ lbs x _______ reps
Set 2: ______ lbs x _______ reps
Set 3: ______ lbs x _______ reps
*crunches

**T31; date (time): ____________(_________)**
Whole-body workout
(3 sets of 10 reps is the goal, 2 min between sets)

Squat
Set 1: ______ lbs x _______ reps
Set 2: ______ lbs x _______ reps
Set 3: ______ lbs x _______ reps

Bench press
Set 1: ______ lbs x _______ reps
Set 2: ______ lbs x _______ reps
Set 3: ______ lbs x _______ reps

Deadlift
Set 1: ______ lbs x _______ reps
Set 2: ______ lbs x _______ reps
Set 3: ______ lbs x _______ reps

Row
Set 1: ______ lbs x _______ reps
Set 2: ______ lbs x _______ reps
Set 3: ______ lbs x _______ reps
*crunches

**T32; date (time): ____________(_________)**

Whole-body workout
(3 sets of 6 reps is the goal, 2 min between sets)

Squat
Set 1: ______ lbs x _______ reps
Set 2: ______ lbs x _______ reps
Set 3: ______ lbs x _______ reps

Bench press
Set 1: ______ lbs x _______ reps
Set 2: ______ lbs x _______ reps
Set 3: ______ lbs x _______ reps

Deadlift
Set 1: ______ lbs x _______ reps
Set 2: ______ lbs x _______ reps
Set 3: ______ lbs x _______ reps

Row
Set 1: ______ lbs x _______ reps
Set 2: ______ lbs x _______ reps
Set 3: ______ lbs x _______ reps
*crunches

**T34; date (time): ____________(_________)**
Whole-body workout
(3 sets of 10 reps is the goal, 2 min between sets)

Squat
Set 1: ______ lbs x _______ reps
Set 2: ______ lbs x _______ reps
Set 3: ______ lbs x _______ reps

Bench press
Set 1: ______ lbs x _______ reps
Set 2: ______ lbs x _______ reps
Set 3: ______ lbs x _______ reps

Deadlift
Set 1: ______ lbs x _______ reps
Set 2: ______ lbs x _______ reps
Set 3: ______ lbs x _______ reps

Row
Set 1: ______ lbs x _______ reps
Set 2: ______ lbs x _______ reps
Set 3: ______ lbs x _______ reps
*crunches

**T33; date (time): ____________(_________)**

Whole-body workout
(3 sets of 3 reps is the goal, 2 min between sets)

Squat
Set 1: ______ lbs x _______ reps
Set 2: ______ lbs x _______ reps
Set 3: ______ lbs x _______ reps

Bench press
Set 1: ______ lbs x _______ reps
Set 2: ______ lbs x _______ reps
Set 3: ______ lbs x _______ reps

Deadlift
Set 1: ______ lbs x _______ reps
Set 2: ______ lbs x _______ reps
Set 3: ______ lbs x _______ reps

Row
Set 1: ______ lbs x _______ reps
Set 2: ______ lbs x _______ reps
Set 3: ______ lbs x _______ reps
*crunches

**T35; date (time): ____________(_________)**
Whole-body workout
(3 sets of 6 reps is the goal, 2 min between sets)

Squat
Set 1: ______ lbs x _______ reps
Set 2: ______ lbs x _______ reps
Set 3: ______ lbs x _______ reps

Bench press
Set 1: ______ lbs x _______ reps
Set 2: ______ lbs x _______ reps
Set 3: ______ lbs x _______ reps

Deadlift
Set 1: ______ lbs x _______ reps
Set 2: ______ lbs x _______ reps
Set 3: ______ lbs x _______ reps

Row
Set 1: ______ lbs x _______ reps
Set 2: ______ lbs x _______ reps
Set 3: ______ lbs x _______ reps
*crunches

**T36; date (time): ____________(_________)**

Whole-body workout
(3 sets of 3 reps is the goal, 2 min between sets)

Squat
Set 1: ______ lbs x _______ reps
Set 2: ______ lbs x _______ reps
Set 3: ______ lbs x _______ reps

Bench press
Set 1: ______ lbs x _______ reps
Set 2: ______ lbs x _______ reps
Set 3: ______ lbs x _______ reps

Deadlift
Set 1: ______ lbs x _______ reps
Set 2: ______ lbs x _______ reps
Set 3: ______ lbs x _______ reps

Row
Set 1: ______ lbs x _______ reps
Set 2: ______ lbs x _______ reps
Set 3: ______ lbs x _______ reps
*crunches

**T38; date (time): ____________(_________)**

Whole-body workout
(3 sets of 6 reps is the goal, 2 min between sets)

Squat
Set 1: ______ lbs x _______ reps
Set 2: ______ lbs x _______ reps
Set 3: ______ lbs x _______ reps

Bench press
Set 1: ______ lbs x _______ reps
Set 2: ______ lbs x _______ reps
Set 3: ______ lbs x _______ reps

Deadlift
Set 1: ______ lbs x _______ reps
Set 2: ______ lbs x _______ reps
Set 3: ______ lbs x _______ reps

Row
Set 1: ______ lbs x _______ reps
Set 2: ______ lbs x _______ reps
Set 3: ______ lbs x _______ reps
*crunches

**T37; date (time): ____________(_________)**

Whole-body workout
(3 sets of 10 reps is the goal, 2 min between sets)

Squat
Set 1: ______ lbs x _______ reps
Set 2: ______ lbs x _______ reps
Set 3: ______ lbs x _______ reps

Bench press
Set 1: ______ lbs x _______ reps
Set 2: ______ lbs x _______ reps
Set 3: ______ lbs x _______ reps

Deadlift
Set 1: ______ lbs x _______ reps
Set 2: ______ lbs x _______ reps
Set 3: ______ lbs x _______ reps

Row
Set 1: ______ lbs x _______ reps
Set 2: ______ lbs x _______ reps
Set 3: ______ lbs x _______ reps
*crunches

*****give 3-day food log to complete**

**T39; date (time): ____________(_________)**

Body weight obtained; tester initial if completed: _________

Weight: _______ lbs;

Urine specific gravity _________

DXA dual-leg lean mass: ________lb

DXA % fat (total body): ________%

Ultrasound; tester initial if completed: _________

Blood collection; tester initial if completed: _________

Skeletal muscle biopsy left leg; tester initial if completed: _________

Fat biopsy; tester initial if completed: _________

Peak isokinetic torque test of the right leg; tester initial if completed _________

Peak torque at 60: ______________ ft-lbs

Peak torque at 120: ______________ ft-lbs

**Memo for:**
**IRB protocol # 15-320 MR 1508 (modification request)**
**"The Effects of Different Protein and Amino Acid Supplements on Muscle Hypertrophy after 12 Weeks of Resistance Exercise in Untrained Men"**

Dear IRB Administration,

As usual, we really appreciate the time and effort put into this review. The lab and students also consistently comment 'how lucky we are to be at Auburn where we can do cool human research'. This is very important for three reasons:

1) I believe the aforementioned sentiment is chiefly due to you all facilitating research rather than being a hindrance so this is much appreciated by myself and the students.
2) The human research approved by you all is giving all of the PhD student GREAT career training and making them competitive for post-Auburn positions (i.e., professorships or post-docs).
3) The human research the is being published is garnering great respect in the field of sports nutrition and Auburn Kinesiology is beginning to be viewed as a hub of quality research in this area from other academic institutions and industry sponsors.

With regard to the current protocol, we have addressed each area of concern in a point-by point fashion.

If we have not adequately provided details needed, do not hesitate to contact me.

<u>Finally, it should be noted that the first set of files in this document are all highlighted with changes made. These pages are followed by clean versions of these documents for stamping/approval purposes.</u>

Thank you again and I look forward to our future correspondence.

Warm regards,

Mike Roberts, PhD

---

<u>The AU IRB's comments are as follows:</u>

1. **# 9 Rationale for Each Change:**
    a. Amendment Request #2 – "Bent-over rows" is listed twice, while "deadlift", which is depicted on the next page, is not mentioned at all. Please correct.

        - I apologize for this. This has been changed.

b. The “full body workout” needs to be consistent throughout the informed consent, protocol, and data sheets.

- Where applicable, I have added ‘whole body workout to the consent form, protocol, and data collection sheet.

c. Amendment Request #3 – In this section, it is stated that the fat biopsy will be taken from the “upper buttock region,” but in the informed consent on page 11, it is said that it would be taken from the “left leg”. Also on page 6 of the informed consent, it was stated that the fat biopsy would be taken from the “hip.” Please make the location of fat biopsy consistent in all parts of the protocol and informed consent.

- I apologize for this inconsistency. It is the upper buttock region and this has been changed throughout.

d. Amendment Request #4 – Please include another bullet that states that an extra $150 will be given when the final biopsies are completed.

- This now reads:

“• $50 for completion of visits 1-2 (1 fat biopsy and 1 muscle biopsy given)
• An additional $100 for completion of visits 1-3 (1 fat biopsy and 2 muscle biopsies given)
• An additional $200 for completion of up to all visits except visit #39 (meaning that all workouts were completed but the last testing session was missed and, thus, the last muscle and fat biopsy was not taken)
• An additional $150 for completion of visit #39
If this is not what you’re aiming towards I can make further changes.”

**2. Informed Consent:**

a. What will be Involved:
   i. Change “3” longer testing sessions to “4” (T1, T2, T3, and T39).

- I apologize for this inconsistency. This has been changed.

b. Visit #1 and Visit #3:
   i. Change “1 tablespoon” to “1 teaspoon (5 mL).”

- I apologize for this inconsistency. This has been changed.

c. Risks or Discomforts:
   i. #1 should also include the fat biopsy as a procedure that could cause a hematoma.

- I apologize for this omission. This has been added.

ii. #6 should also include the ultrasound gel as a possible allergen for participants.

- I apologize for this omission. This has been added.

d. Compensation:

i. The bullets that are listed do not add up to $500. Please include another bullet that states that an extra $150 will be given when the final biopsies are completed.

- This has been changed.

ii. Include a note similar to the following: "Please be aware that compensation for participation in research may be subject to taxation. If you have any questions regarding taxation, please contact the Office of Procurement and Payment Services."

a. See page 38 on the following link for more information: https://sites.auburn.edu/admin/universitypolicies/policies/spendingpoliciesandprocedures.pdf

- I have included the following statement in the informed consent: "Please be aware that compensation for participation in research may be subject to taxation. If you have any questions regarding taxation, please contact the Office of Procurement and Payment Services (334-844-7771)."

iii. Include a question that asks whether the participant is an employee or a foreign national.

At the end of the IRB I have placed the following:

"For payment purposes please indicate here if you are a:

___________ employee

___________ foreign national

In both situations, taxes will be deducted prior to payment being received."

e. Costs for participating:

i. Change the current sentence to read: "In the unlikely event that you sustain an injury from participation in this study, the investigators have no current plans to provide funds for any medical expenses or other costs you may incur."

- I have made this change.

3. **Script and Advertisement:**
    a. Define what BCAAs are.
    b. Define location of fat biopsy throughout (as mentioned above).

        - I have made these changes.

4. **Data sheets:**
    a. Make the “full body workout” components consistent throughout.

        - I have made these changes.

5. **Medical Questionnaire:**
    a. Include a question about a possible allergy to ultrasound gel.
    b. Include a question about a possible allergy to lidocaine.
    c. Include a question about back/spine injury and/or surgery.

        - I have made these changes.

The IRB's comments are as follows:

*"1d and 2dI : Still need the 4th bullet to add up to $500*
*- $150 Visit 39 for final fat and muscle biopsies*

My apologies for not understanding this. I understand this now. I have made this change and it's on the tracked PDF copy here.

*2diii and in consent: Only ask about employees of Auburn University (not students) and about foreign nationals. The reason is payment and Procurement will take the tax deduction before they cut them a check in those situations."*

I have made this change and it's on the tracked PDF copy here.

# AUBURN UNIVERSITY

## CURRICULUM VITAE

# Michael D. Roberts, PhD

## CONTACT INFORMATION

Auburn University
School of Kinesiology
301 Wire Rd, Office 286
Auburn, AL 36849-5323
Phone: (334) 844-1844
E-mail: mdr0024@auburn.edu

## PROFESSIONAL EXPERIENCE

2017-pres. Associate Professor (tenured)
School of Kinesiology
Auburn University

2013-2017 Assistant Professor
School of Kinesiology
Auburn University

2013-pres. Director, Molecular and Applied Sciences Laboratory
School of Kinesiology
Auburn University

2014-pres. Adjunct Research Professor
Edward Via College of Osteopathic Medicine-Auburn University Campus

2010-2013 Postdoctoral Research Fellow
Booth Laboratory, Department of Biomedical Sciences
University of Missouri-Columbia

## EDUCATION

2010 PhD, Exercise Physiology
Department of Health and Exercise Sciences
University of Oklahoma

2006 MSEd, Exercise Physiology
Department of Health, Human Performance and Recreation
Baylor University

2003 BS, Biology
College of Arts and Sciences
Baylor University

# FIELD EXPERIENCE

2004-2006 Certified Personal Trainer
Gold's Gym International, Inc., Waco, TX
Credential: Certified Strength and Conditioning Specialist (CSCS), National Strength and Conditioning Association

# RESEARCH SUPPORT

## Summary

Total extramural funding directly procured as PI or critical co-I to date: $2,265,339

Additional monies procured as critical co-I: $58,645

- NIH T32, *listed below*
  (competitive internal funding at MU; PI: Dr. Ronald Terjung from University of Missouri)
- MU CVM Grant, *listed below*
  (co-I; PI: Dr. Frank Booth from University of Missouri)

## Monies obtained

**(listed from newest to oldest)**

2018-19 Extramural Contract: A prospective, randomized, double-blind, placebo-controlled, parallel group study on effects of a multiple vitamin/mineral/omega-3 fatty acid supplement on nutrient status/functionality, subjective perception of mood/energy/mental/skin health, and gene expression in adult women.
Funding Agency: Ritual
Total Costs (Direct+Indirect): $292,400
Site: Molecular and Applied Sciences Laboratory, Auburn University
Role: co-PI (co-PI: Dr. Kaelin Young from AU-VCOM, co-I: Dr. Rusty Arnold from Harrison School of Pharmacy at AU)

2018 Extramural Contract: Service contract for serum irisin and PICP analyses.
Funding Agency (Direct+Indirect): Applied Sports Science Institute
Total Costs: $5,986
Site: Molecular and Applied Sciences Laboratory, Auburn University
Role: PI

2018 Extramural Contract: Detecting muscle tissue and bone mineral content loss, growth, and maintenance at the cellular, segmental, and total-body level using bioimpedance spectroscopy using the SOZO and SFB7 devices.
Funding Agency (Direct+Indirect): Impedimed
Total Costs: $53,750
Site: Molecular and Applied Sciences Laboratory, Auburn University
Role: co-PI (co-PI: Dr. Kaelin Young from AU-VCOM)

2018 AU-VCOM Seed Grant: [no title].
Funding Agency: Edward Via College of Osteopathic Medicine - Auburn Campus
Total Costs (Direct only): $10,000
Site: Molecular and Applied Sciences Laboratory, Auburn University
Role: PI

2017-18 Extramural Gift/Donation: Laboratory Development Award
Funding Agency: Renaissance Periodization
Total Costs (Direct only): $20,000
Site: Molecular and Applied Sciences Laboratory, Auburn University
Role: co-PI (co-PI: Cody Haun, M.S.)

2017-18 Intramural Contract: Effects of dietary exosomes on muscle hypertrophy
Funding Agency: Auburn University's Intramural Grant Program
Total Costs (Direct only): $60,000
Role: co-PI (co-PI: Andreas Kavazis, co-I: Kaelin Young)

2017-20 American Diabetes Association Innovative Clinical of Translational Science Grant
Funding Agency: American Diabetes Association
Total amount procured for Auburn-Roberts (Direct+Indirect): $142,754
Role: co-I (PI: Dr. Martin from AU-VCOM, co-I: Dr. Beck for AU-VCOM)

2017 Extramural Contract: Effects of ketone salt ingestion on blood ketones and metabolic phenomena in humans
Funding Agency: Applied Sports Science Institute
Total Costs (Direct+Indirect): $39,343
Role: PI

2016 Extramural Gift/Donation: Laboratory Development Award
Funding Agency: FutureCeuticals
Total Costs (Direct only): $30,000
Site: Molecular and Applied Sciences Laboratory, Auburn University
Role: PI

2016 Extramural Contract: The effects of different ingredients on cGMP and nitric oxide production in HUVEC cells

Funding Agency: Purity Products
Total Costs (Direct + Indirect): $15,000
Site: Molecular and Applied Sciences Laboratory, Auburn University
Role: PI

2016 Extramural Contract: The effects of phosphatidic acid on myoblast properties
Funding Agency: ChemiNutra
Total Costs (Direct + Indirect): $7,400
Site: Molecular and Applied Sciences Laboratory, Auburn University
Role: PI

2016 Extramural Gift/Donation: Laboratory Development Award
Funding Agency: Bionutritional Research Group and Hilmar Ingredients
Total Costs (Direct only): $146,000
Site: Molecular and Applied Sciences Laboratory, Auburn University
Role: PI (co-I, Dr. Chris Lockwood)

2016 Extramural Contract: The effects of curcumin on nutritionally-induced non-alcoholic fatty liver disease and non-alcoholic steatohepatitis.
Funding Agency: DolCas Biotech, LLC
Total Costs (Direct + Indirect): $129,560
Site: Molecular and Applied Sciences Laboratory, Auburn University
Role: co-I (PI: Dr. Jeffrey Martin from AU-VCOM)

2016 Extramural Contract: The effects of Red Spinach Extract on variables related to blood flow and exercise performance (Part II)
Funding Agency: DolCas Biotech, LLC
Total Costs (Direct + Indirect): $10,339
Site: Molecular and Applied Sciences Laboratory, Auburn University
Role: co-I (PI: Jeffrey Martin from AU-VCOM)

2016 Extramural Contract: Effects of a novel plant extract on cycling performance, hemodynamics and markers of red blood cell physiology.
Funding Agency: FutureCeuticals
Total Costs (Direct + Indirect): $135,606
Site: Molecular and Applied Sciences Laboratory, Auburn University
Role: co-PI (co-PI: Dr. Kaelin Young from AU-VCOM, co-PI: Dr. Jeffrey Martin from AU-VCOM)

2016 Extramural contract: [no title], contract to assist in the dissemination of research.
Funding Agency: University of Mary Hardin-Baylor
Total Costs (Direct + Indirect): $1,600
Site: Molecular and Applied Sciences Laboratory, Auburn University
Role: PI

2015-16 Extramural Contract: Effect of various ingredients on skeletal muscle Irisin signaling and cross talk with adipose tissue.
Funding Agency: Maximum Human Performance (MHP)
Total Costs (Direct + Indirect): $73,575
Site: Molecular and Applied Sciences Laboratory, Auburn University
Role: PI

2015-16 Extramural contract: Effects of aging and nutrition on various physiological systems.
Funding Agency: University of Tampa and Human Longevity, Inc.
Total Costs (Direct + Indirect): $231,000
Site: Molecular and Applied Sciences Laboratory, Auburn University
Role: PI

2015 Extramural contract: [no title], contract to assist in the dissemination of research.
Funding Agency: University of Mary Hardin-Baylor
Total Costs (Direct + Indirect): $2,400
Site: Molecular and Applied Sciences Laboratory, Auburn University
Role: PI

2015 Extramural contract: [no title], contract to assist in the dissemination of research.
Funding Agency: University of Mary Hardin-Baylor
Total Costs (Direct + Indirect): $1,600
Site: Molecular and Applied Sciences Laboratory, Auburn University
Role: PI

2015 Extramural contract: The effects of Red Spinach Extract on variables related to blood flow and exercise performance.
Funding Agency: DolCas Biotech, LLC
Total Costs (Direct + Indirect): $28,022
Site: Molecular and Applied Sciences Laboratory, Auburn University
Role: co-I (PI: Jeffrey Martin from AU-VCOM)

2015-2016 AU-VCOM Seed Grant: The role of target inflation pressures on skeletal muscle gene expression and the efficacy of a peristaltic pulse external pneumatic compression device as an adjuvant to exercise training.
Funding Agency: Edward Via College of Osteopathic Medicine - Auburn Campus
Total Costs (Direct only): $30,318
Site: Molecular and Applied Sciences Laboratory, Auburn University
Role: co-I (PI: Jeffrey Martin from AU-VCOM)

2015 Extramural Contract: Effects of various ingredients on skeletal muscle Irisin signaling.
Funding Agency: Maximum Human Performance (MHP)
Total Costs (Direct + Indirect): $9,600
Site: Molecular and Applied Sciences Laboratory, Auburn University

Role: PI

2014-15 Extramural sub-contract: Effect of a subchronic ketogenic diet on skeletal muscle anabolic and catabolic signaling as well as adipose tissue signaling
Funding Agency: University of Tampa
Total Costs (Direct + Indirect): $105,000
Site: Molecular and Applied Sciences Laboratory, Auburn University
Role: PI

2014-15 Extramural Gift/Donation: Laboratory Development Award
Funding Agency: 4Life Research, Inc (collaborative efforts with Dr. Chris Lockwood)
Total Costs (Direct only): $100,000
Site: Molecular and Applied Sciences Laboratory, Auburn University
Role: PI

2015 Extramural Contract: Effects of Proprietary Weight Loss Supplement on Resting Metabolism
Funding Agency: MusclePharm, Corp.
Total costs (Direct+Indirect): $47,882
Site: Molecular and Applied Sciences Laboratory, Auburn University
Role: PI

2014-15 Extramural Contract: Effects of a branched-chain amino acid-carbohydrate-electrolyte solution on muscle damage during one week of rigorous training
Funding Agency: MusclePharm, Corp.
Total Costs (Direct + Indirect): $89,000
Site: Molecular and Applied Sciences Laboratory, Auburn University
Role: PI

2014 Extramural Contract: Myostatin, follistatin, and cytokine array assessment in human serum samples
Funding Agency: University of Tampa
Total Costs (Direct + Indirect): $8,800
Site: Molecular and Applied Sciences Laboratory, Auburn University
Role: PI

2014 Extramural Contract: Effects of pneumatic compression therapy on molecular markers of muscle metabolism
Funding Agency: Quinnipiac University
Total Costs (Direct + Indirect): $13,500
Site: Molecular and Applied Sciences Laboratory, Auburn University
Role: PI

2014 Extramural Contract Part 1: Studying the efficacy of ‘BCAA 3.1.2’ on cycling performance, body composition, and immune system markers in elite cyclists over

a training season; Part 2: Studying the effects of 'Combat' on post-exercise muscle-building mechanisms using a human-applicable rat model
Funding Agency: MusclePharm, Corp.
Total Costs (Direct + Indirect): $61,117
Site: Molecular and Applied Sciences Laboratory, Auburn University
Role: PI

2014 Extramural Contract: Effect of Myo-X and Arachidonic acid on Intramuscular Markers of Skeletal Muscle Anabolism
Funding Agency: sub-contract from University of Tampa
Total Costs (Direct + Indirect): $16,995
Site: Molecular and Applied Sciences Laboratory, Auburn University
Role: PI

2014 Extramural Contract: Effect of Different Protein Blends on Intramuscular Markers of Skeletal Muscle Anabolism
Funding Agency: Axiom Foods, Inc.
Total Costs (Direct + Indirect): $35,632
Site: Molecular and Applied Sciences Laboratory, Auburn University
Role: PI

2013-14 Extramural Gift/Donation: Laboratory Development Award
Funding Agency: 4Life Research, Inc (collaborative efforts with Dr. Chris Lockwood)
Total Costs (Direct only): $100,000
Site: Molecular and Applied Sciences Laboratory, Auburn University
Role: PI

2013 Extramural Contract: Effects of transfer factors and adaptogen blends on oxidative stress in rats fed a high fat diet
Funding Agency: 4Life Research, Inc
Total costs (Direct only): $49,230
Site: Booth Laboratory, University of Missouri
Role: PI

2012 Fellowship: T32 Postdoctoral Fellowship
Funding Agency: National Institutes of Health
Total costs: $40,645 (estimated)
Site: Booth Laboratory, University of Missouri
Role: awarded from internal institutional committee for 1-year of postdoctoral salary (PI: Dr. Ronald Terjung from University of Missouri)

2012 Extramural Contract: Effects of an oral ATP supplement on blood flow during exercise in rats
Funding Agency: TSI Health Sciences
Total costs (Direct only): $10,000

Site: Booth Laboratory, University of Missouri
Role: PI

2012 Extramural Contract: Effects of different whey protein forms on the post-prandial serum metabolome in rats
Funding Agency: Bionutritional Research Group
Total costs (Direct only): $42,000
Site: Booth Laboratory, University of Missouri
Role: PI

2012 Intramural Grant: University of Missouri College of Veterinary Medicine Grant
Title: Molecular determinants of running motivation in rats selectively bred to run high versus low nightly distances
Funding Agency: College of Veterinary Medicine, University of Missouri
Total costs (Direct only): $18,000
Site: Booth Laboratory, University of Missouri
Role: co-I and Project Coordinator (PI: Dr. Frank Booth from University of Missouri)

2011-2012 Extramural Contract: Effects of a proprietary whey protein hydrolysate on mammalian physiological systems
Funding Agency: Scivation Inc.
Total costs (Direct only): $100,000 over a 1.5-year period for personnel support and study supplies
Site: Booth Laboratory, University of Missouri
Role: PI

2007 Doctoral Research Award: The Effects of Aging on the Phosphocreatine System
Funding Agency: National Strength and Conditioning Foundation
Total costs (Direct only): $5,000
Site: Applied Biochemistry and Molecular Physiology Laboratory, University of Oklahoma
Role: PI

## Grants applied for but not funded

2016 National Institutes of Health R01 Grant: Dietary exosomes regulate muscle protein accretion in pigs
Funding Agency: National Institutes of Health
Amount requested for Roberts laboratory: $150,000 total over 2 years
Roberts's Role: co-Investigator (PI: Janos Zempleni, University of Nebraska)
Comments: not discussed

2016 National Institutes of Health U01 Grant: Molecular Transducers of

Exercise
Funding Agency: National Institutes of Health
Title: Acute and chronic molecular transducers to endurance/resistant physical training
Amount requested for Auburn-Roberts: $1,156,927
Role: co-I (PI: Dr. Frank Booth from University of Missouri)
Comments: scored 4 of 8 animal proposals, not funded

2015 American Diabetes Association Innovative Clinical of Translational Science Grant
Funding Agency: American Diabetes Association
Amount requested for Auburn-Roberts: $566,003
Role: co-I (PI: Dr. Martin from AU-VCOM)
Comments: not scored, resubmitted in 2016 with edits and additional pilot data

2014 AU Intramural IGP Grant: Establishing guidelines for step-count programs and understanding program adherence decisions
Amount requested: $54,404
Role: co-I (PI: Dr. Matthew Miller from Auburn University)
Comments: scored, but not funded

2014 Extramural Grant: Examining the synergistic effects of creatine and leucine on muscle anabolic and catabolic signaling in C2C12 myotubes concurrently treated with 'youth-like' and 'elderly-like' concentrations of testosterone
Funding Agency: International Society of Sports Nutrition
Amount requested: $10,000
Role: Principal Investigator
Comments: scored, but not funded

2011 MU Intramural Grant: University of Missouri Institute for Clinical and Translational Sciences Grant
Title: The effects of abruptly stopping daily physical activity on skeletal muscle circadian genes and downstream metabolic genes
Amount requested: $10,000
Role: Principal Investigator
Comments: scored, but not funded

2011 MU Intramural Grant: University of Missouri College of Veterinary Medicine
Grant
Title: Molecular determinants of running motivation in rats selectively bred to run high versus low nightly distances
Funder: College of Veterinary Medicine, University of Missouri
Amount requested: $18,000
Role: co-I (PI: Dr. Frank Booth from University of Missouri)
Comments: scored, but not funded; resubmitted with edits in 2012 and procured

2010 National Institutes of Health F32 NRSA Postdoctoral fellowship Grant
Title: Adipose tissue-endothelial cell communication
Amount requested: $144,209
Role: Principal Investigator
Comments: original submission scored in the 36th percentile; resubmission not scored

# PUBLICATION IMPACT

## 2004-present

Google Scholar

| | |
|---|---|
| H-index: | 28 (28 papers cited at least 28 times) |
| Total citations: | 2,426 |
| Past 5-yr citation: | 1,621 |

*Note that these Google Scholar metrics only include peer-reviewed journal articles and do not include published scientific abstracts (e.g., EB abstracts in FASEB J, ACSM abstracts in MSSE, or ISSN abstracts in JISSN) or book chapters*

ResearchGate

| | |
|---|---|
| H-index: | 22 (20 excluding self-citations) |
| Total citations: | 1,478 |
| Publication reads: | ~23,500 |

*Note that these ResearchGate metrics do include peer-reviewed journal articles as well as published scientific abstracts*

# SCHOLARLY CONTRIBUTIONS

## Original peer-reviewed research articles in PubMed-indexed journals

- **listed from newest to oldest**
- ***, indicates Roberts is corresponding or co-corresponding author**
- **†, indicates > 50 citations according to Google Scholar**
- **††, indicates > 100 citations according to Google Scholar**
- **#, indicates paper received an award (noted after the citation)**

1. Roberson P, Haun C, Mobley C, Romero M, Martin J*, **Roberts M***. Skeletal muscle amino acid transporter and BCAT2 expression prior to and following interval running or resistance exercise in mode-specific trained males. Accepted and in press at ***Amino Acids***, 2018.

2. Haun C, Mobley CB, Roberson P, Mumford P, Romero M, Kephart W, Anderson R, Vann C, Osburn S, Pledge C, Lockwood C, **Roberts M***. Serum sex steroid hormone and tissue receptor responses to resistance training and protein supplementation. Comments: Accepted and in press at ***Sci Reports (Nature Publishing Group)***, 2018.

3. Mumford P, Mao X, Mobley CB, Kephart W, Romero M, Haun C, Roberson P, Young K, Martin J, Beck D, **Roberts M***. Cross-talk between skeletal muscle androgen and Wnt signaling potentially contributes to age-related atrophy in rats. Comments: Accepted and in press at ***J Appl Physiol***, 2018.

4. Lowery R, Wilson J, Barniger A, Sharp M, Irvin C, Stefan M, Wallace W, Wilson G, **Roberts M**, Wagner R. The effects of soluble corn fiber and isomaltooligosacharides on blood glucose, insulin, digestion, and fermentation in healthy young males. ***J Insulin Res*** 3(1): https://doi.org/10.4102/jir.v3i1.32, 2018.

5. Mobley CB, Haun C, Roberson P, Mumford P, Kephart W, Romero M, Osburn S, Vann C, Young K, Beck D, Martin J, Lockwood C, **Roberts M***. Biomarkers associated with low, moderate, and high vastus lateralis muscle hypertrophy following 12 weeks of resistance training. ***PLOS One***, Apr 5;13(4):e0195203, 2018. PMID: 29621305

6. Colquhoun R, Magrini M, Haun C, Muddle T, Tomko P, Luera M, Mackey C, Vann C, Martin J, Young K, Defreitas J, **Roberts M***, Jenkins N*. Muscle phenotype is related to motor unit behavior of the vastus lateralis during maximal isometric contractions. ***Physiol Rep.*** 6(5): doi: 10.14814/phy2, 2018. PMID: 29527830

7. **#**, Romero M, Mobley CB, Roberson P, Haun C, Kephart W, Mumford P, Healy J, Young K, Beck D, Martin J, **Roberts M***. Acute and chronic resistance exercise down-regulate markers of LINE-1 retrotransposon activity in human skeletal muscle. ***AJP Cell Physiol***. 314(3): C379-C388, 2018. PMID: 29351416
   **#, received APS Select Award**

8. Rodriguez-Hernandez M, Martin J, Pascoe D, **Roberts M**, Wadsworth D. Multiple short bouts of walking activity attenuate blood glucose response in obese women. ***J Phys Act and Health***. 15(4): 279-286, 2018. PMID: 29421968

9. Mobley CB, Holland AM, Kephart W, Mumford P, Lowery R, Wilson J, **Roberts M***. Progressive resistance-loaded voluntary wheel running increases hypertrophy and differentially affects muscle protein synthesis, ribosome biogenesis, and proteolytic markers in rat muscle. ***J Anim Physiol Anim Nutr (Berl)***. 102(1):317-329, 2018. PMID 28294417

10. Kephart W, Roberson P, Pledge C, Mumford P, Romero M, Haun C, Mobley CB, Martin J, Young K, **Roberts M***. The three-month effects of a ketogenic diet on body composition, blood parameters, and performance metrics in recreationally-trained CrossFit trainees: a pilot study. ***Sports (MDPI)*** 6(1): doi:10.3390/sports6010001, 2018.

11. Haun C, **Roberts M**, Romero M, Osburn S, Healy J, Moore A, Mobley CB, Goodlett M, Pascoe D, Martin J. Concomitant external pneumatic compression treatment with consecutive days of high intensity interval training reduces markers of oxidative stress and proteolysis. ***Eur J Appl Physiol***. 117(12): 2587-2600, 2017. PMID: 29075862

12. Moore A, Haun C, Kephart W, Holland A, Mobley CB, Pascoe D, **Roberts M**, Martin J. A nitrate-rich red spinach extract increases the ventilatory threshold during graded exercise testing. ***Sports (MDPI)*** 5, 80, 2017. doi:10.3390 17.

13. Martin J, Mumford P, Haun C, Luera M, Muddle T, Colquhoun R, Freeney M, Mackey C, Roberson P, Young K, Pascoe D, Defrietas J, Jenkins N, **Roberts M**. Effects of a pre-workout supplement on hyperemia following leg extension resistance exercise to failure with different training loads. ***J Int Soc Sports Nutr*** 14(38), 2017. PMID: 28959158

14. Haun C, Mumford P, Roberson P, Romero M, Mobley CB, Kephart W, Anderson R, Colquhoun R, Muddle T, Luera M, Mackey C, Riffe J, Pascoe D, Young K, Martin J, Defrietas J, Jenkins N*, **Roberts M***. Molecular, neuromuscular, and recovery responses to light versus heavy resistance exercise in young men. ***Physiol Reports*** 5(18), 2017. PMID: 28963127

15. Kephart W, Mumford P, Mao X, Romero M, Hyatt H, Zhang Y, Young K, Martin J, McCullough D, D'Agostino D, Lowery R, Beck D, Quindry J, Wilson J, Kavazis A*, **Roberts M***. The 1-week and 8-month effects of a ketogenic diet or ketone salt supplementation on markers of multi-organ oxidative stress and mitochondrial function in rats. ***Nutrients (MDPI)***, 9(9), 1019; doi:10.3390/nu9091019, 2017. PMID: 28914762

16. Mobley CB, Haun C, Roberson P, Mumford P, Romero M, Kephart W, Anderson R, Vann C, Osburn S, Pledge C, Martin J, Young K, Goodlett M, Pascoe D, Lockwood C, **Roberts M***. Effects of whey, soy or leucine supplementation with 12 weeks of resistance training on strength, body composition, and skeletal muscle and adipose tissue histological attributes in college-aged males. ***Nutrients (MDPI)*** 9(9), 972, 2017. PMID: 28869573

17. Romero M, Mobley CB, Linden M, Meers G, Young K, Martin J, Rector RS, **Roberts M***. Endurance training lowers ribosome density despite increasing ribosome biogenesis markers in rodent skeletal muscle. ***BMC Res Notes*** 10(1):399, 2017. PMID: 28800772

18. Mobley CB, Mumford P, Kephart W, Haun C, Holland AM, Patel R, Anderson R, Langston G, Beck D, Martin J, Young K, Lowery R, Wilson J, **Roberts M***. Aging in rats differentially affects markers of transcriptional and translational capacity in plantaris and soleus muscle. ***Frontiers in Physiol***, 2017 Jul 20(8):518. PMID: 28775694

19. Haun C, **Roberts M**, Romero M, Osburn S, Anderson R, Langston G, Pascoe D, Martin J. Does external pneumatic compression treatment between bouts of overreaching resistance training sessions exert differential effects on molecular signaling and performance-related variables compared to passive recovery? An exploratory study. ***PLOS One***. Jun 29;12(6):e0180429. doi: 10.1371/journal.pone.0180429, 2017. PMID: 28662152

20. Kendall K, Hyde P, Fairman C, Hollaway K, Mumford P, Haun C, Mobley CB, Kephart W, Tribby A, Kimber D, Moon J, Beck D, **Roberts M**, Young K. A randomized, double-blind, placebo-controlled trial to determine the effectiveness and safety of a thermogenic supplement in addition to an energy-restricted diet in apparently healthy females. ***J Dietary Supplements*** 14(6), 2017. PMID: 28388294

21. Wilson J, Lowery R, **Roberts M**, Sharp M, Joy J, Shields K, De Souza E, Rauch J, Partl J, Volek J, D'Agostino D. The effects of ketogenic dieting on body composition, strength, power, and hormonal profiles in resistance training males. ***J Strength and Cond Res***, 2017. DOI: 10.1519/JSC.0000000000001935 [ePub ahead of print]. PMID: 28399015

22. Dalbo V, Teramoto M, **Roberts M**, Scanlan A. Positive self-perceptions of health in the presence of disease. ***Sports (MDPI)*** 5(2): 23, 2017.

23. Dalbo V, **Roberts M**, Mobley CB, Ballmann C, Kephart W, Fox C, Santucci V, Conover C, Beggs L, Balaez A, Hoerr F, Yarrow J, Borst S, and Beck D. Testosterone and trenbolone enanthate increase mature myostatin protein expression despite increasing skeletal muscle hypertrophy and satellite cell number in rodent muscle. ***Andrologia*** 49(3), 2017. PMID: 27246614

24. Urbina S, **Roberts M**, Kephart W, Villa K, Santos E, Olivencia A, Bennett H, Lara M, Foster C, Pupura M, Jaeger R, Taylor L, Wilborn C. Effects of twelve weeks of capsaicinoid supplementation on body composition, appetite and self-reported caloric intake in overweight individuals. ***Appetite*** 113:264-273, 2017. PMID: 28235621

25. Mobley CB, Mumford P, McCarthy J, Miller M, Young K, Martin J, Beck D, Lockwood C, **Roberts M***. Whey protein-derived exosomes increase protein synthesis and anabolism in C2C12 myotubes. ***J Dairy Sci*** 100(1):48-64, 2017. PMID: 28341051

26. Lockwood C, **Roberts M***, Dalbo V, Smith A, Kendall K, Moon J, Stout J. Effects of hydrolyzed whey versus other whey protein supplements on the physiological response to 8 weeks of resistance exercise in college-aged males. ***J Am Coll Nutr*** 36(1), 2017. PMID: 27710436

27. Hyatt H, Smuder A, Sollanek K, Morton A, **Roberts M**, Kavazis A. Comparative changes in antioxidant enzymes and oxidative stress in cardiac, fast twitch, and slow twitch skeletal muscles following endurance exercise training. ***Int J Physiol Pathophysiol Pharmacol*** 8(4), 2016. PMID: 28078055

28. Hyatt H, Kephart W, Holland AM, Mumford P, Mobley CB, Lowery R, **Roberts M**, Wilson J, Kavazis A. A Ketogenic Diet in Rodents Elicits Improved Mitochondrial Adaptations in Response to Resistance Exercise Training Compared to an Isocaloric Western Diet. ***Frontiers in Physiol*** 7:533, 2016. PMID: 27877138

29. Martin J, Kephart W, Haun C, McCloskey A, Shake J, Mobley CB, Goodlett M, Kavazis A, Zhang L, **Roberts M**. Impact of external pneumatic compression target inflation pressure on transcriptome-wide RNA expression in skeletal muscle. ***Physiol Reports*** 4 (22), 2016. PMID: 27884954

30. Hayward S, Wilborn C, Taylor L, Urbina S, Outlaw J, Foster C, **Roberts M**. Effects of a High Protein and Omega-3-Enriched Diet with or Without Creatine Supplementation on Markers of Soreness and Inflammation During 5 Consecutive Days of High Volume Resistance Exercise in Females. ***J Sports Sci and Med*** 15: 4, 704-714. 2016. PMID: 27928217

31. Wilborn C, Outlaw J, Mumford P, Urbina S, Hayward S, **Roberts M**, Taylor L, Foster C. A pilot study examining the effects of 8-week whey protein versus whey protein plus creatine supplementation on body composition and performance variables in resistance-trained women. ***Ann Nutr and Metab*** 69 (3-4): 190-199, 2016. PMID: 27866187

32. Haun C, Kephart W, Holland AM, Mobley CB, McCloskey A, Shake J, **Roberts M**, Martin J. Differential vascular reactivity responses acutely following ingestion of a nitrate rich red spinach extract. ***Eur J Appl Physio***l 116 (11-12): 2267-2279. 2016. PMID: 27695978

33. Townsend J, Stout J, Jajtner A, Church D, Beyer K, Oliveira L, La Moniac M, Riffe J, Muddle T, Baker K, Fukuda D, **Roberts M**, Hoffman J. Resistance exercise increases intramuscular NF-κB signaling in untrained males. ***Eur J Appl Physiol*** 116 (11-12):2103-2111, 2016. PMID: 27582262

34. Holland AM, **Roberts M**, Mumford P, Mobley CB, Kephart W, Conover C, Beggs L, Balaez A, Otzel D, Yarrow J, Borst S, Beck D. Testosterone inhibits expression of lipogenic genes in visceral fat by an estrogen-dependent mechanism. ***J Appl Physiol*** 121(3):792-805, 2016. PMID: 27539493

35. Kephart W, Mumford P, McCloskey A, Holland AM, Shake J, Mobley CB, Jagodinsky A, Weimar W, Oliver G, Young K, Moon J, **Roberts M***. Post-exercise branched chain amino acid supplementation does not affect recovery markers following three consecutive high intensity resistance training bouts compared to carbohydrate supplementation. ***J Int Soc Sports Nutr*** 13:30, 2016. PMID 27468258

36. Holland AM, Kephart W, Mumford P, Mobley CB, Lowery R, Shake J, Patel R, McCullough D, Kluess H, Huggins K, Kavazis A, Wilson J, **Roberts M***. Effects of a ketogenic diet on adipose tissue, liver, and serum biomarkers in sedentary rats and rats that exercised via resisted voluntary wheel running. ***Am J Physiol Regul Integr Comp Physiol*** 2016 Aug 1;311(2):R337-51. PMID: 27357802

37. Sharp M, Lowery R, Mobley CB, Fox C, De Souza E, Shields K, Healy J, Arick N, **Roberts M**, Wilson J. The effects of fortetropin supplementation on body composition,

strength and power in humans and mechanism of action in a rodent model. ***J Am Coll Nutr*** 35(8):679-691, 2016. PMID: 27333407

38. Neidert L, Mobley CB, Kephart W, **Roberts M**, Kluess H. The serine protease, dipeptidyl peptidase IV as a myokine: dietary protein and exercise mimetics as a stimulus for transcription and release. ***Physiol Rep*** 2016 Jun;4(12). PMID: 27335432

39. De Souza E, Lowery R, Wilson J, Sharp M, Mobley CB, Fox C, Lopez H, Shields K, Rauch J, Healy J, Thompson R, Ormes J, Joy J, and **Roberts M**. Effects of arachidonic acid supplementation on acute anabolic and chronic functional performance and body composition adaptations. ***PLOS One*** 2016 May 16;11(5):e0155153. PMID 27182886

40. **Roberts M***, Holland AM, Kephart W, Mumford P, Mobley CB, Lowery R, Fox C, McCloskey A, Shake J, Mesquita P, Patel R, Martin J, Young K, Kavazis A, Wilson J*. A putative low-carbohydrate ketogenic diet elicits mild nutritional ketosis but does not impair the acute or chronic hypertrophic responses to resistance exercise in rodents. ***J Appl Physiol*** 2016 May 15;120(10):1173-85. PMID 26718785

41. Taylor L, Wilborn C, **Roberts M**, McAdams M, White A, Woodall C, Dugan K, Roman S. Eight weeks of pre- and post-exercise whey protein supplementation increases lean body mass and improves performance in division III collegiate female basketball players. Appl Phys ***Nutr and Metab*** 2016 Mar;41(3):249-54. PMID 26842665

42. Mobley CB, Beck D, Kephart W, Conover C, Beggs L, Balaez A, Yarrow J, Borst S, and **Roberts M***. Effects of testosterone treatment on markers of skeletal muscle ribosome biogenesis *in vivo* and *in vitro*. ***Andrologia*** 2016 Jan 19. doi: 10.1111/and.12539. [Epub ahead of print]. PMID 26781353

43. Martin J, Kephart W, Mobley CB, Wilson T, Goodlett M, **Roberts M**. A single 60-min bout of peristaltic pulse external pneumatic compression transiently upregulates phosphorylated ribosomal protein s6. ***Clin Physiol Funct Imaging*** 2016 Jan 14. [Epub ahead of print]. PMID 26769680

44. Taylor L, Mumford P, **Roberts M**, Hayward S, Mullins J, Urbina S, Wilborn C. Safety of Teacrine, a non-habituating, naturally-occurring purine alkaloid over eight weeks of continuous use. ***J Int Soc Sports Nutr*** 2016 Jan 13;13:2. PMID 26766930

45. Ruegsegger G, Company J, Toedebusch R, Roberts C, **Roberts M**, and Booth F. Rapid alterations in perirenal adipose tissue transcriptomic networks with cessation of voluntary running. ***PLOS One*** 2015 Dec 17;10(12):e0145229. PMID 26678390

46. Hyatt H, Toedebusch R, Mobley CB, Fox C, McGinnis G, Quindry J, Booth F, **Roberts M**, Kavazis A. Comparative adaptations in oxidative and glycolytic muscle fibers in a low voluntary wheel running rat model performing three levels of physical activity. ***Physiol Rep*** 2015 Nov 3(11). pii: e12619. PMID 26603455

47. Kephart W, Wachs T, Thompson R, Mobley CB, Fox C, McDonald J, Ferguson B, Moon J, Young K, Nie B, Martin J, Company J, Pascoe D, Arnold R and **Roberts M***. Ten weeks of branched-chain amino acid supplementation improves select performance and immunological variables in trained cyclists. ***Amino Acids*** 2015 Nov 9. [Epub ahead of print]. PMID 26553453

48. Martin J, Friendenreich Z, Borges A, **Roberts M**. Preconditioning with peristaltic external pneumatic compression does not acutely improve repeated Wingate performance nor does it alter blood lactate concentrations during passive recovery compared with sham. ***Appl Phys Nutr and Metab*** 2015 Nov;40(11):1214-1217. PMID: 26489050

49. **Roberts M**, Mobley CB, Heese A, Toedebusch R, Company J, Zhu C, Hofheins J, Krieger A, Cruthirds C, Lockwood C, Leidy H, Kim D, Booth F and Rector RS. Western diet-induced hepatic steatosis and alterations in the liver transcriptome in adult Brown-Norway rats. ***BMC Gastroenterol*** 2015 Oct 30;15(1):151. PMID: 26519296

50. Mobley CB, Fox C, Thompson M, Healy J, Santucci V, Kephart W, McCloskey A, Kim M, Pascoe D, Martin J, Moon J, Young K, and **Roberts M***. Comparative effects of whey protein versus L-leucine on skeletal muscle protein synthesis and markers of ribosome biogenesis following resistance exercise. ***Amino Acids*** 2015 Oct 27. [Epub ahead of print]. PMID: 26507545

51. Martin J, Friendenreich Z, Borges A, **Roberts M**. Acute effects of peristaltic pneumatic compression on repeated anaerobic exercise performance and blood lactate clearance. ***J Strength Cond Res*** 2015 Oct;29(10):2900-6. PMID: 25756325

52. Holland AM, Hyatt H, Smuder A, Sollanek K, Morton A, **Roberts M**, and Kavazis A. Influence of endurance exercise training on antioxidant enzymes, tight junction proteins, and inflammatory markers in the rat ileum. ***BMC Res Notes*** 2015 Sept 30;8(1):514. PMID 26423686

53. Kerksick C, **Roberts M**, Dalbo V, Sunderland K. Intramuscular phosphagen status and the relationship to muscle performance across the age spectrum. ***Eur J Appl Physiol*** 2015 Aug 26 [Epub ahead of print] PMID 26307531

54. Mumford P, Tribby A, Poole C, Dalbo V, Scanlan A, Moon J, **Roberts M** and Young K. Effect of caffeine on golf performance and fatigue during a competitive tournament. ***Med Sci in Sport and Exerc*** 2015 Aug 17. [Epub ahead of print] PMID 26285020

55. Mobley CB, Hornberger T, Fox C, Healy J, Ferguson B, Lowery R, McNally R, Lockwood C, Stout J, Kavazis A, Wilson J and **Roberts M***. Effects of oral phosphatidic acid feeding with or without whey protein on muscle protein synthesis and anabolic signaling in rodent skeletal muscle. ***J Int Soc Sports Nutr*** 2015 Aug 16; 12:32. PMID 26279644

56. Kephart W, Mobley CB, Fox C, Pascoe D, Sefton J, Wilson T, Goodlett M, Sunderland K, Kavazis A, **Roberts M**, Martin J. An acute bout of whole-leg, peristaltic pulse external pneumatic compression upregulates PGC-1α mRNA and eNOS protein expression in human skeletal muscle tissue. ***Exp Physiol*** 2015 Jul 1;100(7):852-6. PMID 25982469

57. McGinnis G, Ballmann C, Peters B, Nannayakarra G, **Roberts M**, Amin R and Quindry J. Interleukin-6 mediates exercise preconditioning against myocardial ischemia reperfusion injury. Am J Physiol Heart. 2015 Jun 1;308(11):H1423-33. PMID 25820396

58. Mobley CB, Fox C, Ferguson B, Pascoe C, Healy J, McAdam J, Lockwood C and **Roberts M***. Effects of protein form and composition on postprandial markers of skeletal muscle anabolism, adipose tissue lipolysis, and hypothalamic gene expression. ***J Int Soc Sports Nutr*** 2015 Mar 13;12:14. PMID: 25792976

59. Mobley CB, Fox C, Ferguson B, Amin R, Dalbo V, Baier S, Rathmacher J, Wilson J and **Roberts M***. L-leucine, beta-hydroxy beta-methylbutyric acid (HMB) and creatine monohydrate prevent myostatin-induced decreases in myotube diameter. ***J Int Soc Sport Nutr*** 2014 Aug 13; 11:38. doi: 10.1186/1550-2783-11-38. PMID: 25132809

60. Joy J, Gundermann D, Lowery R, Jager R, McCleary S, Pupura M, **Roberts M**, Wilson S, Moore, J, Hornberger T and Wilson J. Phosphatidic acid enhances mTOR signaling and resistance exercise-induced hypertrophy. ***Nutr Metab (Lond)*** 2014 Jun 16;11:29. doi: 10.1186/1743-7075-11-29. PMID: 24959196

61. Jager R, **Roberts M**, Lowery R, Joy J, Cruthirds C, Lockwood C, Pupura M, Wilson J. Oral adenosine-5′-triphosphate (ATP) administration increases blood flow following exercise in animals and humans. ***J Int Soc Sports Nutr*** 2014 Jun 13;11:28. doi: 10.1186/1550-2783-11-28. PMID: 25006331

62. **Roberts M**, Toedebusch R, Wells K, Company J, Brown J, Cruthirds C, Heese A, Shu C, Rottinghaus G, Childs T and Booth F. Nucleus accumbens neuronal maturation differences in young rats bred for low versus high voluntary running behavior. ***J Physiol*** 2014 May 15;592(Pt 10):2119-35. doi: 10.1113/jphysiol.2013.268805. PMID: 24665095

63. Toedebusch R, **Roberts M**, Wells K, Company J, Kamosky K, Padilla J, Jenkins N, Booth F and Rector S. Unique transcriptomic signature of omental adipose tissue in Ossabaw swine: a model of childhood obesity. ***Physiol Genomics*** 2014 May 15;46(10):362-75. doi: 10.1152/physiolgenomics.00172.2013. PMID: 24642759

64. Mobley CB, Lockwood C, Toedebusch R, Company J, Heese A, Zhu C, Hofheins J, Krieger A, Cruthirds C, Wiedmeyer C, Kim D, Booth F and **Roberts M***. Herbal adaptogens combined with protein fractions from bovine colostrum and hen egg yolk reduce liver TNF-α expression and protein carbonylation in Western diet feeding in rats. ***Nutr Metab (Lond***. 2014 Apr 23;11:19. doi: 10.1186/1743-7075-11-19. PMID: 24822076

65. Company J, **Roberts M**, Toedebusch R, Cruthirds C and Booth F. Sudden decrease in physical activity evokes adipocyte hyperplasia in 70- to 77-day-old rats but not 49- to 56-day-old rats. ***Am J Physiol Regul Integr Comp Physiol*** 2013 Dec 15;305(12):R1465-78. doi: 10.1152/ajpregu.00139.2013. PMID: 24089381

66. **Roberts M***, Cruthirds C, Lockwood C, Pappan K, Childs T, Company J, Brown J, Toedebusch R and Booth F. Comparing serum responses to acute feedings of an extensively hydrolyzed whey protein concentrate versus its native whey source in rats: a metabolomics approach. ***Appl Physiol Nutr Metab*** 2014 Feb;39(2):158-67. doi: 10.1139/apnm-2013-0148. PMID: 24476471

67. Wilson J, Joy J, Lowery R, Lockwood C, **Roberts M**, Fuller J, De Souza E, Baier S, Wilson S and Rathmacher J. Effects of oral adenosine-5'-triphosphate supplementation on athletic performance, skeletal muscle hypertrophy and recovery in resistance-trained men. ***Nutr Metab (Lond)*** 2013 Sep 22;10(1):57. doi: 10.1186/1743-7075-10-57. PMID: 24330670

68. **Roberts M**, Brown J, Company J, Oberle L, Heese A, Toedebusch R, Wells K, Cruthirds C, Ferreira A, Childs T, Brown M and Booth F. Phenotypic and molecular differences between rats selectively-bred to voluntarily run high versus low nightly distances. ***Am J Physiol Regul Integr Comp Physiol*** 2013 Jun 1;304(11):R1024-35. PMID: 23552494

69. **†, Roberts M**, Bayless D, Childs T, Company J, Jenkins N, Padilla J, Martin J, Dalbo V, Booth F, Rector S and Laughlin MH. Elevated skeletal muscle irisin precursor FNDC5 mRNA in obese OLETF rats. ***Metabolism*** 2013 Aug;62(8):1052-6. doi: 10.1016/j.metabol.2013.02.002. PMID: 23498898

70. Kerksick C, **Roberts M**, Dalbo V, Kreider R and Willoughby D. Changes in skeletal muscle proteolytic gene expression after prophylactic supplementation of EGCG and NAC and eccentric damage. ***Food Chem Toxicol*** 2013 Nov;61:47-52. doi: 10.1016/j.fct.2013.01.026. PMID: 23376779

71. Sunderland K, **Roberts M**, Dalbo V and Kerksick C. Aging and sequential resistance exercise bout effects on housekeeping gene mRNA expression in human skeletal muscle. ***J Strength Cond Res*** 27(1): 1-7, 2013. PMID: 23085978

72. Padilla J, Jenkins N, **Roberts M**, Arce-Esquivel A, Martin J, Laughlin MH and Booth F. Differential changes in vascular mRNA levels between rat iliac and renal arteries produced by cessation of voluntary running. ***Exp Physiol*** 98(1): 337-47, 2013. PMID: 22709650

73. **Roberts M**, Dalbo V, Sunderland K and Kerksick C. Electrophoretic separation of myosin heavy chain isoforms using a modified mini gel system. ***J Strength Cond Res*** 26(12): 3461-8. 2012. PMID: 22709650

74. Dalbo V, **Roberts M**, Hassell S and Kerksick C. Effects of pre-exercise feeding on serum hormone concentrations and biomarkers of myostatin and ubiquitin proteasome pathway activity. ***Eur J Nutr*** 52.2: 477-487, 2013. PMID: 22476926

75. Toedebusch R, Childs T, Hamilton S, Crowley J, Booth F and **Roberts M***. Postprandial leucine and insulin responses and toxicological effects of a novel whey protein hydrolysate-based supplement in rats. ***J Int Soc Sports Nutr*** 9(1): 24, 2012. PMID: 22672725

76. **Roberts M**, Childs T, Brown J, Davis JW and Booth F. Early depression of Ankrd2 and Csrp3 mRNAs in the polyribosomal and whole-tissue fractions in skeletal muscle with decreased voluntary running. ***J Appl Physiol*** 112(8):1291-9, 2012. PMID: 22282489

77. **Roberts M**, Gilpin L, Parker K, Childs T, Will M and Booth F. Dopamine D1 receptor modulation in nucleus accumbens lowers voluntary wheel running in rats bred to run high distances. ***Physiol & Behavior*** 105(3):661-8, 2012. PMID: 22001493

78. Dalbo V, **Roberts M**, Sunderland K, Poole C, Stout J, Beck T, Bemben M and Kerksick C. Acute loading and aging effects on myostatin pathway activity after three sequential bouts of resistance exercise. ***J Gerontol A Biol Sci Med Sci*** 66(8):855-65, 2011. PMID: 21665986

79. Poole C, **Roberts M**, Dalbo V, Sunderland K and Kerksick C. Megalin and androgen receptor gene expression in young and old human skeletal muscle before and after three sequential exercise bouts. ***J Strength Cond Res*** 25(2):309-317, 2011. PMID: 21322835

80. **Roberts M**, Dalbo V, Sunderland K, Poole C, Hassell S and Kerksick C. Myogenic mRNA markers in young and old human skeletal muscle prior to and following sequential exercise bouts. ***App Physiol Nutr Metab*** 36:1-11, 2011. PMID: 21326383

81. Poole C, **Roberts M**, Tucker P, Dalbo V, Billbe B, Debolt N and Kerksick C. The combined effects of exercise and ingestion of a meal replacement in conjunction with a weight loss supplement on body composition and fitness parameters in college-aged men and women. ***J Strength Cond Res*** 25(1): 51-60, 2011. PMID: 21157390

82. Dalbo V, **Roberts M**, Hassell S, Brown R and Kerksick C. Effects of age on serum hormone concentrations and intramuscular proteolytic signaling before and after a single bout of resistance training. ***J Strength Cond Res*** 25(1):1-9, 2011. PMID: 21157391

83. **†,** Kerksick C, Wismann-Bush J, Fogt D, Thomas A, Taylor L, Campbell B, Wilborn C, Harvey T, **Roberts M**, La Bounty P, Galbreath M, Marcello B, Rasmussen C and Kreider R. Changes in weight loss, body composition and cardiovascular disease risk after altering macronutrient distributions during a regular exercise program in obese women. ***Nutr J*** 22(9):59, 2010. PMID: 21092228

84. **Roberts M**, Lockwood C, Dalbo V, Volek J and Kerksick C. Ingestion of a high molecular weight hydrothermally modified waxy maize starch alters metabolic responses to prolonged exercise in trained cyclists. ***Nutr*** 6:659-65, 2010. PMID: 20951003

85. **Roberts M**, Dalbo V, Sunderland K, Poole C, Hassell S and Kerksick C. IGF-1 splice variant and peptide expression patterns in young and old human skeletal muscle prior to and following sequential exercise bouts. ***Eur J App Physiol*** 110(5):961, 2010. PMID: 20668872

86. **Roberts M**, Dalbo V, Hassell S and Kerksick C. Effects of pre-exercise feeding on markers of satellite cell activation. ***Med Sci Sports Exerc*** 42(10):1861-9, 2010. PMID: 20216467

87. Dalbo V, **Roberts M**, Hassell S, Moon J and Kerksick C. Effects of a mineral antioxidant complex on clinical safety, body water, lactate response and aerobic performance in response to exhaustive exercise. ***Int J Sports Nutr Exer Metab*** 20(5): 381-392, 2010. PMID: 20975106

88. Dalbo V, **Roberts M**, Hassell S, Stout JR and Kerksick C. Effect of gender on the metabolic impact of a commercially available thermogenic drink. ***J Strength Cond Res*** 24(6): 1633-42, 2010. PMID: 20508469

89. **Roberts M**, Kerksick C, Dalbo V, Hassell S, Tucker P and Brown R. Molecular attributes of human skeletal muscle at rest and following unaccustomed exercise: an age comparison. ***J Strength Cond Res*** 24(5): 1161-8, 2010. PMID: 20440120

90. Kerksick C, Wilborn C, Campbell W, Harvey T, Marcello B, **Roberts M**, Parker A, Byars A, Greenwood L, Almada A, Kreider R and Greenwood M. The effects of creatine monohydrate supplementation with and without D-pinitol on resistance training adaptations. ***J Strength Cond Res*** 23(9): 2673-82, 2009. PMID: 19858753

91. Moon J, Smith A, Tobkin S, Lockwood C, Kendall K, Graef J, **Roberts M**, Dalbo V, Kerksick C, Cramer J, Beck T and Stout J. Total body water changes after an exercise intervention tracked using bioimpedance spectroscopy: a deuterium oxide comparison. ***Clin Nutr*** 28(5): 516-25, 2009. PMID: 19500888

92. Kerksick C, Thomas A, Campbell B, Taylor L, Wilborn C, Marcello B, **Roberts M**, Pfau E, Grimstevedt M, Opusunji J, Magrans-Courtney T, Rasmussen C, Wilson R and Kreider R. Effects of a popular exercise and weight loss program on weight loss, body composition, energy expenditure and health in obese women. ***Nutr Metab (Lond)*** 6:23, 2009. PMID: 19442301

93. **Roberts M**, Dalbo V, Hassell S and Kerksick C. The expression of androgen-regulated genes before and after a resistance exercise bout in younger and older men. ***J Strength Cond Res*** 23(4):1060-7, 2009. PMID: 19528872

94. Kerksick C, Wilborn C, Campbell B, **Roberts M**, Rasmussen C, Greenwood M and Kreider R. Early phase adaptations to a split-body, linear periodization resistance training program in college-aged and middle men. ***J Strength Cond Res*** 23(3): 962-71, 2009. PMID: 19387379

95. **Roberts M**, Dalbo V, Stout J and Kerksick C. Efficacy and safety of a popular energy drink after 28 days of ingestion. ***J Int Soc Sports Nutr*** 5:19, 2008. PMID: 18950510

96. Moon J, Tobkin S, Smith A, **Roberts M**, Ryan E, Dalbo V, Walter A, Cramer J and Stout J. Percent body fat estimations in college men using field and laboratory methods: A three-compartment model approach. ***Dyn Med*** 7:7, 2008. PMID: 18426582

97. **†,** Moon J, Tobkin S, **Roberts M**, Dalbo V, Kerksick C, Bemben M and Stout J. Total body water estimations in healthy men and women using bioimpedance spectroscopy: a deuterium oxide comparison. ***Nutr and Metab (Lond)*** 5:7, 2008. PMID: 18353180

98. Dalbo V, **Roberts M**, Stout J and Kerksick C. Acute effects of ingesting a commercial thermogenic drink on changes in energy expenditure and markers of lipolysis. ***J Int Soc Sports Nutr*** 5:6, 2008. PMID: 18289388

99. **Roberts M**, Wismann J, Taylor L, Wilborn C, Kreider R and Willoughby D. Effects of ingesting JavaFit Energy Extreme functional coffee on aerobic and anaerobic fitness markers in recreationally-active coffee consumers. ***J Int Soc Sports Nutr*** 4:25, 2007. PMID: 18067677

100. **Roberts M**, Iosia M, Kerksick C, Taylor L, Campbell B, Wilborn C, Harvey T, Jitomir J, Rasmussen C, Willoughby D and Kreider R. Effects of arachidonic acid supplementation on training adaptations and markers of muscle hypertrophy in resistance-trained males. ***J Int Soc Sports Nutr*** 4:21, 2007. PMID: 18045476

101. Moon J, Hull H, Tobkin S, Teramoto M, Karabulut M, **Roberts M**, Ryan E, Kim S, Dalbo V, Walter A, Smith A, Cramer J and Stout J. Percent body fat estimations in college women using field and laboratory methods: A three-compartment model approach. ***J Int Soc Sports Nutr*** 4:16, 2007. PMID: 17988393

102. **††,** Campbell B, **Roberts M**, Kerksick C, Wilborn C, Marcello B, Taylor L, Greenwood M and Kreider R. Pharmokinetics, safety, and effects on exercise performance of L-arginine alpha-ketoglutarate in trained adult men. ***Nutr*** 22 (9): 872-881, 2006. PMID: 16928472

## Peer-reviewed review articles and commentaries

- **listed from newest to oldest**
- ***, indicates Roberts is corresponding or co-corresponding author**
- **†, indicates > 50 citations according to Google Scholar**
- **††, indicates > 100 citations according to Google Scholar**
- **#, indicates paper received an award (noted after the citation)**

103. **Roberts M***, Ruegsegger G, Brown J, Booth F. Mechanisms Associated with Physical Activity Behavior: Insights from Rodent Experiments. ***Exer Sports Sci Rev***. 45(4): 217-222, 2017. PMID: 28704221

104. Dalbo V and **Roberts M**. Invited Commentary: The activity of satellite cells and myonuclei during eight weeks of strength training in young men with suppressed testosterone. ***Acta Physiologica (Oxford)*** 213(3):556-8, 2015. PMID: 25330255

105. **†,** Buford T, **Roberts M**, and Church T. Toward exercise as personalized medicine. ***Sports Med*** 43.3:157-165, 2013. PMID: 23382011

106. **Roberts M**, Company J, Brown J, Toedebusch R, Padilla J, Jenkins N, Laughlin MH and Booth F. Potential clinical translation of juvenile rodent inactivity models to study the onset of childhood obesity. ***Am J Physiol Regul Integr Comp Physiol*** 303(3): R247-58, 2012. PMID: 22696577

107. **††,** Booth F, Laye M and **Roberts M**. Lifetime sedentary living accelerates some aspects of secondary aging. ***J Appl Physiol*** 111(5):1497-504, 2011. PMID: 21836048

108. **Roberts M**, Dalbo V, and Kerksick C. Postexercise myogenic gene expression: are human findings lost during translation? ***Exer Sport Sci Rev*** 39(4):206-11, 2011. PMID: 21799423

109. Kerksick C and **Roberts M**. Supplements for endurance athletes. ***Strength Cond J*** 32(1): 55-64, 2010. doi: 10.1519/SSC.0b013e3181c16db9

110. Dalbo V, **Roberts M**, Lockwood C, Tucker P, Kreider R and Kerksick C. The effects of age on skeletal muscle and the phosphocreatine energy system: can creatine supplementation help older adults? ***Dyn Med*** 24(8): 6, 2009. PMID: 20034396

111. **†,** Dalbo V, **Roberts M**, Kerksick C and Stout J. Putting the myth of creatine supplementation leading to muscle cramps and dehydration to rest. ***British J Sport Med*** 42(7): 567-73, 2008. PMID: 18184753

112. **††,** Campbell B, Kreider RB, Ziegenfuss T, La Bounty P, **Roberts M**, Burke D, Landis J, Lopez H and Antonio J. Position stand: protein and exercise. ***J Int Soc Sports Nutr*** 4:8, 2007. PMID: 17908291

113. **†,** Campbell B, La Bounty P and **Roberts M**. The ergogenic potential of arginine. ***J Int Soc Sports Nutr*** 1:2, 2004. PMID: 18500948

## Articles in review process

**(*, indicates Roberts corresponding author or co-corresponding author)**

114. McAdam J, McGinnis K, Ory R, Young K, Fruge A, **Roberts M**, Sefton J. Estimation of Energy Balance from Quantification of Training Volume and Dietary Intake across 14 Weeks of Army Initial Entry Training. Comments: in review at ***J Int Soc of Sports Nutr***.

115. Cunningham R, Moore M, Moore A, Healy J, **Roberts M**, Rector RS, Martin J. Curcumin supplementation mitigates NASH development and progression in female Wistar rats. Comments: in review at ***Physiol Rep***.

116. Mumford P, Kephart W, Romero M, Mobley CB, Haun C, Healy J, Moore A, Pascoe D, Ruffin W, Beck D, Martin J, **Roberts M***, Young K*. Sub-chronic effects of betalain supplementation on cycling performance and select physiological parameters. Comments: in review at ***Eur J Appl Physiol***.

117. McGinnis K, McAdam J, Lockwood C, Young K, **Roberts M**, Sefton J. Impact of Protein or Carbohydrate Supplementation on Musculoskeletal Injury Rates, Severity and Training Days missed in Initial Entry Training. In review at ***J Athletic Train***.

## Articles in preparation

**(*, indicates Roberts corresponding author or co-corresponding author)**

118. McAdam J, McGinnis K, Beck D, Haun C, Romero M, Mumford P, Roberson P, Young K, Lockwood C, **Roberts M**, Sefton J. Effects of high-dose whey protein supplementation on performance and body composition in soldiers during 9 weeks of Army initial entry training. Comments: Written and will be submitted to ***Med Sci in Sport and Exerc*** in Apr 2018.

119. **Roberts M***, Romero M, Mobley CB, Mumford P, Roberson P, Haun C, Vann C, Osburn S, Holmes H, Greer R, Lockwood C, Parry H, Kavazis A. Differences in biomarkers related to skeletal muscle myofibrillogenesis and mitochondrial physiology in high versus low responders to 12 weeks of resistance exercise training. Written and will be submitted to ***J Appl Physiol*** in Apr 2018.

120. Parry H, Kephart W, Mumford P, Romero M, Mobley C, Zhang Y, **Roberts M**, Kavazis A. Lifelong ketogenic diet increases longevity and liver and skeletal muscle mitochondria volume without altering oxidative stress markers in rats. Written and will be submitted in Apr 2018.

121. **Roberts M***, Mobley C, Haun C, Mumford P, Romero M, Roberson P, Vann C, McCarthy J. Physiological differences between low versus high anabolic responders to resistance exercise training: current perspectives and future research directions. Written and will be submitted to ***Frontiers in Physiol*** in June 2018.

122. Haun C, Cimini B, Thomas D, **Roberts M**, Carpenter A. CellProfiler$^{TM}$ software for skeletal muscle histological analysis. Comments: data collection complete and will be submitted for review in 2018.

123. Kerksick C, **Roberts M**, Campbell B, Galbreath M, Taylor L, Wilborn C, Dove J, Bunn J, Rasmussen C, Kreider R. Calcium with or without vitamin D supplementation does not enhance diet-induced body composition changes in overweight post-menopausal women during 14 weeks of exercise training. Comments: preliminary draft written and will be submitted for review in 2018.

124. Kephart W, Vann C, Mao X, Mumford P, Romero M, Haun C, Mobley CB, Roberson P, D'Agostino D, Lowery R, Wilson J, **Roberts M***. The 1-week and 8-month effects of a ketogenic diet or ketone salt supplementation on skeletal muscle and adipose tissue physiology in rats. Comments: data collection complete and will be submitted for review in 2018.

## Book Chapters

1. Quindry J**, Roberts M**. Chapter 14: Endurance Phenotype Primer. The Routledge Handbook of Sport and Exercise Systems Genetics. Lightfoot T, Roth S, Hubal M (Eds). *Publisher to be determined* (2018)

2. **Roberts M**, Haun C. Chapter 2: Bioenergetics. CSCCa Training Principles. Nesser T (Ed). *Publisher to be determined* (2018)

3. **Roberts M,** Dalbo V, and Buford T. Training and Nutrition Needs of the Older Strength / Power Athlete. *Nutrition and Performance in Masters Athletes*. Reaburn P (Ed). CRC Press: New York (2014)

4. Dalbo V and **Roberts M**. Ergogenic Aids for Masters Athletes. *Nutrition and Performance in Masters Athletes*. Reaburn P (Ed). CRC Press: New York (2014)

5. **Roberts M**, Company J, Campbell B. Fatty acid supplements. *Sports Nutrition: Enhancing Sports Performance*. Campbell B (Ed). CRC Press: New York (2013)

6. Taylor L and **Roberts M**. Introduction: The Role of the Training Table. *Nutritional Guidelines for Athletic Performance: The Training Table*. Taylor L (Ed). CRC Press: New York (2012)

7. **Roberts M**. Calorie Needs for Inducing Muscle Hypertrophy in *Nutritional Guidelines for Athletic Performance: The Training Table*. Taylor L (Ed). CRC Press: New York (2012)

8. Dalbo V and **Roberts M**. Calorie needs for improving body composition in *Nutritional Guidelines for Athletic Performance: The Training Table*. Taylor L (Ed). CRC Press: New York (2012)

9. **Roberts M.** Steroids/Precursor Hormones/Banned Substances: Playing Russian Roulette. *The Misled Athlete*. Germano C (Ed). iUniverse: Indiana (2011)

10. **Roberts M** and Kerksick C. Vitamins/Minerals: Invaluable Cellular Components for Optimal Physiological Function. *Nutrient Timing: Metabolic Optimization for Health, Performance and Recovery*. Kerksick C (Ed). CRC Press: New York (2011)

## Non-refereed ('mainstream') publications

1. **Roberts M.** Are my genes to blame? www.fitnesspudding.com. 2013

2. **Roberts M.** My favorite pre-workout stack. www.scivation.com. 2011

3. Lockwood C, **Roberts M,** Feliciano J, and Stoppani J. Supplements: the next generation. Muscle and Fitness: 70(5), May 2009.

4. **Roberts M** and Dalbo V. Creatine: white meat or water weight? Body of Science 2(2), 2008.

5. **Roberts M**. Arachidonic acid: the new mass builder. www.bodybuilding.com. 2008

6. **Roberts M**. and Llewellyn B. Arachidonic acid: the new mass builder. Muscular Development. December 2007

# COURSES TAUGHT

## Undergraduate Courses

2013-2015 KINE 4630, NSCA CSCS Prep Course
Auburn University, School of Kinesiology

2013-2014 KINE 4600, Strength Development
Auburn University, School of Kinesiology

2006-2010 HES 4873, Principles of Strength and Conditioning
University of Oklahoma, Dept of Health and Exercise Science

2009 HES 1823, Scientific Principles of Health and Disease
University of Oklahoma, Dept of Health and Exercise Science

2009 HES 3873, Principles of Personal Training
University of Oklahoma, Dept of Health and Exercise Science

## Graduate Courses

2016-pres. KINE 7710, Advanced Laboratory Techniques
Auburn University, School of Kinesiology

| | |
|---|---|
| 2015-pres. | KINE 7970, Special Topics: Nutrient Timing for Performance Optimization<br>Auburn University, School of Kinesiology |
| 2015-pres. | KINE 8970, Special Topics: Exercise Genetics Primer<br>Auburn University, School of Kinesiology |
| 2014 | KINE 8970, Special Topics: Molecular Exercise Science<br>Auburn University, School of Kinesiology |
| 2014-pres. | KINE 7970, Legal and Illegal Sports Supplements<br>Auburn University, School of Kinesiology |
| 2011, 2013 | MPP 9435 (co-taught, directed by Frank Booth, PhD), Skeletal Muscle<br>University of Missouri, Dept of Biomedical Sciences |
| 2008 | HES 5000 (co-taught), Exercise and Nutritional Biochemistry<br>University of Oklahoma, Dept of Health and Exercise Science |

# MENTORING EXPERIENCE

## Past PhD students

C. Brooks Mobley — PhD level — 2013-2017
- Current position: Postdoctoral Fellow; University of Kentucky (mentor: Dr. John J. McCarthy)

Wesley Kephart — PhD level — 2014-2017
- Current position: Assistant Professor; University of Wisconsin-Whitewater (tenure-track)

Maleah Holland — PhD level — 2014-2016
- Current position: Assistant Professor; Augusta University (tenure-track)

## Past Masters students

Xuansong Mao — Masters level — 2016-2017
- Current position: PhD student; University of Missouri-Columbia (mentor: Dr. Frank Booth)

## Primary mentoring of graduate students (current)

| | | |
|---|---|---|
| Cody Haun | PhD level | 2015-pres. |
| Petey Mumford | PhD level | 2015-pres. |
| Matt Romero | PhD level | 2016-pres. |
| Paul Roberson | PhD level | 2016-pres. |
| Carlton Fox | PhD level | 2018-pres. |
| Christopher Vann | Masters/PhD level | 2017-pres. |
| Shelby Osburn | Masters level | 2017-pres. |

## Primary mentoring of undergraduate students (current and completed)

| | | |
|---|---|---|
| Carlton Fox | Undergraduate level | 2013-2015 |
| Taylor Wachs | Undergraduate level | 2014 |
| Richard Thompson | Undergraduate level | 2014 |
| James Healy | Undergraduate level | 2013-2014 |
| Vincent Santucci | Undergraduate level | 2014 |
| Anna McCloskey | Undergraduate level | 2014-2015 |
| John Parker | Undergraduate level | 2014-2015 |
| Paulo Mesquita | Undergraduate level | 2014-2015 |
| Joshua Shake | Undergraduate level | 2014-2015 |
| Romil Patel | Undergraduate level | 2015-2017 |
| Shelby Coburn | Undergraduate level | 2015-pres. |
| Richard Anderson | Undergraduate level | 2015-2017 |
| David Baumohl | Undergraduate level | 2015-2017 |
| Drew Solorzano | Undergraduate level | 2016 |
| Christopher Vann | Undergraduate level | 2016-2017 |
| Casey Sexton | Undergraduate level | 2016-pres. |

## Oversight of salaried laboratory technicians

| | | |
|---|---|---|
| C. Brooks Mobley | Laboratory Technician | 2018 |
| Anna McCloskey | Laboratory Technician | 2015-2016 |
| James Healy | Laboratory Technician | 2016-2017 |

## Dissertation Committee Member
**(Committee Chair in parentheses)**

*Completed*

| | | |
|---|---|---|
| C. Brooks Mobley (**Roberts**) | PhD level | 2017 |
| Leslie Neidert (Kluess) | PhD level | 2017 |
| Hayden Hyatt (Kavazis) | PhD level | 2017 |
| Mynor Rodriguez (Wadsworth) | PhD level | 2017 |
| Wesley Kephart (**Roberts**) | PhD level | 2017 |
| Jeremy Townsend (Stout)* | PhD level | 2016 |
| A. Maleah Holland (**Roberts**) | PhD level | 2016 |
| Vandre Figueiredo (Cameron-Smith)† | PhD level | 2016 |
| Ruru Li (Huggins) | PhD level | 2016 |
| Graham McGinnis (Quindry) | PhD level | 2014 |
| Chris Ballmann (Quindry) | PhD level | 2014 |
| Bridget Peters (Quindry) | PhD level | 2014 |

*, indicates external Committee Member for University of Central Florida
†, indicates external Reader for the University of Auckland

*Ongoing*

| | | |
|---|---|---|
| Jeremy McAdam (Sefton) | PhD level | 2016-pres. |
| Cody Haun (**Roberts**) | PhD level | 2017-pres. |
| Matthew Romero (**Roberts**) | PhD level | 2017-pres. |
| Petey Mumford (**Roberts**) | PhD level | 2017-pres. |

Ashley Peart (Wadsworth) PhD level 2017-pres.

# PRESENTATIONS

## Invited Conference and Symposium Lectures

1. Title: 'Over-the-counter supplements that affect muscle mass.'
   Conference: AAPM&R (Orlando, FL); 2018

2. Title: 'Jumping genes: a new paradigm of muscle aging'
   Venue: Hilliard Discussion 8 (HD8), Huffines Institute for Sports Medicine and Human Performance at Texas A&M University (College Station, TX); 2018

3. Title: 'Whey protein-derived exosomes increase protein synthesis and hypertrophy in C2-C12 myotubes'
   Conference: Integrative Physiology of Exercise Meeting (San Diego, CA); 2018

4. Title: 'Are animal models applicable to sports nutrition research?'
   Conference: 15th International Society of Sports Nutrition Conference (Clearwater, FL); 2018

5. Title: 'A critical evaluation of assessing skeletal muscle hypertrophy.'
   Conference: American College of Sports Medicine Annual Meeting (Minneapolis, MN); 2018

6. Title: 'Over-the-counter supplements that affect muscle mass.'
   Conference: AAPM&R (Denver, CO); 2017

7. Title: 'Protein Supplementation for the Tactical Athlete.'
   Conference: Warrior Research Center Research Summit (Auburn, AL); 2017

8. Title: 'Ribosome Biogenesis 101.'
   Conference: American College of Sports Medicine Annual Meeting (Denver, CO); 2017

9. Title: 'Effects of exercise modality and post-exercise nutrition on markers of ribosome biogenesis in skeletal muscle.'
   Conference: Experimental Biology (Chicago, IL); 2017

10. Title: 'Ketogenic dieting with the intent of improving metabolic outcomes.'
    Venue: Auburn University's College of Veterinary Medicine Seminar Series (Auburn, AL); 2017

11. Title: 'Ketogenic dieting with the intent of improving metabolic outcomes.'
    Venue: Baylor University's Biomedical Sciences Seminar (Waco, TX); 2017

12. Title: 'Ketogenic dieting as an adjuvant to exercise-induced weight loss.'

Conference: UAB's Center for Exercise Medicine 2nd Annual Symposium (Birmingham, AL); 2016

13. Title: 'Counterpoint: Nutrition and muscle gains, does leucine content matter?'
Conference: 12th International Society of Sports Nutrition Conference (Austin, TX); 2015

14. Title: 'Dietary protein as a hormone.'
Conference: Southeastern Chapter of the American College of Sports Medicine Meeting (Jacksonville, FL); 2015

15. Title: 'To post doc or not to post doc.'
Conference: American College of Sports Medicine Meeting (San Diego); 2015

16. Title: 'High versus low voluntary running rat model and its implications for human translational research'
Conference: UAB's Center for Exercise Medicine 2nd Annual Symposium (Birmingham, AL); 2014

17. Title: 'Molecular updates on phosphatidic acid: muscle physiology and beyond.'
Conference: 11th International Society of Sports Nutrition Conference (Clearwater Beach, FL); 2014

18. Title: 'Protein supplementation for elite performance.'
Venue: Online Broadcast to Stanford University's Division of Sports Performance; 2014

19. Title: 'Protein supplementation for elite performance.'
Venue: U.S. Army Rangers briefing (6 briefings), Fort Benning, GA; 2013-2014, 2017

20. Title: 'Comparison of WPH vs. Other Whey Protein Forms: What the Science Tells Us.'
Conference: 10th International Society of Sports Nutrition Conference (Colorado Springs, CO); 2013

21. Title: 'Using selective breeding to make couch potatoes versus super-athletes: what we've learned so far.'
Venue: Nutritional Sciences Seminar, University of Missouri-Columbia Medical School; 2012

22. Title: 'Laboratory evidence examining the positive effects of physical activity in disease prevention.'
Conference: 35th National Strength and Conditioning Association Conference (Providence, RI); 2012

23. Title: 'The role of amino acids in complementing activity-induced exercise adaptations.'
Venue: Nutritional Sciences Seminar, University of Missouri-Columbia Medical School; 2011

24. Title: ‘Molecular adaptations to muscle hypertrophy.’
Conference: 33rd National Strength and Conditioning Association Conference (Orlando, FL); 2010

25. Title: ‘Post-exercise inflammation: friend of foe?’
Conference: Strength Pro Summit at *The Arnold Classic* (Columbus, OH); 2008

### Abstract Presentations at National/International Conferences

*Over 150, available upon request*

## SERVICE AND AWARDS

### Peer-reviewed Journal Editorial Boards

| | |
|---|---|
| 2017-pres. | Review Editor<br>Journal: Frontiers in Physiology, Sport and Exercise Nutrition section |
| 2017 | Invited Section Editor (with Dr. Vincent Dalbo)<br>Journal: Sport (MDPI) |
| 2015-pres. | Editorial Board Member<br>Journal: Frontiers in Physiology, Exercise Physiology section |
| 2011-pres. | Editorial Board Member<br>Journal: Journal for the International Society of Sports Nutrition |

### University Service

| | |
|---|---|
| 2018 | Hiring Committee Member for Exercise Physiology Faculty Member<br>School of Kinesiology, Auburn University |
| 2016 | Hiring Committee member for Health Disparities Cluster Hiring Initiative Faculty Member<br>School of Kinesiology, Auburn University |
| 2016 | Hiring Committee Member for Biomechanics Faculty Member<br>School of Kinesiology, Auburn University |
| 2016 | External Committee Member for Cluster Hiring Initiative, Metabolomics Faculty Member<br>Department of Animal Sciences, Auburn University |
| 2016 | Auburn University Intramural Grants Program Reviewer |
| 2015 | Auburn University Intramural Grants Program Reviewer |

| | |
|---|---|
| 2014 | Via Osteopathic School of Medicine-Auburn hiring committee for Department of Cell Biology and Physiology Faculty (involved in 4 faculty hires) |
| 2014 | Ad hoc committee member on AU-KINE graduate admissions |
| 2013-pres. | Committee member on AU-KINE Physical Activity and Health Curriculum Program Committee |
| 2012 | President<br>University of Missouri Postdoctoral Association<br>University of Missouri-Columbia |
| 2011-2013 | Events co-chair<br>University of Missouri Postdoctoral Association<br>University of Missouri-Columbia |

## Auburn School of Kinesiology Seminars Organized

| | |
|---|---|
| Fall 2017 | Nick Shaw (CEO, Renaissance Periodization)<br>Lecture regarding Online Fitness Industry |
| Fall 2017 | Dr. John McCarthy (University of Kentucky)<br>Lecture on miRNAs affecting muscle tissue physiology |
| Fall 2016 | Dr. Gabriel Wilson (Maximum Human Performance)<br>Lecture on Sports Nutrition Industry |
| Fall 2015 | Dr. Marcas Bamman (University Alabama-Birmingham)<br>Lecture on exercise and regenerative medicine |
| Fall 2015 | Dr. Frank Booth (University of Missouri)<br>Lecture on AICAR effects on aerobic fitness in rodents |
| Spring 2015 | Dr. John McCarthy (University of Kentucky)<br>Lecture on miRNAs affecting muscle tissue physiology |
| Fall 2014 | Dr. Frank Booth (University of Missouri)<br>Lecture on Physical Activity and Health and NIH funding |
| Fall 2014 | Dr. Jordan Moon (Muscle Pharm Research Institute)<br>Lecture on Body Composition Research |
| Fall 2014 | Dr. Richard Kreider (Texas A&M University)<br>Lecture on Nutritional Supplement Research |

| | |
|---|---|
| Fall 2013 | Dr. Mark Faries (Stephen F. Austin University)<br>Lecture on Adherence to Physical Activity |
| Fall 2013 | Dr. Chris Lockwood (4Life Research, Inc.)<br>Lecture on Career Development |

## Other Service

| | |
|---|---|
| 2017 | Chair of Basic Science Thematic Poster Presentations<br>National meeting for the American College of Sports Medicine |
| 2016 | Chair of Metabolism Thematic Poster Presentations<br>Southeastern chapter of the American College of Sports Medicine |
| 2015 | *Ad hoc* Masters Student abstract reviewer<br>Southeastern chapter of the American College of Sports Medicine |
| 2015 | Chair of Genetics Thematic Poster Session<br>National meeting for the American College of Sports Medicine |
| 2010-pres. | *Ad hoc* Grant and National Conference Abstract Reviewer<br>National Strength and Conditioning Association |
| 2011-2012 | Scientific Advisory Board Member (non-paid)<br>Scivation, Inc. |
| 2009-2010 | Writer and Contributor<br>Muscle and Fitness magazine |

## Professional Organization Memberships

| | |
|---|---|
| 2008-10, 2017-pres. | American Physiological Society |
| 2009, 2014-pres. | American College of Sports Medicine |
| 2014-pres. | American College of Sports Medicine: Southeastern Chapter |
| 2004-08, 2014-2016. | International Society of Sports Nutrition |
| 2007, 2009 | American College of Sports Medicine: Central States Chapter |
| 2004, 2006-2008 | National Strength and Conditioning Association |
| 2005-2006 | American College of Sports Medicine: Texas Chapter |

## Honors and Awards

| | |
|---|---|
| 2018 | Emily and Gerald Leischuck Graduate Teaching Award<br>College of Education<br>Auburn University |
| 2017 | Outstanding Faculty Early Career Award<br>College of Education<br>Auburn University |
| 2012 | M. Harold Laughlin Scholarship Award<br>(*denotes Outstanding Postdoctoral Fellow in Biomedical Sciences/Kinesiology/Medical School*)<br>School of Medicine and College of Veterinary Medicine<br>University of Missouri |
| 2011-2013 | Recipient of NIH Loan Repayment Award<br>National Institutes of Health |
| 2007 | Recipient of Doctoral Research Grant<br>National Strength and Conditioning Foundation |

## Student Honors and Awards

| | |
|---|---|
| 2018 | C. Brooks Mobley<br>Selected as 1 of Top 10 Graduate Students in the Graduate School<br>Auburn University |
| 2018 | Paul Roberson<br>Placed 1st overall at SEACSM PhD student poster awards<br>School of Kinesiology, Auburn University<br>($300 award) |
| 2017 | C. Brooks Mobley<br>Graduate Student of the Year<br>School of Kinesiology, Auburn University |
| 2017 | C. Brooks Mobley<br>Kochan Fund for Excellence Graduate Award<br>Auburn University<br>($1,250 award) |
| 2017 | C. Brooks Mobley<br>Doctoral Scholar Award<br>American Kinesiology Association |
| 2017 | Wesley Kephart<br>Presidential Award |

(Graduate Student of the Year for Auburn University)
Auburn University
($1,000 award)

2017 Wesley Kephart
Selected as 1 of Top 10 Graduate Students in the Graduate School
Auburn University

2017 Paul Roberson and Wesley Kephart (co-first authors)
Won best poster presentation at Boshell Diabetes Conference
Auburn University
($500 award)

2016 Wesley Kephart
Graduate Student of the Year
School of Kinesiology, Auburn University

2016 Cody Haun
1 of 3 best posters at UAB Exercise is Medicine Conference
($500 award)

2015 Wesley Kephart
Won best poster presentation at the International Society of Sports Nutrition meeting ($1,000 award)

2015 C. Brooks Mobley
1 of 3 best posters at UAB Exercise is Medicine Conference
($500 award)

2014 C. Brooks Mobley
Placed 2$^{nd}$ overall at SEACSM graduate student poster awards
($500 award)

2013 C. Brooks Mobley
1 of 8 finalists for best Masters Student awarded by the American Kinesiology Association

# PROFESSIONAL REFERENCES

## Current or past departmental co-workers

**L. Bruce Gladden, PhD**
Professor, School of Kinesiology
Auburn University
Phone: 334-844-1466
e-mail: gladdlb@auburn.edu

**Andreas N. Kavazis, PhD**
Associate Professor, School of Kinesiology
Auburn University
Phone: 334-844-1479
e-mail: ank0012@auburn.edu

**John C. Quindry, PhD**
Department Chair and Associate Professor, Department of Health and Human Performance
University of Montana
Phone: 406-243-4268
e-mail: john.quindry@mso.umt.edu

## Colleagues in field

**Marcas Bamman, PhD**
Professor, Departments of Physiology and Biophysics
UAB School of Medicine
Phone: 205-996-7937
e-mail: mbamman@uab.edu

**Arny Ferrando, PhD**
Professor, Department of Geriatrics
University of Arkansas Medical School
Phone: 205-996-7937
e-mail: aferrando@uams.edu

## Former post-doc mentor

**Frank W. Booth, PhD**
Professor, Biomedical Sciences
University of Missouri-Columbia
Phone: 573-882-6652
e-mail: boothf@missouri.edu
