# Supplementary material for: Soy protein supplementation is not androgenic or estrogenic in college-aged men when combined with resistance exercise training
Source: Sci Rep. 2018 Jul 24;8:11151. doi: 10.1038/s41598-018-29591-4 (PMC6057888; doi:10.1038/s41598-018-29591-4)
Supplement: Supplementary file 2 — Supplementary figures and tables [file 41598_2018_29591_MOESM2_ESM.docx]

Soy protein supplementation is not adipogenic or estrogenic in college-aged men when combined with resistance training

Cody T. Haun^1^_,_ C. Brooks Mobley^1^, Christopher G. Vann^1^_,_ Matthew A. Romero^1^, Paul A. Roberson^1^, Petey W. Mumford^1^, Wesley C. Kephart^1^, James C. Healy^1,2^, Romil K. Patel^1^_,_ Shelby C. Osburn^1^, Darren T. Beck^1,2^, Robert D. Arnold^4^, Ben Nie^4^, Christopher M. Lockwood^3^, Michael D. Roberts^1, 2*^

^1^Molecular and Applied Sciences Laboratory, School of Kinesiology, Auburn University, Auburn, AL, USA

^2^Department of Cell Biology and Physiology, Edward Via College of Osteopathic Medicine – Auburn Campus, Auburn, AL, USA

^3^Lockwood, LLC, Draper, UT 84020, USA

^4^Department of Drug Discovery & Development, Harrison School of Pharmacy, Auburn University Pharmaceutical Research Building, Auburn, AL, USA

Supplementary Western blot images


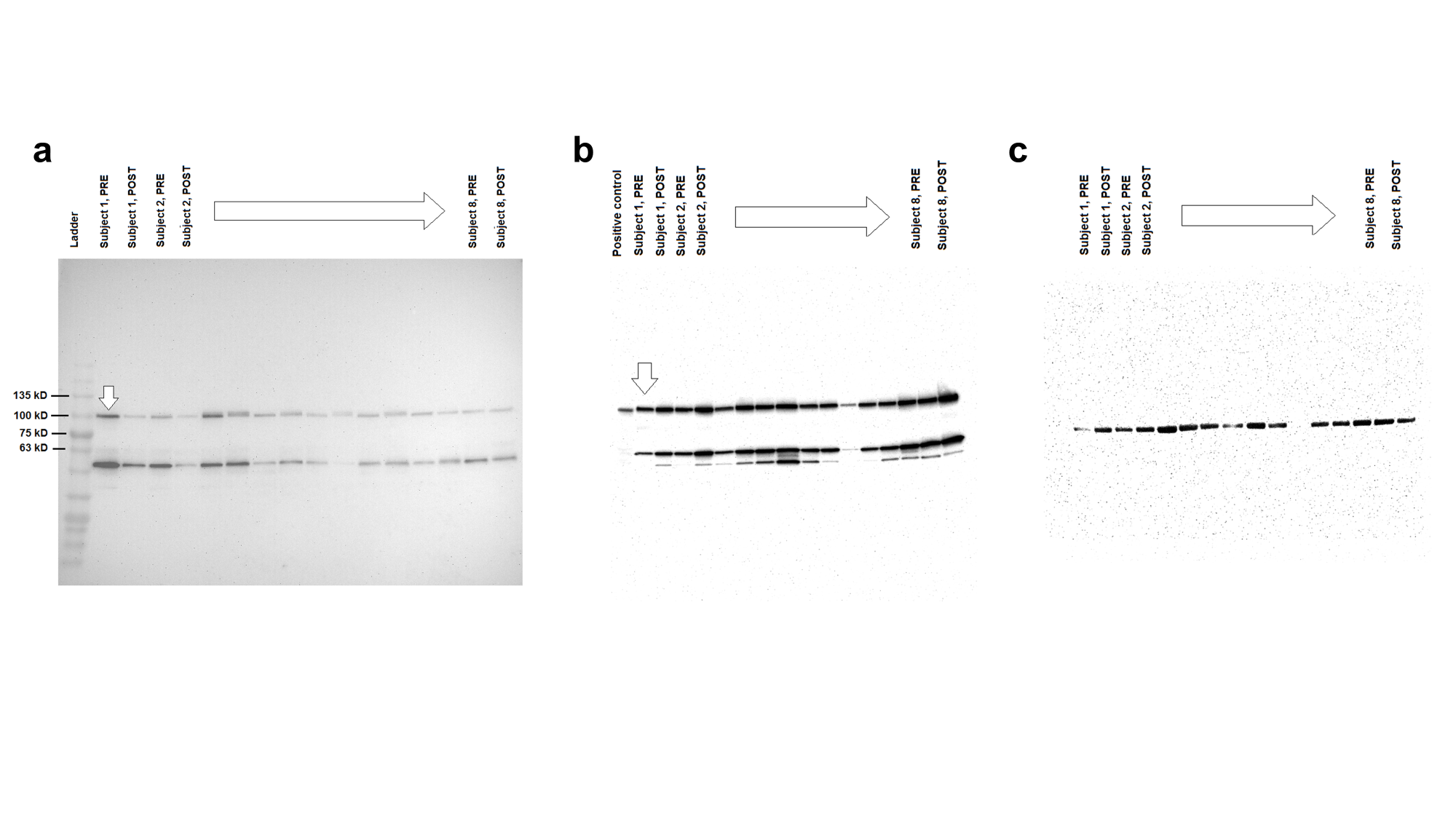


Legend

Panel a, a whole-gel Western blot for androgen receptor. Notably, there were two prominent bands, but the band that was slightly greater than the 100 kD ladder marker was analyzed (indicated by arrow). Panel b, a whole-gel Western blot for estrogen receptor-alpha; again, there were non-specific bands, but our inclusion of a positive control (MCF-7 cell lysate) indicated that the upper band was specific to estrogen receptor alpha and, therefore, was analyzed (indicated by arrow). Panel c, a whole-gel Western blot for estrogen receptor-beta; there were no non-specific bands.

Supplementary muscle histology images

**Pre-training muscle biopsy (20x) (dystrophin/type I/type II/nuclei)**


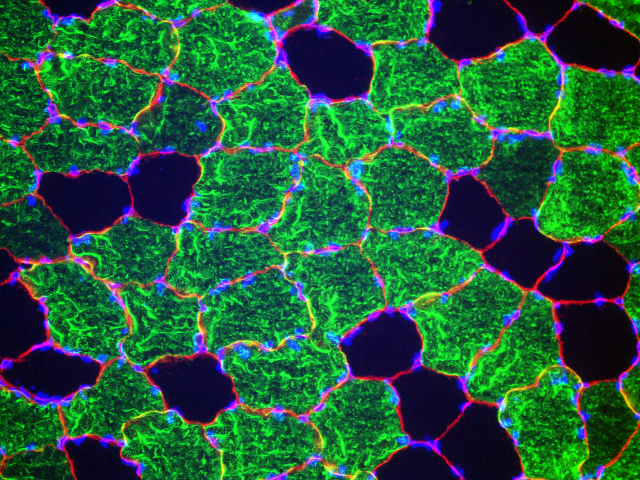


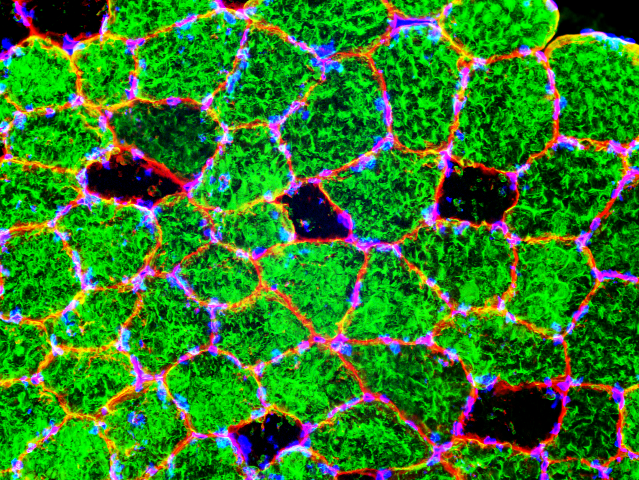


**200 µm**

**Post-training muscle biopsy (20x)**

Supplementary SQ histology images


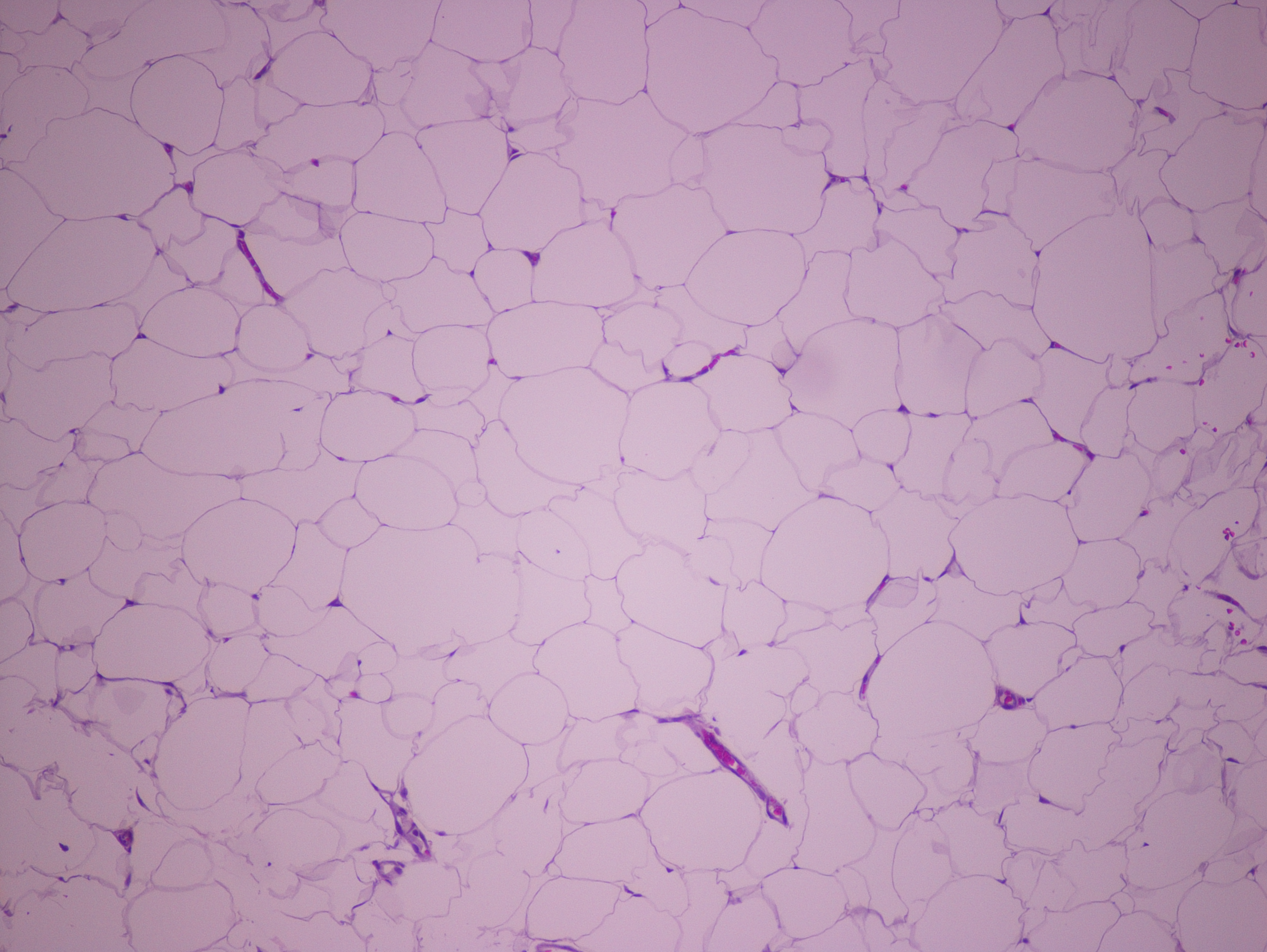


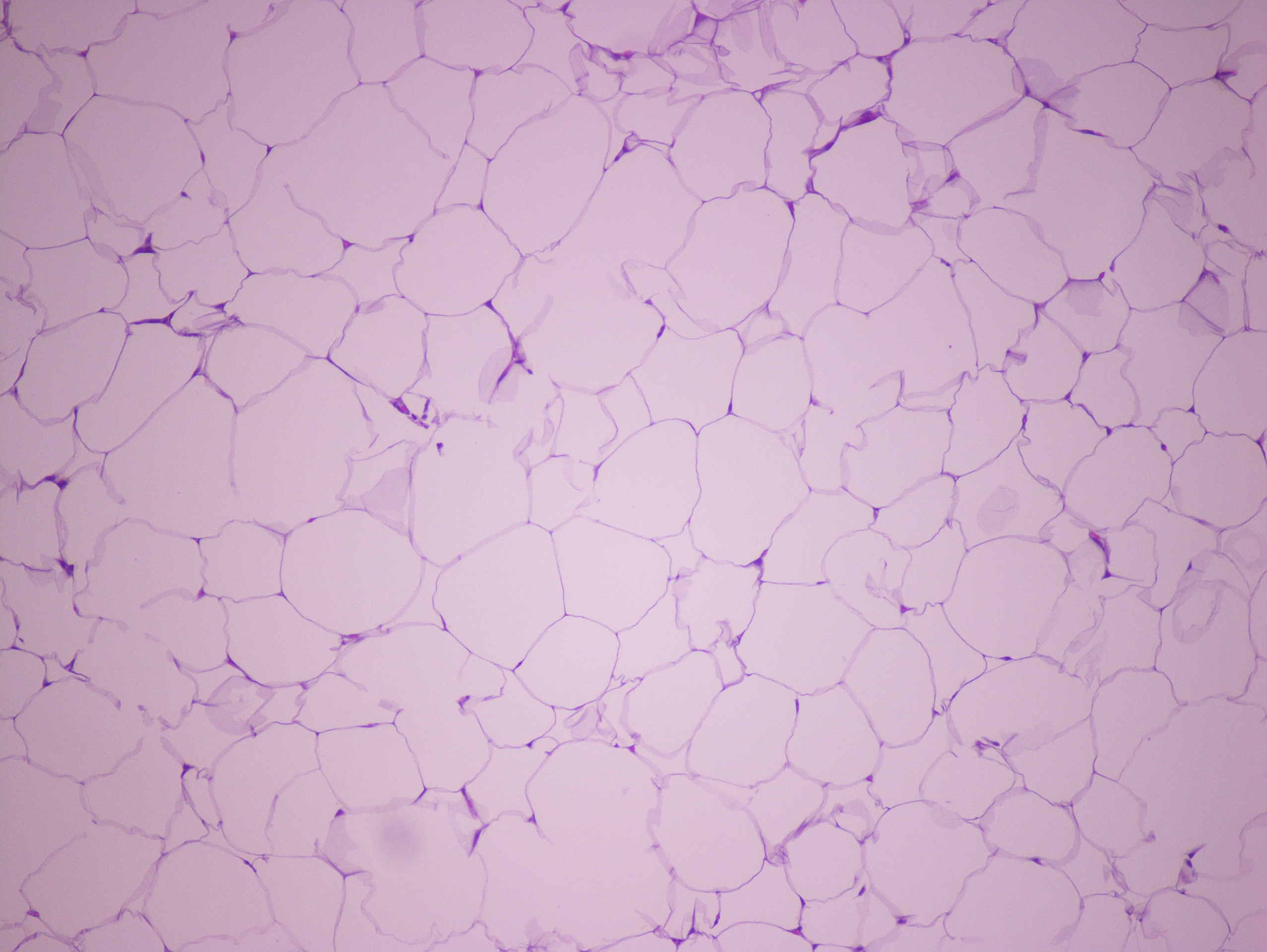


**200 µm**

**Pre-training fat biopsy (10x)**

**Post-training fat biopsy (10x)**

Supplementary Tables

Table 1. Serum Estradiol (pg/ml)

|  | PRE | POST |  |  |  |
| --- | --- | --- | --- | --- | --- |
| Group | Mean ± SD | Mean ± SD | Mean Diff. | Effect Size (Cohen’s *d*) | 95 % CI of Mean Diff. |
| PLA  (n=12) | 49.54 ± 46.38 | 42.58 ± 38.58 | -6.96 | -0.19 | -27.38 – 13.46 |
| SPC  (n=11) | 35.30 ± 18.40 | 32.78 ± 12.11 | -2.52 | -0.07 | -23.85 – 18.81 |
| WPC  (n=9) | 52.56 ± 44.06 | 60.24 ± 75.24 | 7.68 | 0.21 | -15.90 – 31.26 |

Table 2. Serum Testosterone (ng/dl)

|  | PRE | POST |  |  |  |
| --- | --- | --- | --- | --- | --- |
| Group | Mean ± SD | Mean ± SD | Mean Diff. | Effect Size  (Cohen’s *d*) | 95 % CI of Mean Diff |
| PLA  (n=9) | 646.41 ± 250.04 | 695.62 ± 234.77 | 49.21 | 0.15 | -166.41 – 264.83 |
| SPC  (n=13) | 679.77 ± 414.30 | 666.33 ± 435.51 | -13.44 | -0.04 | -192.85 – 165.97 |
| WPC  (n=12) | 622.07 ± 296.28 | 828.68 ± 380.18 | 206.61 | 0.63 | 19.88 – 393.34 |

Table 3. Skeletal Muscle Androgen Receptor Protein Expression

|  | Fold-Change |  |  |  |
| --- | --- | --- | --- | --- |
| Group | Mean ± SD | Mean Diff. | Effect Size (Cohen’s *d*) | 95 % CI of Mean Diff |
| PLA  (n=12) | 1.25 ± 1.12 | 0.25 | 0.29 | 0.25 – 0.73 |
| SPC  (n=15) | 0.72 ± 0.45 | -0.28 | -0.33 | -0.28 – 0.15 |
| WPC  (n=17) | 0.87 ± 0.86 | -0.13 | -0.15 | -0.13 – 0.27 |

Table 4. Adipose Tissue Estrogen Receptor Alpha Protein Expression

|  | Fold-Change |  |  |  |
| --- | --- | --- | --- | --- |
| Group | Mean ± SD | Mean Diff. | Effect Size (Cohen’s *d*) | 95 % CI of Mean Diff |
| PLA  (n=11) | 1.31 ± 0.70 | 0.31 | 0.42 | -0.13 – 0.75 |
| SPC  (n=12) | 1.47 ± 0.92 | 0.47 | 0.64 | 0.05 – 0.89 |
| WPC  (n=8) | 1.38 ± 0.51 | 0.38 | 0.51 | -0.13 – 0.89 |

Table 5. Adipose Tissue Estrogen Receptor Beta Protein Expression

|  | Fold-Change |  |  |  |
| --- | --- | --- | --- | --- |
| Group | Mean ± SD | Mean Diff. | Effect Size (Cohen’s *d*) | 95 % CI of Mean Diff |
| PLA  (n=11) | 1.30 ± 0.89 | 0.3 | 0.09 | -1.63 – 2.23 |
| SPC  (n=12) | 3.47 ± 7.18 | 2.47 | 0.76 | 0.62 – 4.32 |
| WPC  (n=8) | 1.06 ± 0.53 | 0.06 | 0.02 | -2.21 – 2.33 |

Table 6. Adipose Tissue FASN mRNA

|  | Fold-Change |  |  |  |
| --- | --- | --- | --- | --- |
| Group | Mean ± SD | Mean Diff. | Effect Size (Cohen’s *d*) | 95 % CI of Mean Diff |
| PLA  (n=11) | 1.68 ± 0.97 | 0.68 | 0.44 | -0.23 – 1.59 |
| SPC  (n=10) | 1.61 ± 1.36 | 0.61 | 0.40 | -0.34 – 1.56 |
| WPC  (n=9) | 1.48 ± 2.45 | 0.48 | 0.31 | -0.53 – 1.49 |

Table 7. Adipose Tissue HSL mRNA

|  | Fold-Change |  |  |  |
| --- | --- | --- | --- | --- |
| Group | Mean ± SD | Mean Diff. | Effect Size (Cohen’s *d*) | 95 % CI of Mean Diff |
| PLA  (n=11) | 2.34 ± 2.39 | 1.34 | 0.74 | 0.26 – 2.42 |
| SPC  (n=10) | 1.66 ± 1.49 | 0.66 | 0.36 | -0.47 – 1.79 |
| WPC  (n=9) | 1.90 ± 1.48 | 0.9 | 0.49 | -0.29 – 2.09 |

Table 8. Adipose Tissue PPARG mRNA

|  | Fold-Change |  |  |  |
| --- | --- | --- | --- | --- |
| Group | Mean ± SD | Mean Diff. | Effect Size (Cohen’s *d*) | 95 % CI of Mean Diff |
| PLA  (n=11) | 0.75 ± 0.26 | -0.25 | -0.29 | -0.75 – 0.25 |
| SPC  (n=10) | 1.04 ± 1.04 | 0.04 | 0.05 | -0.49 – 0.57 |
| WPC  (n=9) | 1.10 ± 1.38 | 0.10 | 0.12 | -0.46 – 0.66 |

Table 9. Adipose Tissue SREBP1 mRNA

|  | Fold-Change |  |  |  |
| --- | --- | --- | --- | --- |
| Group | Mean ± SD | Mean Diff. | Effect Size (Cohen’s *d*) | 95 % CI of Mean Diff |
| PLA  (n=11) | 1.19 ± 0.93 | 0.19 | 0.06 | -1.82 – 2.20 |
| SPC  (n=10) | 2.92 ± 5.28 | 1.92 | 0.56 | -0.19 – 4.03 |
| WPC  (n=9) | 2.66 ± 4.40 | 1.66 | 0.49 | -0.57 – 3.89 |

Table 10. Adipose Tissue ADRA2A mRNA

|  | Fold-Change |  |  |  |
| --- | --- | --- | --- | --- |
| Group | Mean ± SD | Mean Diff. | Effect Size (Cohen’s *d*) | 95 % CI of Mean Diff |
| PLA  (n=11) | 1.28 ± 1.46 | 0.28 | 0.20 | -0.53 – 1.09 |
| SPC  (n=10) | 1.19 ± 0.74 | 0.19 | 0.14 | -0.66 – 1.04 |
| WPC  (n=9) | 1.78 ± 1.98 | 0.78 | 0.57 | -0.12 – 1.68 |

Table 11. Skeletal Muscle AR mRNA

|  | Fold-Change |  |  |  |
| --- | --- | --- | --- | --- |
| Group | Mean ± SD | Mean Diff. | Effect Size (Cohen’s *d*) | 95 % CI of Mean Diff |
| PLA  (n=10) | 1.16 ± 0.67 | 0.16 | 0.05 | -1.78– 2.10 |
| SPC  (n=9) | 1.89 ± 2.01 | 0.89 | 0.28 | -1.15 – 2.93 |
| WPC  (n=15) | 2.84 ± 5.35 | 1.84 | 0.59 | 0.26 – 3.42 |

Table 12. Skeletal Muscle Atrogin-1 mRNA

|  | Fold-Change |  |  |  |
| --- | --- | --- | --- | --- |
| Group | Mean ± SD | Mean Diff. | Effect Size (Cohen’s *d*) | 95 % CI of Mean Diff |
| PLA  (n=10) | 1.05 ± 0.53 | 0.05 | 0.10 | -0.27 – 0.37 |
| SPC  (n=9) | 0.98 ± 0.24 | -0.02 | -0.04 | -0.36 – 0.32 |
| WPC  (n=15) | 1.20 ± 0.68 | 0.20 | 0.38 | -0.06 – 0.46 |

Table 13. Skeletal Muscle IGF-1 mRNA

|  | Fold-Change |  |  |  |
| --- | --- | --- | --- | --- |
| Group | Mean ± SD | Mean Diff. | Effect Size (Cohen’s *d*) | 95 % CI of Mean Diff |
| PLA  (n=10) | 1.40 ± 1.69 | 0.40 | 0.53 | -0.07 – 0.87 |
| SPC  (n=9) | 0.80 ± 0.33 | -0.20 | -0.26 | -0.69 – 0.29 |
| WPC  (n=15) | 0.78 ± 0.40 | -0.22 | -0.29 | -0.60 – 0.16 |

Table 14. Skeletal Muscle MSTN mRNA

|  | Fold-Change |  |  |  |
| --- | --- | --- | --- | --- |
| Group | Mean ± SD | Mean Diff. | Effect Size (Cohen’s *d*) | 95 % CI of Mean Diff |
| PLA  (n=10) | 1.42 ± 0.82 | 0.42 | 0.70 | 0.05– 0.79 |
| SPC  (n=9) | 0.97 ± 0.51 | -0.03 | -0.05 | -0.42 – 0.36 |
| WPC  (n=15) | 0.95 ± 0.51 | -0.05 | -0.08 | -0.35 – 0.25 |

Table 15. Skeletal Muscle MYOG mRNA

|  | Fold-Change |  |  |  |
| --- | --- | --- | --- | --- |
| Group | Mean ± SD | Mean Diff. | Effect Size (Cohen’s *d*) | 95 % CI of Mean Diff |
| PLA  (n=10) | 0.72 ± 0.26 | -0.28 | -0.56 | -0.59 – 0.03 |
| SPC  (n=9) | 0.84 ± 0.34 | -0.16 | -0.32 | -0.48 – 0.16 |
| WPC  (n=15) | 1.14 ± 0.74 | -0.14 | 0.28 | -0.11 – 0.39 |

Table 16. Skeletal Muscle ODC1 mRNA

|  | Fold-Change |  |  |  |
| --- | --- | --- | --- | --- |
| Group | Mean ± SD | Mean Diff. | Effect Size (Cohen’s *d*) | 95 % CI of Mean Diff |
| PLA  (n=10) | 1.06 ± 1.39 | 0.06 | 0.07 | -0.48 – 0.60 |
| SPC  (n=9) | 0.99 ± 0.63 | -0.01 | -0.01 | -0.58 – 0.56 |
| WPC  (n=15) | 0.82 ± 0.68 | -0.18 | -0.21 | -0.62 – 0.26 |

Table 17. Body Mass (kg)

|  | PRE | POST |  |  |  |
| --- | --- | --- | --- | --- | --- |
| Group | Mean ± SD | Mean ± SD | Mean Diff. | Effect Size (Cohen’s *d*) | 95 % CI of Mean Diff |
| PLA  (n=13) | 78.00 ± 10.29 | 81.48 ± 9.83 | 3.48 | 0.28 | 3.01 — 3.95 |
| SPC  (n=13) | 81.37 ± 13.84 | 84.13 ± 14.50 | 2.76 | 0.22 | 2.29 — 3.23 |
| WPC  (n=15) | 79.57 ± 12.94 | 81.83 ± 12.84 | 2.26 | 0.18 | 1.82 — 2.70 |

Table 18. Fat Mass (kg)

|  | PRE | POST |  |  |  |
| --- | --- | --- | --- | --- | --- |
| Group | Mean ± SD | Mean ± SD | Mean Diff. | Effect Size (Cohen’s *d*) | 95 % CI of Mean Diff |
| PLA  (n=15) | 18.06 ± 5.19 | 18.8 ± 5.38 | 0.74 | 0.10 | 0.30 — 1.18 |
| SPC  (n=15) | 19.73 ± 8.45 | 20.51 ± 8.51 | 0.78 | 0.10 | 0.34 — 1.22 |
| WPC  (n=17) | 19.47 ± 8.58 | 18.69 ± 8.25 | -0.78 | -0.10 | -1.20 — -0.36 |

Table 19. Total Lean Mass (kg)

|  | PRE | POST |  |  |  |
| --- | --- | --- | --- | --- | --- |
| Group | Mean ± SD | Mean ± SD | Mean Diff. | Effect Size (Cohen’s *d*) | 95 % CI of Mean Diff |
| PLA  (n=13) | 57.05 ± 6.33 | 59.90 ± 6.22 | 2.85 | 0.46 | 2.38 — 3.32 |
| SPC  (n=13) | 57.67 ± 6.76 | 60.42 ± 7.35 | 2.75 | 0.44 | 2.28 — 3.22 |
| WPC  (n=15) | 58.15 ± 5.74 | 60.81 ± 5.33 | 2.66 | 0.43 | 2.22 — 3.10 |

Table 20. Visceral Adiposity (kg)

|  | PRE | POST |  |  |  |
| --- | --- | --- | --- | --- | --- |
| Group | Mean ± SD | Mean ± SD | Mean Diff. | Effect Size (Cohen’s *d*) | 95 % CI of Mean Diff |
| PLA  (n=15) | 0.27 ± 0.20 | 0.26 ± 0.21 | -0.01 | -0.03 | -0.45 — 0.43 |
| SPC  (n=15) | 0.42 ± 0.37 | 0.44 ± 0.48 | 0.02 | 0.06 | -0.42 — 0.46 |
| WPC  (n=17) | 0.48 ± 0.35 | 0.45 ± 0.32 | -0.03 | -0.10 | -0.45 — 0.39 |

Table 21. Android Adiposity (kg)

|  | PRE | POST |  |  |  |
| --- | --- | --- | --- | --- | --- |
| Group | Mean ± SD | Mean ± SD | Mean Diff. | Effect Size (Cohen’s *d*) | 95 % CI of Mean Diff |
| PLA  (n=15) | 1.35 ± 0.58 | 1.4 ± 0.66 | 0.05 | 0.06 | -0.39 — 0.49 |
| SPC  (n=15) | 1.55 ± 0.89 | 1.60 ± 0.95 | 0.05 | 0.06 | -0.39 — 0.49 |
| WPC  (n=17) | 1.52 ± 0.90 | 1.42 ± 0.87 | -0.10 | -0.13 | -0.52 — 0.32 |

Table 22. Gynoid Adiposity (kg)

|  | PRE | POST |  |  |  |
| --- | --- | --- | --- | --- | --- |
| Group | Mean ± SD | Mean ± SD | Mean Diff. | Effect Size (Cohen’s *d*) | 95 % CI of Mean Diff |
| PLA  (n=15) | 3.23 ± 0.97 | 3.31 ± 0.96 | 0.08 | 0.06 | -0.36 — 0.52 |
| SPC  (n=15) | 3.42 ± 1.59 | 3.49 ± 1.46 | 0.07 | 0.05 | -0.37 — 0.51 |
| WPC  (n=17) | 3.39 ± 1.60 | 3.1 ± 1.64 | -0.29 | -0.21 | -0.71 — 0.13 |

Table 23. aCSA (μm^2^)

|  | PRE | POST |  |  |  |
| --- | --- | --- | --- | --- | --- |
| Group | Mean ± SD | Mean ± SD | Mean Diff. | Effect Size (Cohen’s *d*) | 95 % CI of Mean Diff |
| PLA  (n=14) | 3172.89 ± 1268.45 | 2765.55 ± 1680.46 | -407.34 | -0.34 | -407.80 — -406.88 |
| SPC  (n=13) | 3540.94 ± 1405.45 | 3340.39 ± 1586.78 | -200.55 | -0.17 | -201.02 — -200.08 |
| WPC  (n=14) | 3819.91 ± 965.70 | 3552.34 ± 1027.82 | -267.57 | -0.22 | -268.03 — -267.11 |

Table 24. VL Thickness (cm)

|  | PRE | POST |  |  |  |
| --- | --- | --- | --- | --- | --- |
| Group | Mean ± SD | Mean ± SD | Mean Diff. | Effect Size (Cohen’s *d*) | 95 % CI of Mean Diff |
| PLA  (n=13) | 2.26 ± 0.30 | 2.72 ± 0.32 | 0.46 | 1.49 | -0.01 — 0.93 |
| SPC  (n=13) | 2.62 ± 0.41 | 3.04 ± 0.34 | 0.42 | 1.36 | -0.05 — 0.89 |
| WPC  (n=15) | 2.49 ± 0.23 | 2.96 ± 0.29 | 0.47 | 1.56 | 0.03 — 0.91 |

Table 25. Dual Leg Lean Mass (kg)

|  | PRE | POST |  |  |  |
| --- | --- | --- | --- | --- | --- |
| Group | Mean ± SD | Mean ± SD | Mean Diff. | Effect Size (Cohen’s *d*) | 95 % CI of Mean Diff |
| PLA  (n=13) | 21.90 ± 2.81 | 23.43 ± 2.86 | 1.53 | 0.56 | 1.06 — 2.00 |
| SPC  (n=13) | 22.46 ± 2.85 | 23.90 ± 1.44 | 1.44 | 0.53 | 0.97 — 1.91 |
| WPC  (n=15) | 22.55 ± 2.59 | 23.93 ± 2.32 | 1.38 | 0.50 | 0.94 — 1.82 |

Table 26. Type I fCSA (μm^2^)

|  | PRE | POST |  |  |  |
| --- | --- | --- | --- | --- | --- |
| Group | Mean ± SD | Mean ± SD | Mean Diff. | Effect Size (Cohen’s *d*) | 95 % CI of Mean Diff |
| PLA  (n=13) | 3449.24 ± 975.28 | 3971.24 ± 1143.75 | 522.00 | 0.58 | 521.53 — 522.47 |
| SPC  (n=14) | 3937.71 ± 864.35 | 4698.13 ± 1329.59 | 760.42 | 0.84 | 759.96 — 760.88 |
| WPC  (n=15) | 3583.41 ± 870.45 | 3975.84 ± 1069.78 | 392.43 | 0.44 | 391.99 — 392.87 |

Table 27. Type II fCSA (μm^2^)

|  | PRE | POST |  |  |  |
| --- | --- | --- | --- | --- | --- |
| Group | Mean ± SD | Mean ± SD | Mean Diff. | Effect Size (Cohen’s *d*) | 95 % CI of Mean Diff |
| PLA  (n=13) | 4658.66 ± 1110.54 | 5093.30 ± 1116.52 | 434.64 | 0.37 | 434.17 —435.12 |
| SPC  (n=14) | 4535.50 ± 967.69 | 5182.24 ± 1422.97 | 646.74 | 0.55 | 646.28 — 647.19 |
| WPC  (n=15) | 4760.92 ± 1415.50 | 6287.67 ± 2348.93 | 1526.74 | 1.30 | 1526.30 — 1527.19 |

Table 28. Sample sizes for each analysis

|  | PLA | SPC | WPC | Total |
| --- | --- | --- | --- | --- |
| Age (n=) | 13 | 13 | 15 | 41 |
| Total Mass (n=) | 13 | 13 | 15 | 41 |
| Total Fat Mass (n=) | 15 | 15 | 17 | 47 |
| Total Body Muscle Mass (n=) | 13 | 13 | 15 | 41 |
| Visceral Adiposity (n=) | 15 | 15 | 17 | 47 |
| Android Adiposity (n=) | 15 | 15 | 17 | 47 |
| Gynoid Adiposity (n=) | 15 | 15 | 17 | 47 |
| aCSA (n=) | 14 | 13 | 14 | 41 |
| VL Thickness (n=) | 13 | 13 | 15 | 41 |
| Dual Leg Lean Mass (n=) | 13 | 13 | 15 | 41 |
| Type I fCSA (n=) | 13 | 13 | 15 | 41 |
| Type II fCSA (n=) | 13 | 13 | 15 | 41 |

Table 29. Supplement Nutrition Facts

|  | **PLA** | **WPC** | **SPC** |
| --- | --- | --- | --- |
| Calories | 204 | 184 | 266 |
| Total Fat (g) | 2.8 | 3.5 | 4.5 |
| Saturated Fat (g) | 2.3 | 2.3 | 2.6 |
| Trans Fat (g) | 0.0 | 0.1 | 0.2 |
| Cholesterol (mg) | 3.8 | 74.0 | 5.3 |
| Total Carbohydrate (g) | 44.4 | 12.0 | 17.2 |
| Dietary Fiber (g) | 1.6 | 1.8 | 1.5 |
| Sugars (g) | 6.0 | 5.9 | 6.2 |
| Protein (g) | 0.4 | 26.3 | 39.2 |
| Vitamins and Minerals |  |  |  |
| Calcium (mg) | 15 | 155 | 165 |
| Iron (mg) | 0.38 | 0.63 | 5.21 |
| Potassium (mg) | 32 | 230 | 961 |
| Sodium (mg) | 91 | 133 | 217 |
| Vitamin D3 (IU) | 0.0 | 0.0 | 0.0 |
| Amino Acid Content |  |  |  |
| Alanine (mg) | 7 | 1,397 | 1,646 |
| Arginine (mg) | 8 | 766 | 2,969 |
| Aspartic Acid (mg) | 15 | 2,881 | 4,537 |
| Cystine (mg) | 0 | 651 | 536 |
| Glutamic Acid (mg) | 36 | 4,530 | 7,154 |
| Glycine (mg) | 6 | 489 | 1,597 |
| Histidine (mg) | 0 | 470 | 910 |
| Isoleucine (mg) | 8 | 1,736 | 1,842 |
| Leucine (mg) | 15 | 2,794 | 2,960 |
| Lysine (mg) | 11 | 2,386 | 2,362 |
| Methionine (mg) | 0 | 598 | 540 |
| Phenylalanine (mg) | 8 | 861 | 1980 |
| Proline (mg) | 14 | 1,630 | 2,029 |
| Serine (mg) | 10 | 1,348 | 1,950 |
| Threonine (mg) | 7 | 1,853 | 1,499 |
| Tryptophan | 0 | 482 | 501 |
| Tyrosine (mg) | 7 | 808 | 1480 |
| Valine (mg) | 11 | 1,465 | 1754 |
| Total EAAs (mg) | 60 | 12,645 | 14,348 |
| Total BCAAs (mg) | 34 | 5,995 | 6,556 |
| Degree of hydrolysis (%) | N/A | N/A | N/A |
| Molecular weight profile (%) |  |  |  |
| >20 kD | - | 0.13 | - |
| 10-20 kD | - | 64.97 | - |
| 5-10 kD | - | 20.25 | - |
| 2-5 kD | - | 8.56 | - |
| 1-2 kD | - | 1.49 | - |
| 0.5-1 kD | - | 0.71 | - |
| <0.5 kD | - | 3.90 | - |
| Average molecular weight (kD) | - | 14.28 | - |

Abbreviations: PLA, maltodextrin placebo; WPC, whey protein concentrate; SPC, soy protein concentrate; g, grams; mg, milligrams; IU, international units; kD, kilodaltons.
